# Fat digestion using RELiZORB in children with short bowel syndrome who are dependent on parenteral nutrition: Protocol for a 90-day, phase 3, open labeled study

**DOI:** 10.1371/journal.pone.0282248

**Published:** 2023-03-01

**Authors:** Savas T. Tsikis, Scott C. Fligor, Paul D. Mitchell, Thomas I. Hirsch, Sarah Carbeau, Eric First, Greta Loring, Coral Rudie, Steven D. Freedman, Camilia R. Martin, Kathleen M. Gura, Mark Puder

**Affiliations:** 1 Vascular Biology Program, Boston Children’s Hospital, Harvard Medical School, Boston, Massachusetts, United States of America; 2 Department of Surgery, Boston Children’s Hospital, Harvard Medical School, Boston, Massachusetts, United States of America; 3 Institutional Centers for Clinical and Translational Research, Boston Children’s Hospital, Boston, Massachusetts, United States of America; 4 Alcresta Therapeutics, Newton, Massachusetts, United States of America; 5 Division of Gastroenterology, Hepatology, and Nutrition, Boston Children’s Hospital, Harvard Medical School, Boston, Massachusetts, United States of America; 6 Division of Gastroenterology, Beth Israel Deaconess Medical Center, Boston, Massachusetts, United States of America; 7 Division of Neonatology, Weill Cornell Medicine, New York City, New York, United States of America; 8 Department of Pharmacy, Boston Children’s Hospital, Harvard Medical School, Boston, Massachusetts, United States of America; UNITED KINGDOM

## Abstract

**Background:**

Short bowel syndrome (SBS) is a leading cause of intestinal failure resulting in parenteral nutrition (PN) dependence and nutritional deficiencies. Long-term PN use is associated with the development of sepsis and intestinal failure-associated liver disease. Achieving enteral autonomy is the optimal way to prevent these complications. In SBS, the decreased intestinal length, bile acid deficiency, and rapid transit time contribute to fat malabsorption and continued PN dependence. We propose the use of an immobilized lipase cartridge (ILC; RELiZORB) that connects in-line with enteral feed tubing sets and is designed to breakdown the majority of fats provided in enteral nutrition (EN). Preclinical studies have demonstrated both improved fat and fat-soluble vitamin absorption with ILC use in a porcine model of SBS. To evaluate the clinical applicability of these findings, we designed a phase 3, open labeled, single center, clinical trial to determine the safety, tolerability, and efficacy of the RELiZORB enzyme cartridge when used daily with EN for 90 days.

**Methods:**

The patient population will include PN dependent children with SBS, aged 2–18 years. The primary outcome is the change in PN calories from baseline, assessed weekly throughout the study. Changes in growth Z-scores, 72-hour fecal fat and coefficient of fat absorption, plasma fatty acids and fat-soluble vitamins will also be evaluated. Assessment of change in continuous outcomes will be made using the area under the curve, expressed as a percent change relative to baseline, calculated over study day 7 to 90 (AUC_7-90_). The incidence of adverse events will be monitored and summarized by system organ class.

**Discussion:**

If successful, RELiZORB may offer a safe alternative to reducing PN dependence and achieving enteral autonomy in pediatric intestinal failure. These results would be clinically significant given the clear association between long-term PN use and complications in SBS.

**Trial registration:**

ClinicalTrials.gov NCT03530852; registered on May 21^st^, 2018, last update posted on September 14^th^, 2022.

## Introduction

### Background

Short bowel syndrome (SBS) is a leading cause of intestinal failure (IF) worldwide, affecting both children and adults [1, 2]. In children, the most common causes include necrotizing enterocolitis, intestinal atresia, and segmental volvulus [3]. Regardless of the exact etiology, SBS-IF is characterized by the functional or anatomical loss of extensive segments of intestine. This ultimately compromises the digestive and absorptive processes that are necessary to support growth and development. As a result, patients frequently require extended periods of parenteral nutrition (PN) and enteral nutrition (EN) for their survival.

Long-term PN use is associated with numerous life-limiting complications. PN delivery requires indwelling central venous catheters (CVCs), which can lead to central line-associated bloodstream infections (CLABSIs) and sepsis. It is estimated that among patients with CVCs for PN use, there are approximately 1.6–8.9 CLABSI episodes per 1000 catheter-days [2, 4, 5]. PN-dependent SBS patients are also at risk for intestinal failure-associated liver disease (IFALD). The incidence of IFALD in children may be as high as 50% and is directly proportional to the duration of PN [6]. Importantly, IFALD may ultimately progress to liver fibrosis and cirrhosis in a subset of patients on prolonged PN contributing to mortality [7].

The primary goal in the management of SBS is to advance enteral feeds and reduce PN dependence while maintaining a stable growth trajectory and nutritional status [8]. Teduglutide, a glucagon-like peptide-2 (GLP-2) analogue, is approved by the Food and Drug Administration (FDA) for the treatment of SBS based on studies in children and adults demonstrating efficacy in reducing parenteral support and intravenous fluid requirements [9, 10]. Use of this medication is not without side effects, and as teduglutide enhances mucosal proliferation, regular endoscopy is necessary to screen for adenomatous polyps and malignancy. Furthermore, even after complete weaning of PN and achieving enteral autonomy, SBS patients remain at risk for micronutrient and fat-soluble vitamin deficiencies, requiring frequent biochemical monitoring [11]. Novel therapeutic strategies are thus needed to address these issues and improve patient outcomes.

Major limiting factors in achieving enteral autonomy and reducing PN dependence include fat malabsorption and associated nutritional deficiencies [8]. Numerous biochemical reactions are required for fat absorption to occur including bile salt emulsification, pancreatic lipase-mediated triglyceride breakdown, and micelle formation. In SBS, several factors such as decreased intestinal length, relative bile acid deficiency, and increased transit time impact efficient fat absorption [12]. We hypothesized that by using an external lipase device to pre-digest fats, nutritional absorption would improve, thereby contributing to reduced PN dependence in patients with SBS. RELiZORB (Alcresta Therapeutics, Newton, MA) is a commercially available FDA-cleared immobilized lipase cartridge which connects in-line with existing enteral feeding sets and is designed to mimic the function of pancreatic lipase. As EN passes through the device, lipase enzymes which are covalently bound to microbeads, hydrolyze triglyceride fats to deliver readily absorbable fatty acids and monoglycerides [13].

We have recently shown in a large animal model that the use of an immobilized lipase cartridge improved both fat and fat-soluble vitamin absorption [14]. To evaluate if these findings translate clinically, we propose to conduct a phase 3, open labeled study of RELiZORB in pediatric patients with SBS who are dependent on PN. If successful, RELiZORB could be considered an important supplemental therapeutic to improve clinical outcomes and achieve enteral autonomy in pediatric intestinal failure.

### Objectives

This protocol complies with the SPIRIT reporting guidelines (S1 File). The primary objective of this study is to evaluate the effect of the RELiZORB enzyme cartridge when used with EN daily for 90 days on the change from baseline in PN calories. The secondary objective is to evaluate the effect of RELiZORB on the change from baseline in body weight using weight adjusted for age Z-scores. Measurements will be assessed at baseline and on study days 7, 14, 28, 60, 90 for in-clinic assessments, and weekly for at-home assessments in pediatric subjects with SBS, aged 2 years– 18 years, who are dependent on PN.

Safety objectives for the study include the incidence of grade 2 or higher adverse events (AEs), abnormal vital signs, and abnormalities in hematology and biochemistry parameters based on in-clinical laboratory evaluations. Several exploratory objectives will also be examined including: change (from baseline) in the coefficient of fat absorption (CFA) measured through 72-hour stool collection, plasma and stool fatty acid composition, PN volume, total enteral calories/volume, and total oral calories/volume. In addition to weight measurements, growth over the study period will be assessed through height, head circumference (in children <36 months of age), and BMI measurements.

## Materials and methods

### Participants, interventions, and outcomes

#### Trial design, setting, and explanation for choice of comparators

The study is a phase 3 open label, pre- and post-, single center clinical trial in pediatric subjects with SBS, aged 2–18 years who have been dependent on PN for at least six months. Treatment will be the use of the RELiZORB enzyme cartridge with the administration of EN daily for a total of 90 days. This study will be open label; all patients receive the study treatment. Allocation will be known by the subjects, Principal Investigator and their medical staff. Participants will be enrolled and followed at Boston Children’s Hospital.

Based on the study design, each subject will be compared to themselves utilizing pre-treatment baseline measurements; effectively each patient serves as their own control. This type of study design is beneficial in rare diseases, such as pediatric SBS, characterized by extensive heterogeneity between patients [3]. Given this approach and to reduce bias, patients need to demonstrate stability on key outcomes prior to enrollment, including PN dependence and EN requirements, as outlined below.

#### Eligibility criteria

The following are the inclusion criteria required for eligibility:

1. Male or female patients, aged 2–18 years, inclusive.
2. Diagnosed with SBS, as determined by medical history and PN dependence (i.e. need for PN for >60 days after intestinal resection or a bowel length <25% of expected).
3. Congenital or acquired gastrointestinal disease requiring surgical intervention that has occurred at least three months prior to screening.
4. Patient is on parenteral lipid and at least 30% of daily caloric and fluid intake has been provided by PN for at least six months prior to screening.
5. Stable PN nutrition requirement, determined by less than 5% reduction in PN nutrition for at least one month prior to screening, or at the discretion of the investigator.
6. The patient has a CVC at the time of study inclusion.
7. Screening direct bilirubin that is in the normal range for age or is not determined to be clinically significant by the investigator.
8. The patient has an existing feeding tube, receiving EN via an enteral feeding pump at a rate>10ml/hr but <120ml/hr, and is able to tolerate at least 10 ml/kg/day EN with which the RELiZORB cartridge can be used.
9. Stable EN requirement with no change in formula composition or rate for at least one month prior to screening.
10. The patient or a parent or legal guardian of the patient is able to read, understand, and is willing to provide informed consent (or assent, if applicable) for the patient.
11. The patient (if assent is applicable) or a parent or legal guardian is able to understand the requirements of the study and is willing to bring the patient to all clinic visits and complete all study related procedures (as determined by the investigator).
12. A parent or legal guardian is willing to provide written authorization for the use and disclosure of protected health information.

Patients will be excluded from the study if any of the following apply:

1. Other causes of chronic liver disease other than SBS (i.e. hepatitis C, cystic fibrosis, biliary atresia, alpha 1 anti-trypsin deficiency, and Alagille syndrome).
2. The patient has had a bowel lengthening procedure, including but not limited to, a STEP procedure.
3. Any serum triglyceride concentration >400 mg/dL at screening.
4. Pancreatic insufficiency as defined as the use of pancreatic enzymes within 30 days prior to screening.
5. Evidence of untreated intestinal obstruction or active stenosis, as determined by the investigator.
6. Unstable absorption due to cystic fibrosis or known DNA abnormalities (i.e., familial adenomatous polyposis, Fanconi syndrome) as determined by the investigator.
7. History of microvillus inclusion disease, as determined by medical history.
8. Severe known dysmotility syndrome (i.e., pseudo-obstruction, gastroschisis-related motility disorders), as determined by the investigator.
9. Initiation of teduglutide or other GLP-2 analogues within six months of screening
10. Use of growth hormone, or supplemental glutamine within three months prior to screening.
11. Use of cisapride within 30 days prior to screening.
12. Active clinically significant pancreatic or biliary disease, as determined by the investigator.
13. Receiving EN via any formula that is not compatible with the RELiZORB cartridge (for example, insoluble fiber-containing formulas).
14. Determined by the investigator to be unsuitable for participation in this trial for any reason.

#### Interventions

**Fig 1:**
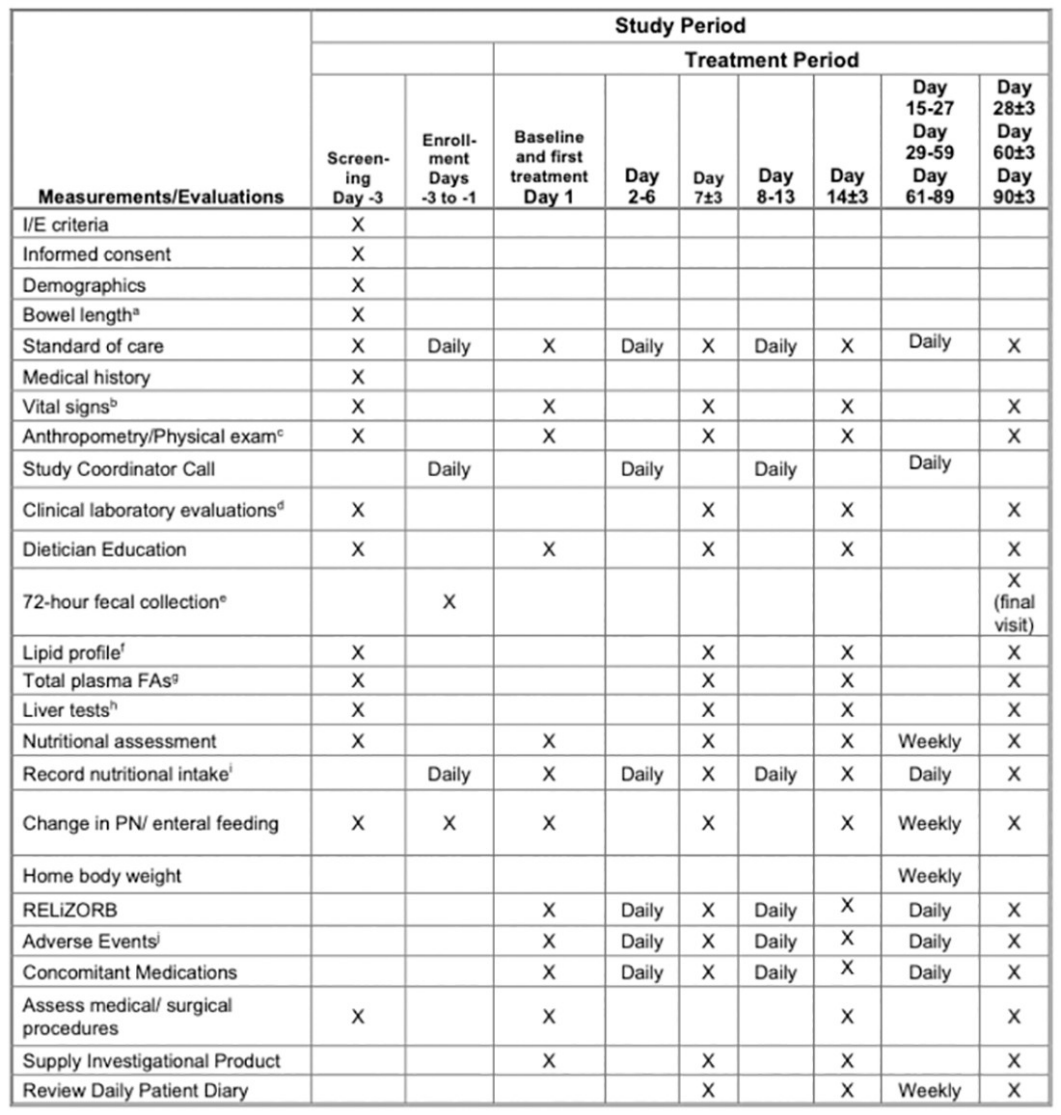
Schedule of enrolment, interventions, and assessments. I/E criteria = Inclusion and exclusion criteria. FAs = fatty acids. PN = Parenteral nutrition. a. Record known bowel length/type from last surgical resection or other measurement. b. Blood pressure, pulse rate, respiratory rate and temperature. c. Includes height or length, head circumference (for subjects < 36 months), and weight. If a physical examination or nutrition assessment was performed during the 3-day screening period, it will be used for the baseline assessment when available. d. Includes chemistry (including fat-soluble Vitamins A, D, E, and K), hematology, coagulation and C-reactive protein (CRP). Clinical laboratory evaluations obtained at time of screening will serve as the baseline assessment. e. 72-hour fecal collection will occur in the 72 hours preceding treatment day 1 and the final clinic visit day 90. f. Samples of the lipid profile will include cholesterol, high-density lipoprotein (HDL), very low-density lipoprotein (VLDL), and low-density lipoprotein (LDL). Results of samples obtained at time of screening will be analyzed during enrollment period, prior to participant receiving device. g. Samples of total plasma fatty acids will include total saturated, total monosaturated, total polyunsaturated, total Omega-3, total Omega 6, total fatty acids, a-linolenic Acid, linoleic acid, oleic acid, EPA, arachidonic acid, mead acid, DHA, and triene:tetraene ratio. h. Serum alanine aminotransferase (ALT), serum aspartate aminotransferase (AST), and serum gamma-glutamyl transpeptidase (GGT). i. 24-hour nutritional intake/composition (PO and enteral dietary intake and PN volume) will be recorded using a phone-based application or paper diary. This will be especially important during the 72-hour stool collection periods for calculation of the coefficient of fat absorption. j. Adverse events will be recorded daily in an electronic or paper format by the subject’s parent or guardian, and by study staff during clinic visits. Monitoring of these events will also occur through a daily phone call from a study coordinator. If any AEs or concerns are noted by the family, the study coordinator will alert the study team by email. PI or other delegated investigators will determine next steps.

Subjects are initially evaluated in a screening visit (Day -3) during which they will be screened for eligibility, including baseline clinical and physical assessments (Fig 1). At screening, the investigator or designee will review the subject’s medical history to determine eligibility, explain the study to the subject and the subject’s parent or legal guardian, and if eligible, obtain informed consent (S3 File). Informed consent and assent will be obtained from study investigators and authorized personnel and the discussion will be documented in the patient’s electronic medical record. If consent is given and the subject is deemed eligible based on the results of the screening visit, they will be enrolled in the study and pre-baseline assessments will be performed, which will include physical examination, nutritional assessment, vital signs, and laboratory evaluations (Fig 1). The subject’s parent or guardian will also receive education from a registered dietitian on recording caloric intake and composition in oral and enteral feeds, and PN. As part of this education, the dietitian will review how to record daily oral and enteral intake via paper dairy or with a HIPAA-compliant nutrition/food tracking phone application. If the latter, the subject’s parent or guardian will receive access to a nutrition/food tracking phone application.

The subject’s parent or guardian will then be asked to collect all stool for 72-hours over Day -3 to Day -1 and to record caloric intake and composition in oral and enteral feeds, and PN during that time. The collected stool will be submitted to the Boston Children’s clinical laboratory for total fat quantification. To evaluate the degree of fat malabsorption, the intake data and stool fat quantification will be used to measure the baseline CFA, as previously described [15].

Treatment will be administered on study Day 1, followed by daily use of the RELiZORB enzyme cartridge whenever EN is administered, for a total of 90 days. During the treatment period, all EN will be administered through the RELiZORB enzyme cartridge. Nutritional intake (24-hour enteral dietary and PN volume intake), stool consistency/amount/frequency (if applicable) or ostomy (if applicable) output, study device use, and incidence of symptom changes will be recorded daily, in an electronic or paper diary, by the subject’s parent or guardian. Access to the nutrition/food tracking phone application along with a daily phone call from a study coordinator will be used to ensure accurate recording and completion of these tasks.

**Fig 2:**
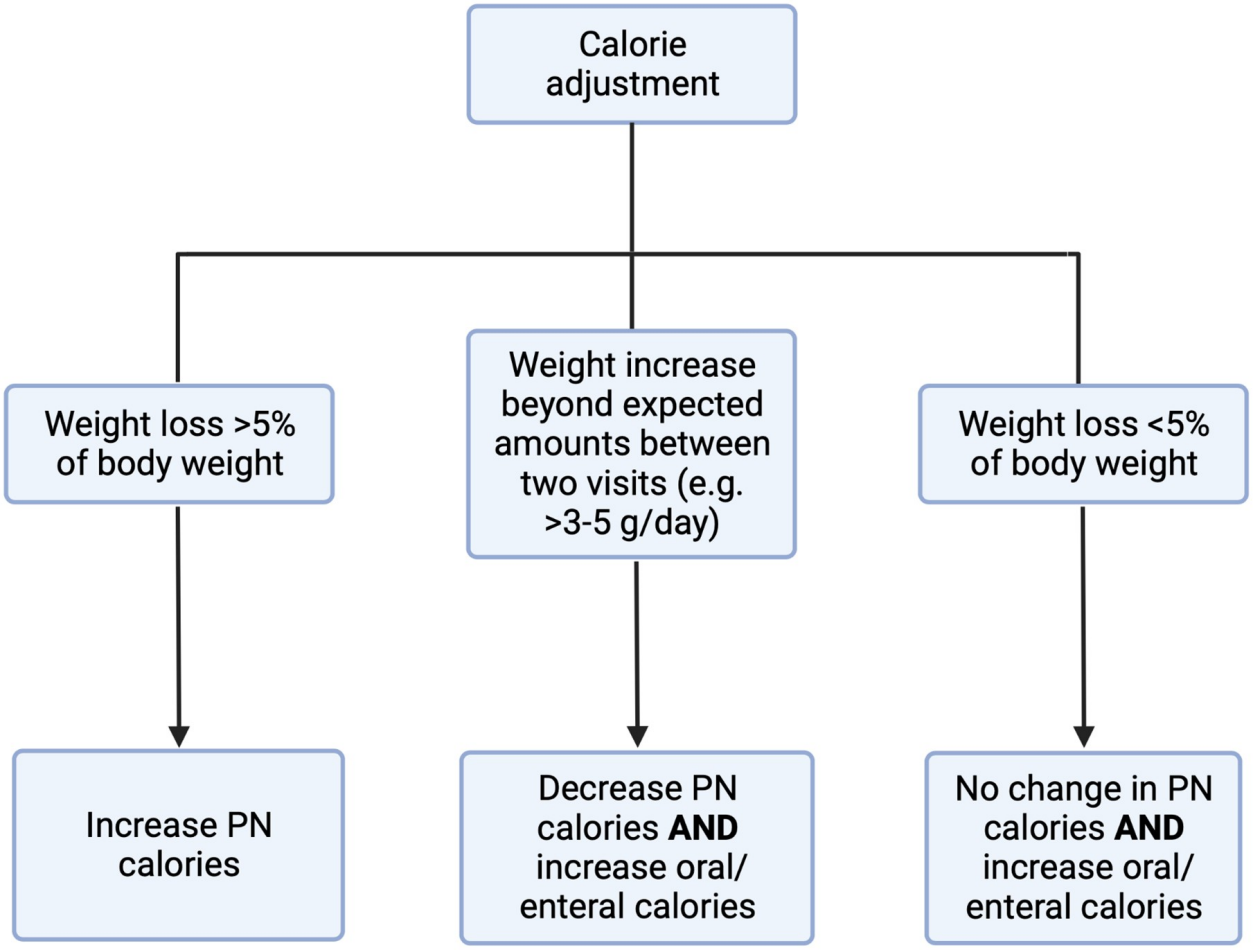
Nutritional support adjustments based on weight changes. Additional considerations include other growth parameters, laboratory evaluations (if available), overall clinical status, intake/output ratio, and enteral tolerance. Figure created with Biorender.com.

Subjects will return to the clinic on Days 7, 14, 28, 60, and 90 of the treatment period (Fig 1). The final clinic visit will be preceded by a repeat 72-hour stool collection and measurement of CFA to determine the change (if any) in intestinal fat absorption. During the clinic visits, staff will review the information in the subject’s diary, review compliance with the use of the study device and discuss relevant observations with the subject during the visit. Upon review, information contained in the diary will be entered in the electronic database. In addition to a daily study coordinator phone call, the study staff will have weekly telephone contact with families during interim weeks after the day 14 visit. Prior to these visits, subjects will be weighed at home by their parent or guardian using a standardized weight scale. At the in-person clinic and weekly telephone visits, study staff will monitor safety, diaries, weight data, stool composition (Bristol scale) and output, changes in caloric intake, changes in urine output, and will make adjustments to PN and enteral feeding accordingly (Fig 2). Adjustments will be based on the subject’s nutritional needs, weekly weights, height, hydration status and investigator’s medical judgement. If necessary, unscheduled visits can be arranged in place of the telephone contacts.

At each clinic visit, vital signs, physical examination, clinical evaluations, AEs, concomitant medications, medical/surgical procedures, and blood tests to determine the levels of liver enzymes will also be performed. Blood samples will be collected to analyze lipid profiles. All AEs post-baseline that occur will be recorded. Participants will meet with a study dietitian during each visit to assess nutritional needs and address issues or concerns.

#### Subject discontinuation

Subjects may voluntarily withdraw consent from the trial at any time. The investigators will provide a written explanation of the reason for discontinuation in a source document which will be captured and summarized in the data management system. A subject may also be removed from the study in the event of an AE, which in the judgement of the investigator, investigational product manufacturer, or the DSMB presents an unacceptable consequence or risk to the subject. Subjects may also be removed if, in the judgement of the investigator, an intercurrent illness or complication occurs that prevents the continued adherence to the protocol requirements. Noncompliance to the protocol requirements and/or refusal of investigational product administration are reasons for involuntary subject withdrawal. Reasonable efforts will be made to monitor such subjects for AEs and to complete follow-up assessments after discontinuation.

#### Adherence and related strategies

Subjects and their families will receive initial training and education on the use of RELiZORB with their enteral feeds during the screening (Day -3) and Day 1 visits. Educational pamphlets and printed instructions will complement these educational sessions. The daily coordinator phone call is incorporated into the protocol to monitor for continued adherence regarding the use of the device with each enteral feed and promote participant retention/follow-up. Any issues noted during these calls will be addressed promptly. Subjects will be considered to have complied with study treatment if ≥80% of the planned EN was administered using the RELiZORB device. The frequency and percentage of subjects who were compliant will be presented.

#### Concomitant care

Clinical care will be continued as per the discretion of the Center for Advanced Intestinal Rehabilitation (CAIR) program at Boston Children’s Hospital. Concomitant medications will be logged and tracked during each study visit in the appropriate case reporting form (CRF). As the lipase enzyme is covalently bound to beads in the device, it remains in the cartridge and is not ingested. Therefore, there is no potential for drug interactions with other medications the participant may be taking. In the event of a hospitalization, subjects will be instructed to bring their consent forms and study devices to avoid interruption in treatment. The research team will work with the clinical teams in such an event to ensure continued protocol adherence.

#### Post-trial care

Families will have the option to work with the CAIR program coordinators at our institution on obtaining insurance approval for long-term coverage of RELiZORB following the end of the study. In the event of injury harm directly results from taking part of this research, we will ensure that the required care is provided. Participants are not eligible for compensation in the event of an injury, and this is mentioned in the informed consent form.

#### Outcomes

The primary outcome of this study is the change from baseline in PN calories, assessed weekly throughout the study. The secondary outcome is the change from baseline in body weight (weight-for-age adjusted Z-score), also assessed weekly throughout the study.

Safety outcomes include the following:

1. The incidence of adverse events, grade 2 or higher. All AEs will be reported by study participants including changes in stool consistency (Bristol stool scale)/amount/frequency (if applicable), increased ostomy output (if applicable, defined as >2.5mL/kg/hour), the need to decrease enteral feeds, changes in urine color assessed by the Urine Dehydration Color scale, and discontinuation from the study treatment, assessed over the entire study.
2. The incidence of abnormalities in vital signs (blood pressure, pulse rate, respiratory rate, temperature) assessed at baseline, Days 7, 14, 28, 60, and 90.
3. The incidence of abnormalities in hematology and biochemistry parameters. based on hospital reference ranges, assessed at baseline, Days 7, 14, 28, 60, and 90.

Several exploratory outcomes will also be examined:

1. The change from baseline in 72-hour fecal fat and CFA, assessed at baseline (Days -3 to -1) and prior to the end of the study (Days 87–89).
2. The change from baseline in plasma fatty acid composition and fat-soluble vitamins (A, D, E, and K), assessed at Day 7, 14, 28, 60, and 90.
3. Change in growth height (assessed by height-for-age Z-score) and head circumference (assessed by head-circumference-for-age Z-score) [in subjects < 36 months]) from baseline, assessed at Day 7, Day 14, Day 28, Day 60, and Day 90.
4. The change in BMI-for-age Z-score assessed at Day 7, 14, 28, 60, and 90.
5. The change from baseline in PN volume, assessed weekly.
6. The change from baseline in total enteral calories and total enteral volume, assessed weekly.
7. The change from baseline in total oral calories and total oral volume, assessed weekly.
8. The ability to wean from PN, assessed over the entire study from baseline until Day 90.

#### Participant timeline

The detailed participant timeline for this protocol can be found in Fig 1.

#### Sample size

Precision-based power calculations were determined using nQuery Advisor 7.0 (GraphPad Software DBA Statistical Solutions, San Diego, CA). The primary outcome is the change in PN calories from baseline, assessed weekly at 13 assessments (study days 7, 14, 21, 28, 35, 42, 49, 56, 63, 70, 77, 84, and 90) using a baseline-adjusted time-weighted average (AUC_7-90_). In adults receiving teduglutide intended to improve enteral absorption in PN dependent patients with intestinal failure, an aim of 20% reduction was used to assess efficacy [9]. When adapted for a pediatric intestinal failure population, a ≥10% reduction was considered a valid pharmacodynamics (PD) marker of increased intestinal absorptive capacity. Based on the design of the teduglutide studies it was felt that the data provide similar support for a ≥10% reduction at Day 90 as a predictor of pharmacodynamic effect with the use of the RELiZORB device.

A reliable estimate for the standard deviation (SD) of AUC_7-90_ for the primary outcome in this patient population is unavailable. Unpublished data for seven patients are available, but at least four of these would not meet the eligibility criteria for this study (ages 1–10; one had a STEP procedure; three have pancreatic involvement). Nevertheless, the mean ± SD reduction (AUC_7-90_) for these seven patients was 0.81±0.14, representing a 19% decrease over 90 days. Using this SD with a sample size of n = 32, a 20% observed reduction in outcome would provide a 95% confidence interval (CI) = (0.75, 0.85), ruling out a true mean population reduction smaller than 15%. Conservatively, if the SD was doubled (SD = 0.28), the 95% CI = (0.7, 0.9), excluding a true mean population reduction smaller than 10%, our target threshold. In general, for any continuous outcome, a sample size of n = 32 with two-side alpha = 0.05 will provide 80% power to detect an effect size (mean/SD) as small as 0.51, considered a medium-sized effect [16].

#### Recruitment and retention

Historical review of patients followed by the CAIR program suggests that an average of eight patients per year can be expected to meet the eligibility criteria, for a total of 32 subjects over four years. Patients who follow with the CAIR program will be screened for initial eligibility. Subjects who meet the eligibility criteria will then be approached during their regular short bowel clinic visits which typically occur every two months. If interested, the study coordinators will then schedule the initial screening and enrollment study visits. All study subjects will receive all of their care through CAIR so retention is expected to be high with minimal loss to follow up.

### Data collection, management, and analysis

#### Data collection methods

Paper CRFs will be used to capture study assessments and data. The study coordinator or other delegated study personnel will enter data from source documents into the CRFs and then into the electronic database. InForm (Oracle Health Sciences, Redwood Shores, CA), an FDA Part 11 compliant electronic data capture (EDC) system, will be used for this study. The investigator will review and sign off on appropriate CRFs prior to filing. Study documents related to consent, eligibility, unanticipated adverse device effects (UADESs), and device accountability will be reviewed by the study monitor during periodic site visits. This can include consent forms, CRFs, study logs and source documents. Copies of the CRFs used in this study are available upon reasonable request.

#### Data training and management

Training will be provided to authorized study personnel for the use of InForm. All clinical trial personnel using the EDC system must have the necessary education, training, and experience or any combination of these. The investigators will be responsible for documenting employee education, training, and previous experience that pertain to the EDC system for all site personnel.

The investigators will maintain adequate security of the EDC system, including documentation that all users have been trained on the appropriate standard operating procedure and a list of authorized users. To ensure all data entries can be tracked, all personnel responsible for data entry must obtain InForm training certificates before any data can be entered in the database. The EDC system is configured to track all user entries and edits to the data. Authorized study personnel will be assigned a unique password after receiving database training. The sponsor-investigator (MP) will ensure that the electronic data systems are validated, and that data are backed up.

#### Confidentiality and data access

The anonymity and privacy of participating subjects is of the outmost importance. Subjects will be identified by an assigned subject number on CRFs and other documents retrieved from the site or sent to the study monitor, regulatory agencies, central laboratories, or blinded reviewers. Documents that identify the subject (e.g., the signed informed consent form) will be maintained in strict confidence by the investigator, except to the extent necessary to allow auditing by the appropriate regulatory authority, or the study monitor. The investigators will keep all data confidential about the nature of the proposed investigation provided by the study monitor to the investigator (with the exception of information required by law or regulations to be disclosed to the IRB, the subject, or the appropriate regulatory authority).

Upon study completion, the investigators will retain control of the data. Even though the final dataset will lack identifiers, we believe that there remains the possibility of deductive disclosure of subjects given the limited number of enrolled subjects as well as the rarity of SBS. Therefore, we will make the data and associated documentation available to users only under a data-sharing agreement that provides for: 1) a commitment to using data only for research purposes and not to identify any individual participant; 2) a commitment to securing the data using appropriate computer technology; and 3) a commitment to destroying or returning the data after analyses are completed.

#### Statistical methods: Outcomes evaluation

Data will be summarized in tables listing the number of subjects, mean, standard deviation, standard error, 95% confidence interval (CI), median, interquartile range (IQR), minimum, and maximum for continuous data and number of subjects, frequency and percentage for categorical data. Summaries will be presented by subject and visit, as well as in aggregate when appropriate. Outcomes will be presented in their native units (i.e. as collected), in addition to transformed units when necessary (for example logarithmic). Anthropometry outcomes (weight, height, head circumference, body mass index) will be examined as Z-scores using the Centers for Disease Control growth charts. All outcomes will be explored visually using various graphing methods, including (but not limited to) box-whisker, profile, and bar plots. All statistical analyses will be performed with SAS version 9.4 or later (Cary, NC).

Continuous outcomes, which include all outcomes except the incidence of AEs and incidence of weaning from PN, will be described as indicated in the general overview above using summary measures at each visit. Incidence of ever weaning from PN will be summarized by frequency count and percentage.

Assessment of change in continuous outcomes will be made using the area under the curve between study day 7 and 90 (AUC_7-90_), adjusted for baseline (Day 1), calculated over all relevant assessment timepoints (5 and 13 post-baseline weekly assessments for measurements obtained in clinic and at home, respectively). Measurements obtained in clinic will be assessed at study days 7, 14, 28, 60 and 90, while those obtained at home will be assessed on study days 21, 35, 42, 49, 56, 63, 70, 77, 84.

Adjustment for baseline will be made by dividing the outcome value at each assessment by the baseline value, leading to interpretation of AUC_7-90_ as a mean relative percent increase or decrease in outcome depending on whether AUC_7-90_ is greater or less than unity (1), respectively. AUC_7-90_ will be calculated as a time-weighted average, given by the equation:

$$\frac{0.5∙\sum \left(t_{i+1}-t_{i}\right)\left(r_{i}+r_{i+1}\right)}{\sum \left(t_{i+1}-t_{i}\right)}$$

Where *t*_*i*_ denotes the assessment day as described above, and *r*_*i*_ = *y*_*i*_/*y*_1_ denotes the outcome at assessment day *t*_*i*_ divided by the baseline (day 1) value.

The assumption of normality will be assessed for AUC_7-90_ for each continuous outcome using quantile-quantile (Q-Q) plots and the Shapiro-Wilk test. Outcomes determined to be skewed will be normalized using an appropriate transformation (e.g. logarithm; normal scores based on ranks). AUC_7-90_ for each outcome will be summarized as described above.

#### Statistical methods: Safety analysis

Safety analysis will be performed on all subjects enrolled in the study with a follow-up safety assessment. Adverse event data will be listed individually and summarized by system organ class and preferred terms within a system organ class. The number and percentage of subjects with AEs, serious adverse events (SAEs), AEs that lead to discontinuation, study device-related AEs (determined by the investigator), and AEs that lead to death will be summarized. Only AEs grade 2 or higher will be presented. When calculating the incidence of AEs, each adverse event will be counted only once for a given subject. If the same AE occurs on multiple occasions in a subject, the occurrence with the highest severity and relationship to study device will be reported. If two or more adverse events are reported as a unit, the individual terms will be reported as separate events. AEs will also be summarized with regards to severity and relation to study drug. Additionally, the frequency of each AE within-subject will be calculated and summarized across all subjects in order to rank the commonality of each particular AE.

Vital signs, hematology, and clinical chemistry parameters from baseline to the end of the study will be presented as outlined above for continuous outcomes and reported to the Data Safety Monitoring Board (DSMB) at the regularly scheduled meetings. Incidence of changes in laboratory parameters from normal to abnormal, based on Boston Children’s Hospital reference ranges, will be identified and lab values listed by subject.

#### Statistical methods: Additional considerations

The primary population analyzed for efficacy will be the full-analysis population (often referred to as intention-to-treat), defined as all subjects enrolled in the study. This population will include all enrolled subjects regardless of protocol deviations, compliance, or number of visits completed. A per-protocol population will be used for sensitivity analysis where results will be compared to the full-analysis population. The per-protocol population will exclude subjects with substantial deviations from the protocol (as determined by the investigator prior to database lock), <80% compliance, intercurrent illness for >20% of follow-up, or insufficient study visits (as determined by the investigator prior to database lock). The safety population will include a subset of the full-analysis population who received at least one treatment with RELiZORB and who have at least one adverse event grade 2 or higher.

The default method for calculating area under the curve when either the first follow-up visit (Day 7), last follow-up visit (Day 90), or both first and last follow-up visits are missing will be to evaluate from the first available follow-up visit to the last available follow-up visit. That is, AUC_F-L_ (area under the curve from first available until last available) will be calculated with no change in outcome indicated by AUC_F-L_ = 1. Sensitivity of the area under the curve to unknown data will be investigated using a best- and worse-case scenario method, as detailed in the study protocol (S2 File).

### Monitoring

#### Study coordination and execution

The daily operations of this project will be managed by study coordinators and project managers. Study staff will also include a dedicated dietitian, pharmacist, postdoctoral research fellow, biostatistician, and research assistants. The principal and associated investigators will oversee all study operations. Weekly team meetings will discuss study progress, enrollment, any necessary protocol amendments, AE status and classification, and address any issues as they occur. Details regarding study documentation are available in the complete study protocol (S2 File).

#### Data monitoring

The DSMB will be a board comprised of three clinicians who are subject experts, and one biostatistician not participating in this study–all independent of the study investigators and sponsors. One of the clinicians will be appointed as chairman of the DSMB. The DSMB is responsible for safeguarding the interests of study participants, assessing the safety and efficacy of study procedures, and for monitoring the overall conduct of the study. The first meeting will occur prior to the first participant’s enrollment. The DSMB will then convene once the first participant has enrolled into the study, followed by every six months to review limited cohort safety data with additional meetings scheduled as needed. Specific details about the DSMB composition, responsibilities, organization and meeting frequency can be found in the DSMB Charter (S4 File).

No interim analysis or early stopping rules are planned given the sample size and protocol design. However, the DSMB will have the right to recommend stopping the trial early if safety concerns arise. Additionally, an independent site monitor will perform periodic visits to review consent, eligibility and unanticipated adverse device effects (UADEs) for all enrolled subjects, as well as device accountability documents.

#### Adverse events and related procedures

Any adverse events that occur after the use of RELiZORB enzyme cartridge will be recorded and tracked in a dedicated AE log. AEs will be defined, graded, and classified according to the *Common Terminology Criteria for Adverse Events*, *v*.*5*.*0* [17]. The subject will receive appropriate treatment and medical supervision for any AE that occurs. Subjects with anaphylactic or allergic reactions will not continue to receive the study device. All AEs judged to be clinically significant, including clinically significant laboratory abnormalities, will be followed until resolution. All AEs will be summarized in the annual report or more frequently if requested by the regulatory agency.

For the purposes of this trial, any AE that is results in death, is life-threatening, requires hospitalization or prolongation of an existing hospitalization, results in a persistent or significant incapacity or substantial disruption of the ability to conduct normal life operations, or other medically important events will be considered a SAE. Any SAE occurring after use of the study device must be reported to the IRB and the Data and Safety Monitoring Board (DSMB) by phone or in person within 72 hours of the time the investigator becomes aware of the SAE, or within 24 hours if the event is fatal or life threatening.

#### Auditing

This study will be monitored through an independent monitoring program at our institution. This monitoring program follows established auditing procedures as outlined in the FDA Guidelines for the Monitoring of Clinical Investigations, as well as the International Council for Harmonization of Technical Requirements for Pharmaceuticals for Human Use (ICH) Good Clinical Practices. After enrollment of the first subject, an in-person monitoring visit will occur every three months for the duration of the study. During these visits, patient records specifically related to consent, eligibility, device accountability, and unanticipated adverse device effects will be reviewed for accuracy and completeness. Based on these reviews, reports will document the findings, if any, and any actions that need to be taken to correct any issues. The study team will be responsible for reviewing each report and ensuring that the proper follow-up actions occur as deemed necessary. A final monitoring visit will occur at the end of the study to ensure that all study-related documentation is present and complete.

### Trial status

The finalized protocol version is v3.0. The protocol has been reviewed and approved by the Boston Children’s Hospital Institutional Review Board (Initial approval date: May 29^th^ 2018, #IRB-P00028297). Recruitment and enrollment at Boston Children’s Hospital began in June, 2022. Enrollment is estimated to be completed by July, 2026.

## Discussion

### Protocol amendments and communication

Substantive changes in the protocol include changes that affect the safety of subjects or changes that alter the scope of the investigation, the scientific quality of the study, the experimental design, dosages, assessment variable(s), the number of subjects treated, or the subject-selection criteria. Such changes will be prepared as a protocol amendment. A protocol amendment must receive IRB approval before implementation. In parallel with the IRB approval process, the protocol amendment will be submitted to the appropriate regulatory authority as an amendment to the regulatory submission under which the study is being conducted. If a protocol amendment requires changes in the informed consent form, the revised informed consent form prepared by the investigator must also be approved by the IRB before implementation.

Departures from the protocol are allowed only in situations that eliminate an immediate risk to a subject and that are deemed crucial for the safety and well-being of that subject. The investigator or the attending physician also will contact the IRB as soon as possible in the case of such a departure. These departures do not require preapproval by the IRB; however, the IRB must be notified in writing as soon as possible after the departure has been made. In addition, the investigator will document in the subject’s CRF the reasons for the protocol deviation and the ensuing events.

This study is registered with ClinicalTrials.gov (NCT03530852), and the results will be submitted to ClinicalTrials.gov as outlined in the NIH Policy on the Dissemination of NIH-Funded Clinical Trial Information. The entry will be maintained by study team members to ensure that the most up-to-date information about the trial is communicated to the public.

### Dissemination plan

The results of this research will be disseminated in several ways. Submissions to conference proceedings at the regional and national level will serve to disseminate these findings to academic and healthcare professionals. The results of this work will also be submitted in the form of a manuscript to an appropriate scientific journal. Finally, our findings may be included in data submissions or fillings with regulatory agencies as deemed necessary. As all study participants will be patients of our institution, laboratory results obtained during the study, will be made available in the subject’s medical record and communicated in real time to the subject’s and their families.

### Limitations

This study is designed as an open-label, pre- and post-, single center clinical trial where each subject serves as their own control in comparison to an established baseline. Prior to enrollment, subjects need to demonstrate stability on their EN and PN regimens with no significant changes within one month of screening. Due to the rarity of SBS, two parallel arms (e.g., as in a randomized control trial) are not feasible in the design of this single-center study. In general, the small participant numbers, often seen in rare diseases, make conventional parallel-arm studies prone to bias because of chance imbalance between groups [18, 19]. An important consideration specific to SBS is also the extensive heterogeneity of the patient population [2], which further limits a two-arm approach given the potential for additional confounding. We considered use of a crossover study design, but this was also deemed not feasible given the potential for disease-modifying treatment to occur with bowel adaptation while receiving the RELiZORB cartridge, which would preclude a ‘wash-out’ period, and the ethical concern of removing a potential disease-modifying therapy that may reduce highly morbid complications such as IFALD and CLABSIs through reduction of parenteral nutrition requirement.

An important limitation of our protocol is the small sample size which may limit the power to detect changes in several of the study’s outcomes. The *a priori* power calculation was based upon detection of a clinically relevant decrease (10%) in PN requirements from baseline, but the sample size may be underpowered for the exploratory outcomes. Furthermore, the open label study design can be a limitation due to the potential for bias in the study outcomes. To minimize and safeguard against this, we have incorporated a previously validated advancement protocol (Fig 2) that will be strictly followed, per protocol design. In addition, all decisions regarding PN/EN changes will be made by the primary treating gastroenterologist and dietitian at the CAIR program, independent of study investigators.

### Role of sponsor

This is an investigator-initiated study, and the sponsor is the principal investigator who oversees all aspects of the study design, execution, and analysis (MP). Decisions regarding study design, data collection, management, analysis, and interpretation, writing of the report, and the decision to submit the report for publication were made independent of the study funders who do not have direct authority over these activities.

## Supporting information

S1 File SPIRIT checklist. (DOCX)

S2 File Study protocol. (DOCX)

S3 File Informed consent. (PDF)

S4 File Data Safety Monitoring Board (DSMB) charter. (DOCX)
