# Supplementary material for: Fat digestion using RELiZORB in children with short bowel syndrome who are dependent on parenteral nutrition: Protocol for a 90-day, phase 3, open labeled study
Source: PLoS One. 2023 Mar 1;18(3):e0282248. doi: 10.1371/journal.pone.0282248 (PMC9977023; doi:10.1371/journal.pone.0282248)
Supplement: S2 File — (DOCX) [file pone.0282248.s002.docx]

|  | **RELiZORB** | |
| --- | --- | --- |
| **CLINICAL PROTOCOL** |  |  |
|  |  | |

**A 90 day, Phase 3, Open Labeled Exploratory Study of RELiZORB to Evaluate Safety, Tolerability, and Nutrient Absorption in Children with Short Bowel Syndrome who are Dependent on Parenteral Nutrition**

| **July 5, 2022** | |
| --- | --- |
|  |  |

**Version 3**

This study is to be performed in accordance with Good Clinical Practice, the ethical principles that have their origin in the Declaration of Helsinki, Title 21 of the Code of Federal Regulations, Parts 50 (Protection of Human Subjects), and 56 (Institutional Review Boards), and 812 (Investigational Device Exemption Application), and International Council on Harmonization E6 (Guideline for Good Clinical Practice).

| Sponsor-Investigator: | Mark Puder, M.D., Ph.D. |  |
| --- | --- | --- |
|  | Professor of Surgery, Harvard Medical School |  |
|  | Phone: 617-355-1838 |  |
|  | Fax: 617-730-0477 |  |
|  | E-mail: Mark.Puder@childrens.harvard.edu |  |
|  |  |  |
| Sub-investigators: | Kathleen Gura, PharmD Assistant Professor of Pediatrics |  |
|  | Clinical Pharmacist GI/Nutrition Manager, Clinical Research |  |
|  | Assistant Professor of Pediatrics, Harvard Medical School |  |
|  | Phone: 617-355-2336 |  |
|  | Fax: 617-730-0601 |  |
|  | E-mail: Kathleen.Gura@childrens.harvard.edu |  |
|  |  |  |
|  | Alexandra Carey, M.D. |  |
|  | Director, Home Parenteral Nutrition Program |  |
|  | Division of Gastroenterology, Hepatology and Nutrition |  |
|  | Phone: 617-355-7964 |  |
|  | E-mail: Alexandra.Carey@childrens.harvard.edu |  |
|  |  |  |
|  | Steven David Freedman, M.D., Ph.D. |  |
|  | Professor of Medicine |  |
|  | Beth Israel Deaconess Medical Center |  |
|  | Dana 501 - East Campus |  |
|  | 330 Brookline Ave |  |
|  | Phone: 617-667-5576 |  |
|  | E-mail: sfreedma@bidmc.harvard.edu |  |
|  |  |  |
|  | Camilia Rivera Martin, M.D, MS |  |
|  | Professor of Pediatrics |  |
|  | 1283 York Avenue, 15^th^ Floor |  |
|  | New York, NY 10065 |  |
|  | Phone: 646-697-6428 |  |
|  | E-mail: cam4024@med.cornell.edu |  |
|  |  |  |
|  |  |  |
| Research facilities: | Boston Children’s Hospital |  |
|  | 300 Longwood Ave, Boston, MA 02115 |  |
|  |  |  |
| Institutional Review Board: | Boston Children’s Hospital Institutional Review Board |  |
|  |  |  |
|  |  | |
|  |  | |
|  |  | |
|  |  | |

STUDY SYNOPSIS

| **Name of finished product:** RELiZORB | |
| --- | --- |
| **Name(s) of active ingredient(s):** The active ingredient in RELiZORB is the digestive enzyme lipase, attached to polymeric carriers together called iLipase®. | |
| **Title of study:** A 90 day, Phase 3 Open Labeled Exploratory Study of RELiZORB to Evaluate Safety, Tolerability, and Nutrient Absorption in Children with Short Bowel Syndrome who are Dependent on Parenteral Nutrition. | |
| **Investigator(s):** Mark Puder, MD, PhD; Kathleen Gura, PharmD, BCNSP; Alexandra Carey, MD; Steven Freedman MD, PhD; Camilia Martin MD, MS | |
| **Number of sites:** Single site study (Boston Children’s Hospital) | |
| **Study period:** Approximately 90 days | **Phase of development:** Phase 3 |
| **Objectives**  The objectives of this study are to determine the safety, tolerability, and nutrient absorption of enteral nutrition administered through the RELiZORB enzyme cartridge for a total of 90 days. Measurements will be assessed at baseline and on study Day 7, 14, 28, 60, 90 for in-clinic assessments and weekly for at-home assessments in pediatric subjects with short bowel syndrome (SBS), aged 2 years – 18 years, who are dependent on parenteral nutrition (PN).  Primary objective:   - To evaluate the effect of the RELiZORB enzyme cartridge when used with enteral nutrition daily for 90 days on the change from baseline in PN calories.   Secondary objective:   - To evaluate the effect of the RELiZORB enzyme cartridge when used with enteral nutrition daily for 90 days on the change from baseline in body weight (weight-for-age z-score).   Safety objectives:  To evaluate the safety and tolerability of the RELiZORB enzyme cartridge when used with enteral nutrition daily for 90 days, by examining:   - The incidence of grade 2 or higher adverse events. All adverse events will be collected by participants, including diarrhea, changes in stool consistency (Bristol scale)/ amount/frequency (if applicable), increased ostomy output (if applicable, defined as >2.5 mL/kg/hour), the need to decrease enteral feeds, changes in urine color assessed by Dehydration Color scale, and discontinuation from the study treatment. - The incidence of abnormalities in vital signs (blood pressure, pulse rate, respiratory rate, temperature). - The incidence of abnormalities in hematology and biochemistry parameters based on Boston Children’s Hospital reference ranges. Clinical lab evaluations will include sodium, potassium, chloride, CO2, glucose, blood urea nitrogen (BUN), creatinine, calcium, phosphorus, magnesium, alkaline phosphatase, albumin, pre-albumin, total protein, total and direct bilirubin, triglycerides, cholesterol, high-density lipoprotein (HDL), very low-density lipoprotein (VLDL), low-density lipoprotein (LDL), C-reactive protein (CRP), AST, ALT, GGT and fatty acid profile.   Exploratory objectives:   - To evaluate the effect of the RELiZORB enzyme cartridge when used with enteral nutrition daily for 90 days on the change from baseline in nutrient absorption as measured by 72-hour total fecal fat, coefficient of fat absorption, and fecal fatty acid composition. - To evaluate the effect of the RELiZORB enzyme cartridge when used with enteral nutrition daily for 90 days on the change from baseline in plasma fatty acid composition. - To evaluate the effect of the RELiZORB enzyme cartridge when used with enteral nutrition on growth as measured by height and head circumference (in children <36 months of age) using adjusted-for-age Z-score. - To evaluate the effect of the RELiZORB enzyme cartridge when used with enteral nutrition daily for 90 days on the change from baseline in BMI z-score. - To evaluate the effect of the RELiZORB enzyme cartridge when used with enteral nutrition daily for 90 days on the change from baseline in PN volume. - To evaluate the effect of the RELiZORB enzyme cartridge when used with enteral nutrition daily for 90 days on the change from baseline in total enteral calories and total enteral volume. - To evaluate the effect of the RELiZORB enzyme cartridge when used with enteral nutrition daily for 90 days on the change from baseline in total oral calories and total oral volume. - To evaluate the effect of the RELiZORB enzyme cartridge when used with enteral nutrition daily for 90 days on the ability to wean from PN. | |
| **Methodology:**  This study is a phase 3 open labeled uncontrolled single center clinical trial in pediatric subjects with SBS, aged 2 years – 18 years who have been dependent on PN for at least 6 months. Treatment will be the use of the RELiZORB enzyme cartridge with the administration of enteral nutrition daily for a total of 90 days. This study will be open labeled; treatment allocation will be known by the subjects, Principal Investigator and his medical staff. | |
| This clinical trial includes an initial screening visit (Day -3) in which subjects will be screened for eligibility, including baseline clinical and physical assessments. At screening the investigator or designee will review the subject’s medical history to determine eligibility, explain the study to the subject and the subject’s parent or legal guardian, and if eligible, obtain informed consent. If consent is given and the subject is deemed eligible based on the results of the screening visit, they will be enrolled in the study and pre-baseline assessments will be performed, which will include physical examination, nutritional assessment, vital signs, 72-hour fecal stool collection, and clinical evaluations.  Treatment will be administered on study Day 1, followed by daily use of the RELiZORB enzyme cartridge whenever enteral nutrition is administered, for a total of 90 days. During the treatment period, all enteral nutrition will be administered through the RELiZORB enzyme cartridge. Nutritional intake (24-hour enteral dietary and PN volume intake), stool consistency/amount/frequency (if applicable) or ostomy (if applicable) output, study device use, and incidence of symptom changes will be recorded daily electronically or on paper by the subject’s parent or guardian. Throughout the duration of the study, parents or guardians will also be contacted daily by a study coordinator and/or dietician to assess nutritional record and identify any other potential problems. Clinic visits will occur on Days 7, 14, 28, 60, and 90 (range ± 3 days) of the treatment period. Assessments for the primary endpoint and safety will be collected during these visits and through a weekly phone call with at-home weight measurement using a standardized scale. Advancement of oral or enteral feedings and weaning from parenteral feeding will be determined by the investigator during the weekly phone calls and scheduled clinic visits based on the subject’s clinical status, which will include assessment of weight, growth and hydration status. | |
| **Number of subjects (planned):** A total of 32 pediatric subjects with SBS, aged 2 years -18 years, are expected to be enrolled in the study over a period of 48 months. | |
| **Inclusion criteria:**   1. Male or female patients, ages 2 years to 18 years, inclusive. 2. Diagnosed with SBS, as determined by medical history and PN dependence (i.e. need for PN for >60 days after intestinal resection or a bowel length <25% of expected). 3. Congenital or acquired gastrointestinal disease requiring surgical intervention that has occurred at least 3 months prior to screening. 4. Patient is on parenteral lipid and at least 30% of daily caloric and fluid intake has been provided by PN for a least 6 months prior to screening 5. Stable PN nutrition requirement, determined by less than 5% reduction in PN nutrition calories for at least 1 month prior to screening, or at the discretion of the investigator. 6. Screening direct bilirubin that is in the normal range for age and is not determined to be clinically significant by the investigator. 7. Subject has an existing feeding tube, is receiving enteral nutrition via a pump at a rate>10ml/hr but <120ml/hr, and is able to tolerate at least 10 ml/kg/day enteral nutrition. 8. Stable enteral nutrition requirement with no change in formula composition or rate for at least 1 month prior to screening. 9. The parent or legal guardian of the patient is able to read, understand, and is willing to provide informed consent (and assent, if applicable). 10. The patient (if assent is applicable) or parent or legal guardian of the patient is able to understand the requirements of the study and is willing to bring the patient to all clinic visits and complete all study related procedures (as determined by the investigator). 11. A parent or legal guardian is willing to provide written authorization for the use and disclosure of protected health information. | |
| **Exclusion criteria:**   1. Other causes of chronic liver disease other than SBS (i.e., hepatitis C, cystic fibrosis, biliary atresia, alpha 1 anti-trypsin deficiency, and Alagille syndrome). 2. The patient has had a bowel lengthening procedure, including but not limited to, a STEP procedure. 3. Any serum triglyceride concentration >400 mg/dL at screening. 4. Pancreatic insufficiency as defined as the use of pancreatic enzymes within 30 days prior to screening. 5. Evidence of untreated intestinal obstruction or active stenosis, as determined by the investigator. 6. Unstable absorption due to cystic fibrosis or known DNA abnormalities (i.e., familial adenomatous polyposis, Fanconi syndrome) as determined by the investigator. 7. History of microvillus inclusion disease, as determined by medical history. 8. Severe known dysmotility syndrome (i.e., pseudo-obstruction, gastroschisis-related motility disorders), as determined by the investigator. 9. Initiation of teduglutide or other GLP-2 analogues within 6 months of screening 10. Use of growth hormone, or supplemental glutamine within 3 months prior to screening. 11. Use of cisapride within 30 days prior to screening. 12. Active clinically significant pancreatic or biliary disease, as determined by the investigator. 13. Patients are receiving formulas that are not compatible with the RELiZORB cartridge (example, insoluble fiber-containing formulas) 14. Determined by the investigator to be unsuitable for participation for any reason. | |
| **Test product, dose, and mode of administration:**  RELiZORB is a digestive enzyme cartridge designed to mimic the function of pancreatic lipase. RELiZORB is designed for use by patients receiving enteral tube feeding who have trouble breaking down and absorbing fats. RELiZORB is developed using Alcresta’s proprietary enzyme immobilization technology. The active ingredient in RELiZORB is the digestive enzyme lipase, attached to polymeric carriers together called iLipase®.  As the enteral tube feeding formula passes through RELiZORB, it makes contact with the iLipase and the fat in the formula is broken down to its absorbable form (fatty acids and monoglycerides) prior to ingestion. The iLipase remains in the cartridge and does not become part of what is ingested. RELiZORB has been shown to break down 90 percent of fats in most enteral feeding tube formulas, including the most difficult to break down long-chain polyunsaturated fatty acids, such as docosahexaenoic acid (DHA), eicosapentaenoic acid (EPA) and arachidonic acid (ARA), which are critical for growth and development.  RELiZORB is a single-use, point-of-care digestive enzyme cartridge that connects in-line with existing enteral feeding pump tubing sets and patient extension sets or enteral feeding tubes. RELiZORB is comprised of a clear cylindrical, plastic cartridge with a single inlet connection port and a single purple outlet connection port. The inlet and outlet ports of RELiZORB are intended to connect in-line with enteral feeding pump tubing sets and patient extension sets or enteral feeding sets. Inside the cartridge, there are small white beads which are compromised of the digestive enzyme, lipase that is covalently bound to the small white beads. The lipase-bead complex is retained within the cartridge during use by filters on both ends of the cartridge. The fat in enteral formulas is hydrolyzed when it comes into contact with iLipase as the formula passes through the cartridge. | |
| **Duration of treatment:** The total duration of study participation will be 90 days during which the patient will receive all enteral nutrition after it has passed through the RELiZORB enzyme cartridge. Screening will be performed prior to the start of the study. The first use of the RELiZORB enzyme cartridge with enteral nutrition will occur on study Day 1 and all subsequent enteral nutrition will continue to be administered using a RELiZORB device for a total of 90 days. | |
| **Criteria for evaluation:**  Treatment will be initiated on study Day 1.  Primary outcome:   - The change from baseline in PN calories, assessed weekly throughout the study.   Secondary outcome:   - The change from baseline in body weight (weight-for-age z-score), assessed weekly throughout the study.   Safety outcomes:   - The incidence of adverse events, grade 2 or higher. All adverse events will be reported by study participants including changes in stool consistency (Bristol scale)/amount/frequency (if applicable), increased ostomy output (if applicable, defined as >2.5 mL/kg/hour), the need to decrease enteral feeds, changes in urine color assessed by Dehydration Color scale and discontinuation from the study treatment, assessed over the entire study from baseline until Day 90. - The incidence of abnormalities in vital signs (blood pressure, pulse rate, respiratory rate, temperature) assessed at baseline, Day 7, 14, 28, 60, and 90. - The incidence of abnormalities in hematology and biochemistry parameters, based on Boston Children’s Hospital reference ranges, assessed at baseline, Day 7, 14, 28, 60, and 90.   Exploratory outcomes:   - The change from baseline in 72-hour fecal fat and coefficient of fat absorption, assessed at baseline (Days -3 to -1) and prior to the end of the study (Days 87-89). - The change from baseline in plasma fatty acid composition and fat-soluble vitamins (A, D, E, and K), assessed at Day 7, 14, 28, 60, and 90. - Change in growth height (assessed by height-for-age Z-score) and head circumference (assessed by head-circumference-for-age z-score) [in subjects < 36 months]) from baseline, assessed at Day 7, Day 14, Day 28, Day 60, and Day 90 - The change in BMI z-score assessed at Day 7, 14, 28, 60, and 90. - The change from baseline in PN volume, assessed weekly. - The change from baseline in total enteral calories and total enteral volume, assessed weekly. - The change from baseline in total oral calories and total oral volume, assessed weekly. - The ability to wean from PN, assessed over the entire study from baseline until Day 90. | |
| **Experimental procedures:**  **Screening Period (Day -3)**  The following evaluations will be performed to assess the subject’s eligibility for the study:   - Complete medical history - Vital signs   - Temperature, blood pressure, pulse rate, and respiratory rate - Clinical lab evaluations   - Chemistry (sodium, potassium, chloride, CO2, glucose, blood urea nitrogen (BUN), creatinine, calcium, phosphorus, magnesium, alkaline phosphatase, albumin, pre-albumin, total protein, total and direct bilirubin, triglycerides)   - Lipid profile (i.e. cholesterol, high-density lipoprotein (HDL), very low-density lipoprotein (VLDL), low-density lipoprotein (LDL))   - Serum C-reactive protein (CRP), fatty acid profile | |
| - Nutritional intake   - Oral and enteral intake and PN volume intake   - Stool consistency (Bristol scale)/amount/volume (if applicable)   - Ostomy (if applicable) output | |
|  | |
| - Liver enzymes   - Serum alanine aminotransferase (ALT)   - Serum aspartate aminotransferase (AST)   - Serum gamma-glutamyl transpeptidase (GGT)   Results from screening must not show any clinically significant abnormalities in order for the subject to be enrolled and progress to the treatment phase of the trial. | |
| **Enrollment (Day -3 to -1) and First Day of Treatment (Day 1)**  Assessments of safety and clinical status will include:   - Vital signs (temperature, blood pressure, pulse rate, and respiratory rate) - Physical examination - Height or length, head circumference (for subjects < 36 months), and weight. - NOTE: physical examinations performed during the screening period will be used for the baseline assessment when available.   Assessments of clinical endpoints will include:   - 72-hour fecal collection for total fat and fatty acid analysis - Lipid profile (i.e., cholesterol, high-density lipoprotein (HDL), very low-density lipoprotein (VLDL), low-density lipoprotein (LDL) - Fat-soluble vitamins (A, D, E, and K) - Liver Tests (AST, ALT, GGT) - Total plasma fatty acids - total saturated, total monosaturated, total polyunsaturated, total Omega 3, total Omega 6, total fatty acids - a-linolenic Acid, linoleic acid, oleic acid, EPA, arachidonic acid, mead acid, DHA, triene:tetraene ratio - Nutritional intake - Daily caloric intake of enteral fat and PO as well as PN volume will be documented | |
| The investigator will assess the subject’s nutritional intake, weight, height, and hydration status to determine if, based on their medical judgment, changes to the subject’s nutritional treatment should be made.  The subject’s parent or guardian will be given access to an electronic diary(or given a paper diary if no electronic capability) and instructed to record daily any changes in symptoms (e.g., abdominal pain, increases in stool output above baseline, stool consistency through Bristol scale), stool amount/frequency (if applicable), ostomy (if applicable) output and use of the study device. The subject’s parent or guardian will be instructed on how to administer enteral nutrition using the RELiZORB enzyme cartridge and will be provided with RELiZORB enzyme cartridges to take home. The subject’s parent or guardian will also receive education from a registered dietician on recording caloric intake and composition in oral and enteral feeds, and TPN. As part of this education, the subject’s parent or guardian will receive access to a nutrition/food tracking phone application (Cronometer) to assist them in this task throughout the study.  The subject’s parent or guardian will then be asked to collect all stool for 72-hours over study Day -3 to Day -1 and to record caloric intake and composition in oral and enteral feeds, and TPN during that time. This will be used to determine the baseline coefficient of fat absorption.  The first enteral feeding with the RELiZORB enzyme cartridge will occur under the observation of a member of the research team (Day 1). The parent or guardian will supply and prepare the enteral formula. The feeding regimen, including type of formula, will not be changed for research purposes. Subjects will remain on their current regimen. Additionally, only standard (non-blenderized) commercial formulas will be permitted to be used with the RELiZORB enzyme cartridge. Amino-acid based formulas that are compatible with the RELiZORB enzyme cartridge will be permitted based on the inclusion criterion requiring formulas be compatible with the device prior to study enrollment.  Any Adverse Events (AEs) that occur after the use of RELiZORB enzyme cartridge will be recorded according to the procedures described below. Any significant clinical finding prior to use of the study device will be included in the subject’s medical history.  **Treatment Period (Day 1 to 90)**  During the treatment period (Day 1-90) all enteral nutrition via pump (tube feeds) will be administered through a RELiZORB enzyme cartridge. RELiZORB is a single-use product and a new device will be used with each new enteral feeding period. Re-use may result in contamination of the product. If re-used, RELiZORB may not effectively hydrolyze fats.  The subject’s parent or guardian will record daily the use of the device, the subject’s nutritional intake (24 hour oral and enteral intake and PN volume) and any changes, stool consistency (Bristol scale)/amount/frequency (if applicable), ostomy (if applicable) output, ,any changes in symptoms (e.g., abdominal pain, increases in stool output above baseline), and urine color using an a nutrition phone application and electronic diary (or using a paper diary if no electronic capability) . Access to a nutrition/food tracking phone application along with a daily phone call from a study coordinator will be used to ensure accurate recording and completion of these tasks. In addition, if the subject experiences any unexpected physical or medical condition, or an in-hospital admission, the subject’s parent or guardian will be directed to call the clinic immediately and report it to the study staff.  Subjects will return to the clinic on Day 7, Day 14, Day 28, Day 60, and Day 90 of the treatment period. The final clinic visit will be preceded by a repeat 72-hour stool collection for fat analysis. During the clinic visits, staff will review the information in the subject’s diary, review compliance with the use of the study device and discuss relevant observations with the subject during the visit. Information contained in the diary will be entered in the electronic database in a timely manner. In addition to a daily study coordinator phone call, the study staff will have weekly telephone contact with families during interim weeks after the day 14 visit. Study staff will monitor safety, diaries, weekly weights, stool composition (Bristol scale) and output, changes in caloric intake, changes in urine output, and will make adjustments to PN and enteral feeding accordingly. Adjustments will be based on the subject’s nutritional needs, weight, height, hydration status and investigator’s medical judgement. If necessary, unscheduled visits can be arranged in place of the telephone contacts.  At each clinic visit, vital signs, physical examination, clinical evaluations, adverse events, concomitant medications, medical/surgical procedures, and blood tests to determine the levels of liver enzymes will be performed. Blood samples will also be collected to analyze lipid profiles. All AEs post-baseline that occur will be recorded. Participants will meet with a study dietician during each clinic visit to assess nutritional needs and address issues or concerns. | |
| **Statistical methods**  Data will be summarized in tables listing the number of subjects, mean, standard deviation, standard error, 95% confidence interval (CI), median, interquartile range (IQR), minimum, and maximum for continuous data and number of subjects, frequency and percentage for categorical data. Summaries will be presented by subject and visit, as well as in aggregate when appropriate. All statistical analyses will be performed with SAS version 9.4 or later (Cary NC). | |
| Assessment of change in continuous outcomes will be made using the area under the curve, adjusted for baseline, calculated over study day 7 to 90 (AUC_7-90_). Adjustment for baseline will be made by dividing the outcome value at each visit by the baseline value, leading to interpretation of AUC_7-90_ as a mean relative percent increase or decrease in outcome depending on whether AUC_7-90_ is greater or less than unity (1), respectively. The assumption of normality will be assessed for AUC_7-90_ for each continuous outcome using quantile-quantile (Q-Q) plots and the Shapiro-Wilk test. Outcomes determined to be skewed will be normalized using an appropriate transformation (e.g. logarithm; normal scores based on ranks).  Adverse event data will be listed individually and summarized by system organ class and preferred terms within a system organ class. The adverse events will be recorded by the study nurse and/ or study physician and classified using the *Common Terminology Criteria for Adverse Events Version 5.0.* The number and percentage of subjects with grade 2 or higher adverse events (AEs), serious adverse events (SAEs), AEs that lead to discontinuation, study device-related AEs (determined by investigator), and AEs that lead to death will be summarized. Vital signs, hematology, and clinical chemistry parameters from baseline to the end of the study will be presented as outlined above for continuous outcomes and reported to the DSMB at the regularly scheduled meetings. Incidence of changes in laboratory parameters from normal to abnormal, based on Boston Children’s Hospital reference ranges, will be identified and lab values listed by subject. | |

TABLE OF CONTENTS

Page

[STUDY SYNOPSIS 3](#_Toc126862688)

[LIST OF TABLES 15](#_Toc126862689)

[LIST OF FIGURES 15](#_Toc126862690)

[LIST OF ABBREVIATIONS 16](#_Toc126862691)

[1.0 INTRODUCTION 17](#_Toc126862692)

[1.1 BACKGROUND 17](#_Toc126862693)

[1.2 STUDY RATIONALE 21](#_Toc126862694)

[2.0 OBJECTIVES 25](#_Toc126862695)

[2.1 PRIMARY OBJECTIVE 25](#_Toc126862696)

[2.2 SECONDARY OBJECTIVE 25](#_Toc126862697)

[2.3 SAFETY OBJECTIVES 25](#_Toc126862698)

[2.4 EXPLORATORY OBJECTIVES 26](#_Toc126862699)

[3.0 STUDY DESIGN 27](#_Toc126862700)

[3.1 BASIC DESIGN CHARACTERISTICS 27](#_Toc126862701)

[3.2 STUDY POPULATION 29](#_Toc126862702)

[3.2.1 Inclusion Criteria 29](#_Toc126862703)

[3.2.2 Exclusion Criteria 30](#_Toc126862704)

[3.3 ENDPOINTS 32](#_Toc126862705)

[3.3.1 Primary efficacy endpoint 32](#_Toc126862706)

[3.3.2 Secondary efficacy endpoint 32](#_Toc126862707)

[3.3.3 Safety endpoints 32](#_Toc126862708)

[3.3.4 Exploratory endpoints 32](#_Toc126862709)

[3.4 DROPOUTS 33](#_Toc126862710)

[4.0 STUDY DEVICE 35](#_Toc126862711)

[4.1 IDENTIFICATION AND DESCRIPTON OF INVESTIGATIONAL PRODUCT 35](#_Toc126862712)

[4.1.1 Investigational Product 35](#_Toc126862713)

[4.1.2 Labeling 36](#_Toc126862714)

[4.2 ADMINISTRATION INSTRUCTIONS AND SCHEDULE 36](#_Toc126862715)

[4.3 STORAGE AND HANDLING OF INVESTIGATIONAL PRODUCT 39](#_Toc126862716)

[4.4 COMPLIANCE WITH INVESTIGATIONAL PRODUCT 40](#_Toc126862717)

[4.5 CONCOMITANT MEDICATIONS 41](#_Toc126862718)

[5.0 MANAGEMENT OF NUTRITIONAL SUPPORT DURING THE STUDY 42](#_Toc126862719)

[5.1 STEPS FOR ADJUSTING NUTRITIONAL SUPPORT VOLUME AND CALORIES (Figure 2) 43](#_Toc126862720)

[6.0 EXPERIMENTAL PROCEDURES 44](#_Toc126862721)

[6.1 OVERVIEW: SCHEDULE OF TIME AND EVENTS 44](#_Toc126862722)

[7.0 MEASUREMENTS AND EVALUATIONS 48](#_Toc126862723)

[7.1.1 Screening Period (Day -3) 48](#_Toc126862724)

[7.1.2 Enrollment Period (Day -3 to -1) 49](#_Toc126862725)

[7.1.3 First Day of Treatment (Day 1) 49](#_Toc126862726)

[7.1.4 Treatment Period (Day 1 to 90) 51](#_Toc126862727)

[7.1.5 Standard of Care 53](#_Toc126862728)

[8.0 DATA AND SAFETY MONITORING BOARD (DSMB) 55](#_Toc126862729)

[9.0 PROCEDURES FOR HANDLING ADVERSE EVENTS AND SERIOUS ADVERSE EVENTS 55](#_Toc126862730)

[9.1 DEFINITION OF AN ADVERSE EVENT 55](#_Toc126862731)

[9.2 DEFINITION OF A SERIOUS ADVERSE EVENT 57](#_Toc126862732)

[9.3 DEFINITION OF UNANTICIPATED ADVERSE DEVICE EFFECT 59](#_Toc126862733)

[9.4 RECORDING ADVERSE EVENTS AND SERIOUS ADVERSE EVENTS 59](#_Toc126862734)

[9.5 ASSESSMENT OF INTENSITY 59](#_Toc126862735)

[9.6 ASSESSMENT OF CAUSALITY 60](#_Toc126862736)

[9.7 EXPECTEDNESS OF SERIOUS ADVERSE EVENTS 61](#_Toc126862737)

[9.8 REPORTING OF SERIOUS ADVERSE EVENTS 62](#_Toc126862738)

[9.9 FOLLOW-UP OF ADVERSE EVENTS AND SERIOUS ADVERSE EVENTS 63](#_Toc126862739)

[9.10 LIVER EVENTS 63](#_Toc126862740)

[9.11 TREATMENT INTERRUPTION 64](#_Toc126862741)

[Treatment interruption will be allowed for subjects who require temporary reduction or elimination of enteral nutrition due to intercurrent illness. The RELiZORB enzyme cartridge will continue to be used for any enteral nutrition that is administered during this time. Periods of intercurrent illness will be recorded on the adverse event form and addressed at the time of data analysis (see analysis section). 64](#_Toc126862742)

[9.12 SUBJECT DISCONTUNUATION 64](#_Toc126862743)

[9.12.1 Adverse Event 65](#_Toc126862744)

[9.12.2 Intercurrent Illness 65](#_Toc126862745)

[9.12.3 Noncompliance 65](#_Toc126862746)

[9.12.4 Refusal of Investigational Product Administration 65](#_Toc126862747)

[9.12.5 Withdrawal of Consent 66](#_Toc126862748)

[9.13 STOPPING RULES 66](#_Toc126862749)

[9.14 PREMATURE STUDY TERMINATION 66](#_Toc126862750)

[10.0 DATA COLLECTION AND PROCESSING AND STATISTICAL ANALYSIS 67](#_Toc126862751)

[10.1 DATA COLLECTION AND PROCESSING 67](#_Toc126862752)

[10.2 STATISTICAL ANALYSIS 68](#_Toc126862753)

[10.2.1 General Overview 68](#_Toc126862754)

[10.2.2 Outcomes Evaluation 68](#_Toc126862755)

[10.2.3 Safety Analysis 70](#_Toc126862756)

[10.2.4 Other considerations 71](#_Toc126862757)

[10.2.5 Sample Size 73](#_Toc126862758)

[10.3 INFORMED CONSENT AND AUTHORIZATION FOR USE AND DISCLOSURE OF PROTECTED HEALTH INFORMATION 74](#_Toc126862759)

[10.4 STUDY DOCUMENTATION 75](#_Toc126862760)

[10.4.1 Investigator Information 75](#_Toc126862761)

[10.4.2 Investigator’s Study Files 75](#_Toc126862762)

[10.4.3 Case Report Forms and Source Documentation 76](#_Toc126862763)

[10.4.4 Retention of Study Documents 76](#_Toc126862764)

[10.5 CONFIDENTIALITY 77](#_Toc126862765)

[10.5.1 Data 77](#_Toc126862766)

[10.5.2 Subject Anonymity 77](#_Toc126862767)

[10.6 PROTOCOL COMPLIANCE 77](#_Toc126862768)

[10.7 STUDY MONITOR FUNCTIONS AND RESPONSIBILITY 78](#_Toc126862769)

[10.8 GENERAL INFORMATION 78](#_Toc126862770)

[11.0 REFERENCES 78](#_Toc126862771)

LIST OF TABLES

Page

[Table 1. Fat hydrolysis for representative enteral formulas (500 mL) using RELiZORB at a flow rate of 120 mL/hour 22](#_Toc505707711)

[Table 2. Schedule of Time and Events 46](#_Toc505707712)

[Table 3. Daily Fluid Requirements by Body Weight, According to the Holliday-Segar Method 53](#_Toc505707713)

[Table 4. Daily Caloric and Protein Requirements 53](#_Toc505707714)

[Table 5. Nutritional Regimen 53](#_Toc505707715)

[Table 6. Classification of AEs by Intensity 60](#_Toc505707716)

[Table 7. Assessment of Causality of AEs 61](#_Toc505707717)

LIST OF FIGURES

Page

[Figure 1. Hydrolysis of fat by lipase into monoglyceride and free fatty acids 21](#_Toc126862772)

[Figure 2. Nutritional support adjustments based on calories 43](#_Toc126862773)

[Figure 3. Hypothetical examples of area under the curve for one subject 69](#_Toc126862774)

LIST OF ABBREVIATIONS

AE adverse event

ALT alanine aminotransferase

ARA arachidonic acid

AST aspartate aminotransferase

CRF case report form

DHA docosahexaenoic acid

DSMB data and safety monitoring board

EDC electronic data capture

EN enteral nutrition

EPA eicosapentaenoic acid

EPI exocrine pancreatic insufficiency

FDA Food and Drug Administration

GGT gamma-glutamyl transpeptidase

GMP good manufacturing practice

ICH International Council on Harmonization

IEC independent ethics committee

INR international normalized ratio

IP investigational product

IRB institutional review board

LA linoleic acid

LCPUFA long-chain polyunsaturated fatty acid

NORD National Organization of Rare Disorders

PN parenteral nutrition

PNALD PN associated liver disease

SAE serious adverse event

SBS short bowel syndrome

# INTRODUCTION

## BACKGROUND

Short bowel syndrome (SBS) is a rare condition included in The National Organization for Rare Disorders (NORD) database. The estimated prevalence of SBS in adults in the US is reported to be approximately 10,000-20,000 individuals (Oley Foundation [www.oley.org]; Short Bowel Syndrome Foundation [http://www.shortbowelfoundation.org]), affecting males and females in equal numbers. The disorder has numerous causes, both congenital and acquired, but a common etiological factor is the functional or anatomical loss of extensive segments of the small intestine, leading to a severe decrease in intestinal absorptive capacity. In children, the most common causes are necrotizing enterocolitis, intestinal atresias, and intestinal volvulus.^1^

The link between intestinal loss of the small bowel and the risk of SBS is self-evident. Losing large amounts of the small intestine compromises the digestive and absorptive processes. Adequate digestion and absorption cannot take place and proper nutritional status cannot be maintained. Treatment of SBS is focused on supplying the nutrients and vitamins that patients lack and may include (1) a high-calorie diet that includes vitamins, minerals, carbohydrates, proteins, and fats; (2) injections of vitamins and minerals; (3) administration of drugs that slow down the normal movement of the small intestine; and (4) feeding through a vein (i.e., parenteral nutrition (PN)).

Most SBS patients are initially fed with PN. In some patients, intestinal adaptation, alone or in combination with dietary modifications, allows weaning from PN. Unfortunately, many patients cannot be weaned from PN because of dramatically reduced intestinal length or function. Patients on long-term PN frequently experience serious metabolic complications. Most complications are hepatic and biliary disorders manifested by hepatic steatosis, fibrosis and cholestasis.[^1^](#_REFERENCES) These disorders can progress to fulminant liver failure.[^1^](#_REFERENCES) Before 2006, advanced liver disease was the most common cause of death in SBS patients.[^2^](#_REFERENCES) In infants with SBS, PN-associated liver disease (PNALD) is estimated to occur in 40-60% of those receiving long-term PN[^3,4^](#_REFERENCES) with mortality estimated to be as high as 80%.[^5,6,7^](#_REFERENCES)

Many consider PNALD to be caused in part by the intravenous lipid emulsions used to provide fat calories in the PN formulation, although the etiology is unclear.[^3,4,8^](#_REFERENCES) It has been suggested that PNALD may be caused by inflammation that is related to the type of intravenous lipid emulsion used.[^4,8^](#_REFERENCES) These fat emulsions cannot be eliminated from PN because by doing so would lead to the development of essential fatty acid deficiency and its concomitant complications.[^8,9^](#_REFERENCES) Recent data has shown that the replacement of a primarily omega-6 soybean oil lipid emulsion with those comprised primarily of omega-3 fatty acids may lead to reduction or avoidance of PNALD without predisposing the patient to essential fatty acid deficiency.[^3,10^](#_REFERENCES) Moreover, having a more bioavailable form of essential fatty acids that can be administered enterally may reduce or avoid the need for intravenous lipid emulsions entirely.

Although increased and earlier enteral feeding may reverse PNALD, data suggests that duration of hepatic dysfunction can last for several months after cessation of PN.[^11,12,13^](#_REFERENCES) In one retrospective study, children with a history of PNALD who have achieved PN independence had persistent alanine aminotransferase (ALT) elevations despite normalization of direct bilirubin concentrations. This suggests that hepatic injury may be ongoing beyond the time of bilirubin normalization.[^13^](#_REFERENCES) PN-dependent infants can achieve normalization of marked hyperbilirubinemia with enteral nutrition, however this improvement in liver function usually begins only after full enteral nutrition is tolerated and PN is withdrawn. These findings support the aggressive weaning of PN to enteral nutrition in infants with SBS. ^12^

Enterally administered long chain triglycerides in patients with SBS, especially those with hepatic dysfunction, are not well tolerated due to bile acid malabsorption, which leads to decreased micelle formation and fat digestion. Switching to other forms of fat such as medium-chain triglycerides that do not require micelles for absorption may be better tolerated in patients with bile acid or pancreatic insufficiency but are not optimal as they increase the osmotic load in the intestine. This may increase the chance of dumping; moreover, medium chain fatty acids do not contain essential fatty acids. The ability to provide the essential fatty acids such as those present in enteral formulas in a form that does not require the formation of micelles for absorption, would allow patients with SBS and those who are no longer PN dependent to receive adequate nutrition and continue to maintain the same growth trajectory as when they received the majority of their nutrition parenterally.

For example, enteral fish oil has been tried with limited success as an alternative to intravenous fish oil lipid emulsions in PN dependent infants with PNALD.^14^ In that case series, patients received a variety of products at various doses and actual absorption of the products was not determined. The results were further confounded by a variety of enteral feeding practices. Thus, no firm conclusions could be drawn from this retrospective study. In addition to improved hepatic function, early enteral fish oil administration may improve intestinal adaptation in infants with an enterostomy. Yang et al. have described how infants less than 2 months of age with an enterostomy who were tolerating some enteral nutrition (approximately 20 ml/kg/day) in combination with PN were shown to have increased enteral intake, better enteral protein absorption, decreased need for parenteral fat emulsions, and reduced conjugated bilirubin before reanastomosis and improved growth after reanastomosis.^15,16,17^ Furthermore, by improving tolerance of enteral feedings, bile flow can be stimulated and enterohepatic recirculation can be improved, such that PNALD can be prevented or reversed. Despite these findings, PN duration remained unchanged and actual fat reduction was more likely due to increased fat intake in the intervention arm and not due to improved absorptive capacity.

It is hypothesized that by using an external lipase device enteral nutrition will be better absorbed, and PN dependence will be reduced as enteral autonomy is increased. As mentioned previously, the goal of therapy for patients with SBS is to transition from parenteral nutrition to enteral nutrition. In one retrospective review conducted on infants at our institution's Short Bowel Syndrome Clinic from 1999 to 2004, the benefit of stopping PN and achieving enteral autonomy was demonstrated.^12^ Inclusion criteria included PN use for more than 1 month, serum direct bilirubin more than 3 mg/dL while on PN, and tolerance of full enteral nutrition with eventual discontinuation of PN. Twelve infants were identified with a PN duration of 5±1 months. Five patients underwent liver biopsy while on PN, and histological evidence of cholestasis was found on all specimens. Peak total and direct bilirubin levels were 10.5±1.9 and 7.0±1.6 mg/dL, respectively, and occurred at time of PN discontinuation. Only 2 patients had improvement in serum bilirubin levels before initiation of full enteral nutrition. After initiation of full enteral nutrition and discontinuation of PN, all patients achieved permanent normalization of bilirubin levels by 4 months (*P* < 0.05) after a 1-month plateau phase. Alkaline phosphatase levels approached reference range within this time but were not significant.

One of the challenges in providing enteral nutrition to patients with SBS is ensuring that the nutrition is adequately absorbed to achieve adequate growth and avoidance of deficiencies. The small intestine is the primary site of nutrient absorption; a decrease in absorptive capacity of nutrients in the small intestine may similarly impact the drug absorption process.

Alcresta Pharmaceuticals has developed a device which will assist in the absorption of enterally administered nutrition. RELiZORB is a single-use, point-of-care digestive enzyme cartridge that connects in-line with existing enteral feeding pump tubing sets and patient extension sets or enteral feeding tubes. RELiZORB is designed to hydrolyze (digest) fats contained in enteral formulas, mimicking the function of the digestive enzyme lipase that is normally secreted by the pancreas, the body’s digestive organ. By hydrolyzing (digesting) fats from enteral formulas, RELiZORB allows for the delivery of absorbable fatty acids and monoglycerides to patients. By virtue of its formulation and mechanism of action, it is hypothesized that RELiZORB will increase fatty acid absorption in patients with SBS and allow patients to achieve the same growth as that experienced while receiving parenteral nutrition. RELiZORB is therefore expected to increase intestinal nutrient absorption in SBS patients.

RELiZORB contains the digestive enzyme lipase bound to beads (iLipase®). Like human pancreatic lipase, the lipase in RELiZORB has sn-1, sn-3 selectivity in the hydrolysis of triglyceride fats. When enteral formula flows through RELiZORB, the lipase bound to the beads hydrolyzes fats in their triglyceride form, including important long-chain polyunsaturated fats (LCPUFAs), releasing omega-3 (docosahexaenoic acid (DHA) and eicosapentaenoic acid (EPA)) and omega-6 (linoleic acid (LA) and arachidonic acid (ARA)) into their absorbable fatty acid and monoglyceride forms. The iLipase® is retained within the RELiZORB cartridge by two filters as enteral formula flows through RELiZORB.

Figure 1. Hydrolysis of fat by lipase into monoglyceride and free fatty acids


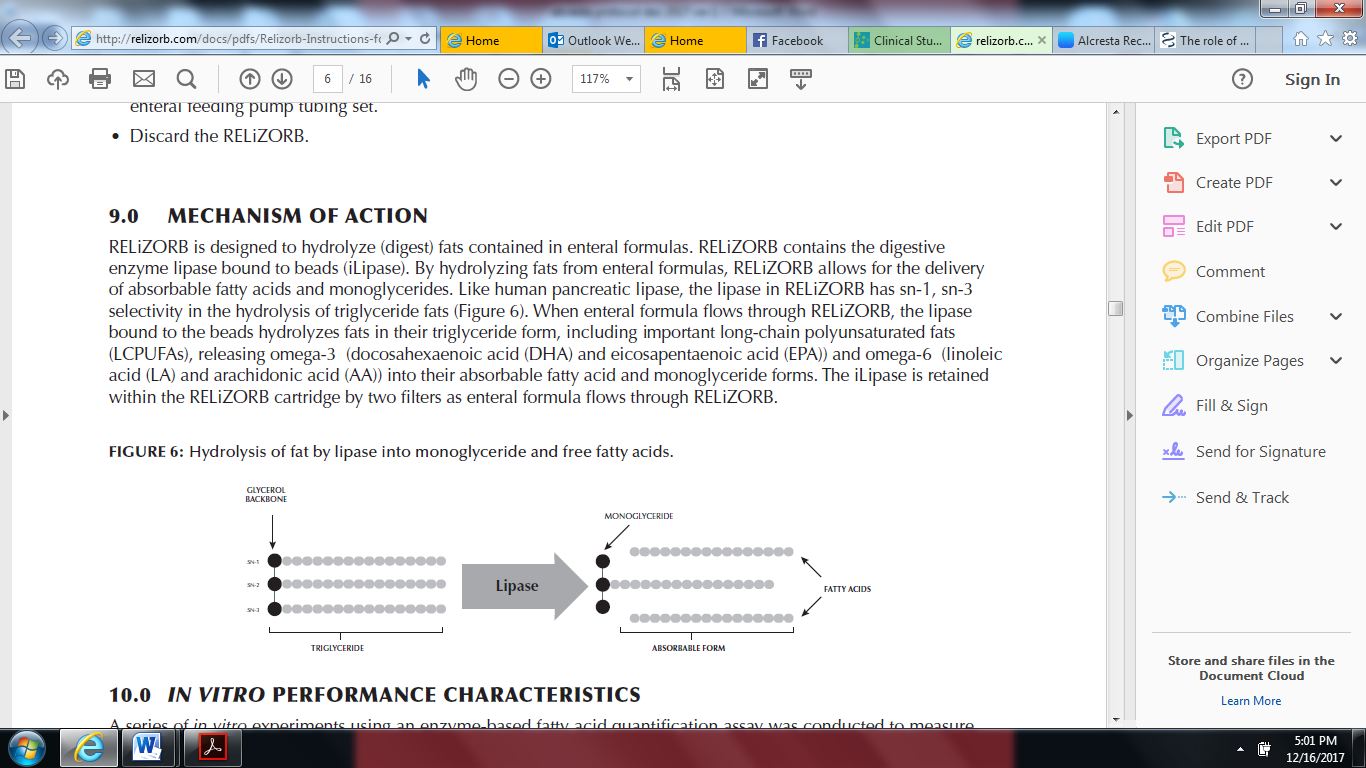


Medications that have been used to manage SBS include teduglutide, a form of glucagon-like peptide 2, which is a protein involved in the adaptation and rehabilitation of the lining (mucosa) of the intestines, and somatropin (rDNA origin) for injection, a human growth hormone, thought to improve the ability of the small intestine to absorb nutrients, thereby lessening the need for PN. Both of these therapies are not without risk. Teduglutide carries a risk for acceleration of neoplastic growth whereas somatropin use has been associated with an increased risk of mortality. There is a need for a less toxic therapy.

## STUDY RATIONALE

Previous in vitro and pre-clinical studies have provided the scientific rationale for the use of RELiZORB for the treatment of SBS. RELiZORB was approved by the FDA for use in children older than age 5 and adults to hydrolyze fats in enteral formula. Furthermore, the FDA has reviewed this study and determined it to be nonsignificant risk.

***Representative Fat Hydrolysis Data Using RELiZORB***

RELiZORB has been shown to hydrolyze >90% of fat in most enteral formulas when tested with 500 mL of enteral formula at a flow rate of 120 mL/hour (Table 1). Comparable or improved fat hydrolysis has also been shown when RELiZORB was used with enteral feeding pump flow rates between 10-120 mL/hour.

Table 1. **Fat hydrolysis for representative enteral formulas (500 mL) using RELiZORB at a flow rate of 120 mL/hour**

| **PRODUCT** | **FAT (g)** | **MCT / LCT RATIO (%)** | **CALORIES (kcal)** | **OMEGA-3 DHA & EPA (g)** | **% FAT HYDROLYSIS WITH RELiZORB** |
| --- | --- | --- | --- | --- | --- |
| **NUTREN** **2.0** | 52 | 74:25 | 1,000 | - | >90% |
| **TWOCAL** **HN** | 43 | 19:81 | 950 | - | 60% |
| **IMPACT** **PEPTIDE 1.5** | 32 | 50:50 | 750 | 2.45 | >90% |
| **PEPTAMEN** **1.5** | 28 | 70:30 | 750 | - | >90% |
| **PEPTAMEN AF** | 27 | 50:50 | 600 | 1.2 | >90% |
| **VITAL AF 1.2 CAL** | 26 | 45:55 | 570 | 1.9 | >90% |
| **PEPTAMEN** | 20 | 70:30 | 500 | - | 90% |
| **NUTREN**® **1.0** | 19 | 25:75 | 500 | - | >90% |

(For a detailed list of compatible formulas refer to <https://www.relizorb.com/docs/pdfs/Compatible-Formulas-and-Pumps.pdf>, accessed September 29, 2020)

The efficacy, safety and intended use of RELiZORB was tested in a well-established pre-clinical porcine model of exocrine pancreatic insufficiency (EPI) that mimics the inability to digest and absorb fats.^18^ Ligation of the pancreatic ducts in the EPI porcine model causes a total lack of pancreatic enzymes, leading to arrested growth, fatty acid deficiencies, and GI symptoms including steatorrhea.

In a study conducted in the EPI porcine model, a single feeding (500 mL; 4 hours, 120 mL/hr) of enteral formula administered through RELiZORB resulted in an increase in fat absorption, caloric intake and improvement in uptake of omega-3 fatty acids docosahexaenoic acid (DHA) and eicosapentaenoic acid (EPA) in plasma levels over 24 hours compared with enteral formula that was not administered through RELiZORB. Enteral formula passed through RELiZORB normalized plasma levels of DHA and EPA compared to the healthy control group. RELiZORB was shown to hydrolyze >90% of fats from 500 mL of Peptamen AF® (750 kcal, 32 g fat, 1.8 g DHA and EPA; Nestlé Health Science Europe). The clinical significance of these findings has not been determined.

The objective of this study (i.e., *24 Hour Pharmacokinetic Plasma Fatty Acid Uptake: Safety, tolerability and fat absorption with use of RELiZORB in enteral (tube) feeding*) was to measure fat absorption by assessing plasma 24-hour pharmacokinetic changes evaluated by measuring important plasma long-chain polyunsaturated fatty acids (LCPUFA), specifically omega-3 fatty acids DHA and EPA. This was a parallel study consisting of three groups: an EPI group (n=6) receiving formula hydrolyzed with RELiZORB, a second EPI group (n=5) receiving non-hydrolyzed formula without RELiZORB and a third healthy control group (n=3) with normal digestive function receiving non-hydrolyzed formula without RELiZORB. Peptamen® AF, a semi-elemental enteral formula containing hydrolyzed protein was provided over 4 hours (500 mL; pump rate 120 mL/hour). Fat absorption was evaluated by measuring plasma long-chain polyunsaturated fatty acids (LCPUFA), specifically plasma DHA and EPA levels. RELiZORB use was well tolerated in the porcine model with normal food intake and no serious adverse reactions. No vomiting or diarrhea was recorded during the 24-hour period. RELiZORB normalized plasma levels of DHA and EPA compared to the healthy control group. Formula hydrolyzed with RELiZORB was associated with a statistically significant increase in total fat absorption and improvement in uptake of omega-3 fatty acids (DHA and EPA) in plasma levels over 24-hours compared with non-hydrolyzed formula without RELiZORB (*P* < 0.05). The clinical significance of this observation has not been determined. Increased uptake of specific long-chain polyunsaturated fatty acids with use of RELiZORB resulted in a statistically significant reduction in the omega-6 to omega-3 ratio. The clinical significance of this observation has not been determined.

Based on the EPI porcine model data, the FDA approved RELiZORB in November 2015 for use in adults to hydrolyze fats in enteral nutrition. The first clinical study (*Increased Fat Absorption from Enteral Formula through an In-line Digestive Cartridge in Patients with Cystic Fibrosis*) assessed the safety, tolerability and fat absorption of RELiZORB in patients with cystic fibrosis (CF). This was a multicenter, randomized, double-blind, crossover trial with an open-label safety evaluation period. The study was comprised of a 7-day run-in period, a cross-over period, and a 7-day open-label safety period. Patients were randomized to receive RELiZORB or a placebo cartridge. The primary efficacy endpoint was the change in plasma fatty acid concentrations of DHA and EPA, markers of fat absorption, for 24 hours after a single enteral nutrition feeding administered through the in-line device. Safety and tolerability endpoints included gastrointestinal (GI) events associated with fat malabsorption, as well as non-GI events.

Thirty-three patients were enrolled from 11 CF centers across the United States; 82% of enrolled patients were ≤18 years of age. The mean area under the curve for total DHA + EPA was 2.8 times higher for RELiZORB compared to placebo (537.0±400.5 vs 192.2±198.7 µg/mL, respectively; *P*<0.001). RELiZORB was generally safe and well tolerated, with no unanticipated adverse effects.

Freedman et al evaluated safety, tolerability, and fat absorption of the RELiZORB enzyme cartridge in 33 patients aged 5 – 34 years with cystic fibrosis^20^. In this multicenter, randomized, double-blind, crossover trial, plasma omega-3 fatty acid concentrations were used to measure fat absorption. The RELiZORB enzyme cartridge was associated with a 2.8-fold increase in plasma omega-3 fatty acid concentration compared to placebo and was determined to be safe and well-tolerated.

In another study by Stevens et al, 36 cystic fibrosis patients aged 5 – 33 years were followed longitudinally for 90 days in a multicenter, open-label study of RELiZORB use with overnight enteral nutrition^21^. The RELiZORB enzyme cartridge was associated with a 2-fold increase in the omega-3 index, a long-term measure of fat absorption in patients with cystic fibrosis. No unexpected adverse events were associated with the use of the RELiZORB cartridge.

# OBJECTIVES

## PRIMARY OBJECTIVE

- To determine the effectiveness of the RELiZORB enzyme cartridge on the absorption of enteral nutrition when used daily for a total 90 days of treatment, in pediatric subjects with SBS who are PN dependent, aged 2 years – 18 years, by measuring the change in PN calories from baseline, assessed weekly throughout the study.

## SECONDARY OBJECTIVE

- To determine the effectiveness of the RELiZORB enzyme cartridge on the absorption of enteral nutrition when used daily for a total 90 days of treatment, in pediatric subjects with SBS who are PN dependent, aged 2 years – 18 years, by measuring the change in body weight (weight-for-age z-score) from baseline, assessed weekly throughout the study.

## SAFETY OBJECTIVES

- To determine the safety and tolerability of the RELiZORB enzyme cartridge on the absorption of enteral nutrition when used daily for a total 90 days of treatment, in pediatric subjects with SBS who are PN dependent, aged 2 years -18 years, by recording daily the incidence of adverse events, including changes in stool consistency (through Bristol scale), changes in stool amount/frequency (if applicable), increased ostomy output (if applicable, defined as >2.5 mL/kg/hour), the need to decrease enteral feeds, changes in urine color (through Dehydration Color scale), and discontinuation from study treatment.
- To determine the safety and tolerability of the RELiZORB enzyme cartridge on the absorption of enteral nutrition when used daily for a total 90 days of treatment, in pediatric subjects with SBS who are PN dependent, aged 2 years -18 years, by monitoring vital signs (blood pressure, pulse rate, respiratory rate, temperature).
- To determine the safety and tolerability of the RELiZORB enzyme cartridge on the absorption of enteral nutrition when used daily for a total of 90 days of treatment, in pediatric subjects with SBS who are PN dependent, aged 2 years – 18 years, by monitoring hematology and biochemistry parameters. Clinical lab evaluations will include sodium, potassium, chloride, CO2, glucose, blood urea nitrogen (BUN), creatinine, calcium, phosphorus, magnesium, alkaline phosphatase, albumin, pre-albumin, total protein, total and direct bilirubin, AST, ALT, GGT, triglycerides, cholesterol, high-density lipoprotein (HDL), very low-density lipoprotein (VLDL), low-density lipoprotein (LDL), C-reactive protein (CRP), and fatty acid profile.

## EXPLORATORY OBJECTIVES

- To evaluate the effect of using the RELiZORB enzyme cartridge with enteral nutrition when used daily for a total 90 days of treatment, on the change in fat absorption as measured by 72-hour total fecal fat and fatty acid composition in pediatric subjects with SBS who are PN dependent, aged 2 years – 18 years.
- To evaluate the effect of using the RELiZORB enzyme cartridge with enteral nutrition when used daily for a total 90 days of treatment, on the change in plasma fatty acid composition and fat-soluble vitamins (A, D, E, and K) in pediatric subjects with SBS who are PN dependent, aged 2 years – 18 years.
- To evaluate the effect of using the RELiZORB enzyme cartridge with enteral nutrition when used daily for a total 90 days of treatment on growth in pediatric subjects with SBS who are PN dependent, aged 2 years – 18 years, by measuring height (assessed with height-for-age z-score) and head circumference (assessed with head circumference-for-age z-score in children < 36 months of age).
- To evaluate the effect of using the RELiZORB enzyme cartridge with enteral nutrition when used daily for a total 90 days of treatment on BMI z-score in pediatric subjects with SBS who are PN dependent, aged 2 years – 18 years.
- To evaluate the effect of using the RELiZORB enzyme cartridge with enteral nutrition when used daily for a total 90 days of treatment on PN dependency in pediatric subjects with SBS who are PN dependent, aged 2 years – 18 years, by recording PN volume.
- To evaluate the effect of using the RELiZORB enzyme cartridge with enteral nutrition when used daily for a total of 90 days of treatment on enteral nutrition tolerance in pediatric subjects with SBS who are PN dependent, aged 2 years – 18 years, by recording total enteral calories and volume.
- To evaluate the effect of using the RELiZORB enzyme cartridge with enteral nutrition when used daily for a total of 90 days of treatment on oral nutrition tolerance in pediatric subjects with SBS who are PN dependent, aged 2 years – 18 years, by recording total oral calories and volume.
- To evaluate the effect of using the RELiZORB enzyme cartridge with enteral nutrition when used daily for a total of 90 days of treatment on the ability to wean from PN at any time during the course of the study.

# STUDY DESIGN

## BASIC DESIGN CHARACTERISTICS

This study is a phase 3, open labeled uncontrolled single center,
clinical trial to determine the safety, tolerability, and bioavailability of the RELiZORB enzyme cartridge with enteral nutrition when used daily for a total 90 days of treatment when given to pediatric subjects with SBS, aged 2 years – 18 years, who have been PN dependent for at least 6 months.

Subjects with a signed informed consent will be enrolled in the study. Results from screening must not show any clinically significant abnormalities in order for the subject to progress to the enrollment and treatment phases of the trial. During the screening visit, subjects and legal guardians will also receive education from a registered dietician on recording caloric intake and composition in oral and enteral feeds, and PN. As part of this education, the subject and parent/guardian will receive access to a nutrition/food tracking phone application (Cronometer) to assist them with this task throughout the study. If there is no electronic capability, a paper diary will be provided. The subject and parent/guardian will also receive education from a registered nurse on accurate and complete stool collection. Subjects will be provided with a standardized weight scale for at-home weight recording.

Upon completing screening and enrollment, subjects and their legal guardians will be instructed to record total calories and composition in oral and enteral feeds and TPN and to collect all stool for 72 hours (Days -3 to -1) prior to the first day of treatment. The stool collection and recording will be repeated in the 72-hours preceding the final study visit (Days 87-89). All stool will be collected in a single container and kept refrigerated during collection. If necessary (for example, out of state participants, excessively watery stools), subjects will be admitted to our dedicated research center during this collection period.

On the first day of treatment (Day 1), all subjects will use the RELiZORB enzyme cartridge to administer their enteral nutrition and will continue to use a cartridge daily for a total of 90 days. The study device will be used with enteral nutrition administered to the subject daily by a parent or guardian for 90 days (Day 1 to Day 90).

Nutritional intake (24 hour enteral and oral intake, and PN volume) will be recorded daily in the nutrition/food tracking phone application (or paper diary). Any changes in symptoms (e.g., abdominal pain, increases in stool output above baseline, changes in urine frequency/color) will also be recorded daily by the subject’s parent or guardian in an electronic diary (or paper diary). Families will also receive a daily study coordinator phone call to address any issues or concerns and ensure compliance. Subjects will visit the clinic on Day 1, Day 7, Day 14, Day 28, Day 60, and Day 90 of the treatment period, where clinical and laboratory assessments will be collected for the clinical and safety endpoints. Subjects will also receive continuing nutrition education during these visits and any questions will be addressed. The study staff (dietician, investigators) will have weekly telephone contact with families during interim weeks after the day 14 visit. Study staff will monitor safety, nutrition tracking, diaries, weekly weights, stool composition (Bristol scale) and output, changes in caloric intake, changes in urine output, and will make adjustments to PN and enteral feeding accordingly. Adjustments will be based on the subject’s nutritional needs, weight, height, hydration status and investigator’s medical judgement. If necessary, unscheduled visits can be arranged in place of the telephone contacts.

## STUDY POPULATION

A total of 32 pediatric subjects with SBS, aged 2 years – 18 years and PN dependent are expected to be enrolled in this open labeled study. Eligibility will be established by the investigator based on the inclusion and exclusion criteria.

### Inclusion Criteria

To be considered eligible to participate in this study, a subject must meet all the inclusion criteria listed below:

1. Male or female patients, aged 2 years – 18 years, inclusively.
2. Diagnosed with SBS, as determined by medical history and PN dependence (i.e. need for PN for >60 days after intestinal resection or a bowel length <25% of expected).
3. Congenital or acquired gastrointestinal disease requiring surgical intervention that has occurred at least 3 months prior to screening.
4. Patient is on parenteral lipid and at least 30% of daily caloric and fluid intake has been provided by PN for at least 6 months prior to screening.
5. Stable PN nutrition requirement, determined by less than 5% reduction in PN nutrition for at least 1 month prior to screening, or at the discretion of the investigator.
6. The patient has a Central Venous Catheter (CVC) at the time of study inclusion.
7. Screening direct bilirubin that is in the normal range for age or is not determined to be clinically significant by the investigator.
8. The patient has an existing feeding tube, receiving enteral nutrition via an enteral feeding pump at a rate>10ml/hr but <120ml/hr, and is able to tolerate at least 10 ml/kg/day enteral nutrition with which the RELiZORB cartridge can be used.
9. Stable enteral nutrition requirement with no change in formula composition or rate for at least 1 month prior to screening.
10. The patient or a parent or legal guardian of the patient is able to read, understand, and is willing to provide informed consent (or assent, if applicable) for the patient.
11. The patient (if assent is applicable) or a parent or legal guardian is able to understand the requirements of the study and is willing to bring the patient to all clinic visits and complete all study related procedures (as determined by the investigator).
12. A parent or legal guardian is willing to provide written authorization for the use and disclosure of protected health information.

### Exclusion Criteria

To be eligible for entry into the study, the subject must not meet any of the exclusion criteria listed below:

1. Other causes of chronic liver disease other than SBS (i.e. hepatitis C, cystic fibrosis, biliary atresia, alpha 1 anti-trypsin deficiency, and Alagille syndrome).
2. The patient has had a bowel lengthening procedure, including but not limited to, a STEP procedure.
3. Any serum triglyceride concentration >400 mg/dL at screening.
4. Pancreatic insufficiency as defined as the use of pancreatic enzymes within 30 days prior to screening.
5. Evidence of untreated intestinal obstruction or active stenosis, as determined by the investigator.
6. Unstable absorption due to cystic fibrosis or known DNA abnormalities (i.e., familial adenomatous polyposis, Fanconi syndrome) as determined by the investigator.
7. History of microvillus inclusion disease, as determined by medical history.
8. Severe known dysmotility syndrome (i.e., pseudo-obstruction, gastroschisis-related motility disorders), as determined by the investigator.
9. Initiation of teduglutide or other GLP-2 analogues within 6 months of screening
10. Use of growth hormone, or supplemental glutamine within 3 months prior to screening.
11. Use of cisapride within 30 days prior to screening.
12. Active clinically significant pancreatic or biliary disease, as determined by the investigator.
13. Receiving enteral nutrition via any formula that is not compatible with the RELiZORB cartridge (for example, insoluble fiber-containing formulas).
14. Determined by the investigator to be unsuitable for participation in this trial for any reason.

## ENDPOINTS

### Primary efficacy endpoint

The primary efficacy endpoint is the change in PN calories since baseline, assessed weekly throughout the study.

### Secondary efficacy endpoint

The secondary efficacy endpoint is the change in body weight (weight-for-age z-score) since baseline, assessed weekly throughout the study.

### Safety endpoints

The safety endpoints include:

- Subject incidence of adverse events, including changes in stool consistency assessed by Bristol scale, changes in stool amount/frequency (if applicable), increased ostomy output (if applicable, defined as >2.5 mL/kg/hour), the need to decrease enteral feeds, changes in urine color assessed by Dehydration Color scale, and discontinuation from the study treatment.
- Subject incidence of abnormalities in vital signs, assessed at baseline, Day 7, 14, 28, 60, and 90.
- Subject incidence of abnormalities in hematology and biochemistry parameters assessed at baseline, Day 7, 14, 28, 60, and 90.

### Exploratory endpoints

- Change in 72-hour fecal fat composition and coefficient of fat absorption from enrollment, assessed at Days -3 to -1, to Days 87-89.
- Change in plasma serum fatty acid profile, and fat-soluble vitamins (A, D, E, and K) from baseline, assessed at Day 7, 14, 28, 60, and 90.
- Change in height (height-for-age z-score) and head circumference (head-circumference-for-age z-score) [in subjects < 36 months]) from baseline, assessed at Day 7, 14, 28, 60, and 90.
- Change in BMI z-score from baseline, assessed at Day 7, 14, 28, 60 and 90.
- Change in PN volume from baseline, assessed weekly.
- Change in enteral nutrition tolerance (calories and volume) from baseline, assessed weekly.
- Change in oral nutrition tolerance (calories and volume) from baseline, assessed weekly.
- Ability to wean from PN, assessed over the entire course of the study.

## DROPOUTS

Subjects who are enrolled but do not receive the RELiZORB enzyme cartridge will be considered dropouts. Furthermore, enrolled subjects who receive the RELiZORB enzyme cartridge and do not complete any study assessments through Day 90 will be considered dropouts. Dropouts will be replaced in order to reach the goal of 32 subjects with evaluable data. Patients with at least one post-baseline study assessment will be retained for analysis.

**3.5** **STUDY VISIT COMPENSATION**

Subjects will be paid $100.00 compensation for each research visit that they complete, with compensation provided through a service called ClinCard. Each subject/family will also be provided with a parking voucher and food voucher for each visit or will be reimbursed through the use of the ClinCard at the end of each study visit for study-related expenses such as travel, parking and meals, and other study-related expenses if a receipt is provided.

Subjects/families traveling a radius greater than 50 miles from BCH will be compensated for their travel expenses. Patients and families will be reimbursed for roundtrip travel expenses (i.e., airfare, gasoline, parking) and hotel stays at the Inn at Longwood Medical (or up to $225.00 a night if subjects stay at another hotel). Excluding travel expenses (if necessary) this will add up to a total of $1,402.00 if the subject completes all the research visits. If the subject leaves the study early, or is withdrawn at the discretion of the study physician, the subject will only be paid for the visits he/she has completed.

This research study will use a service called ClinCard® by the company Greenphire, www.greenphire.com, to manage all payments associated with the subject’s participation in study visits, time and travel related to participation in the study. ClinCard/Greenphire will provide documentation for the subject’s taxes (1099 form) and to the hospital. Boston Children’s Hospital has contracted with ClinCard/Greenphire to provide this service but Boston Children’s Hospital and ClinCard/Greenphire are separate entities and have no other relationship. ClinCard/Greenphire is solely responsible for the security of any information provided to them.

At each visit, the subject will receive a parking voucher and $25 for meal expenses. This $25 will also be loaded onto the ClinCard at each visit. If the subject resides out of state, the travel expenses as described above will be covered. The subject will also be compensated for completing the study diaries. The subject will be reimbursed $5.00 per completed diary.

If subjects live outside a 50-mile radius of BCH and travel to their study visits by car, they will be reimbursed for their round trip. We will calculate the appropriate reimbursement based on the IRS standard mileage rate for the calendar year and add that amount to the subject’s ClinCard.

Compensation Break Down

| **Item** | **Cost per day** | **# of days** | **Total** |
| --- | --- | --- | --- |
| Patient Stipend | $100 | up to 7 | $700 |
| Diary Completion | $5 | up to 90 | $450 |
| Parking Voucher | $11 | up to 7 | $77 |
| Meal Voucher | $25 | up to 7 | $175 |
| Total | | | $1402 |

# STUDY DEVICE

## IDENTIFICATION AND DESCRIPTON OF INVESTIGATIONAL PRODUCT

All investigational products used in this study will be manufactured, tested, labeled, and released according to current legal requirements and Good Manufacturing Practice (GMP).

### Investigational Product

RELiZORB is a single-use, point-of-care digestive enzyme cartridge that connects in-line with existing enteral feeding pump tubing sets and patient extension sets or enteral feeding tubes. RELiZORB is designed to hydrolyze (digest) fats contained in enteral formulas, mimicking the function of the digestive enzyme lipase that is normally secreted by the pancreas, the body’s digestive organ. By hydrolyzing (digesting) fats from enteral formulas, RELiZORB allows for the delivery of absorbable fatty acids and monoglycerides to patients.

RELiZORB is comprised of a clear cylindrical, plastic cartridge with a single inlet connection port and a single purple outlet connection port. The inlet and outlet ports of RELiZORB are intended to connect in-line with enteral feeding pump tubing sets and patient extension sets or enteral feeding sets. Inside the cartridge, there are small white beads. The digestive enzyme, lipase, is covalently bound to the small white beads. The lipase-bead complex, iLipaseTM (immobilized lipase), is retained within the cartridge during use by filters on both ends of the cartridge. The fat in enteral formulas is hydrolyzed as it comes in contact with iLipase as the formula passes through the cartridge.

### Labeling

All study devices will be labeled in accordance with text that is in full compliance with FDA regulations. Each study device label includes the following information:

- Protocol number
- Name and address of IP developer/distributor (Alcresta)
- Manufacturing date
- Route of administration and dosing (directed per protocol)
- Storage conditions
- Investigational device statement

## ADMINISTRATION INSTRUCTIONS AND SCHEDULE

- - - RELiZORB is intended for single-use only. At the conclusion of the feeding, the RELiZORB must be discarded. Do not store or re-use it.
    - A single RELiZORB may be used for up to 500 mL of enteral formula. For enteral formula volumes greater than 500 mL, install a new RELiZORB after 500 mL of formula has passed through the RELiZORB. A second RELiZORB may be used immediately after the first RELiZORB has been used. If less than 500 mL of enteral formula per feeding is used, the RELiZORB must be discarded after use.

Patients and patient caregivers should review the following RELiZORB installation instructions before use:

1. Set up the pump and enteral feeding pump tubing set per the pump manufacturer’s instructions. Prime the enteral feeding pump tubing per the manufacturer’s instructions.

2. Remove the RELiZORB pouch from its carton. Examine the RELiZORB pouch.

Do not use the RELiZORB if:

- the pouch seal is broken
- the current date is past the expiration date shown on the pouch

3. Remove the RELiZORB from its pouch. Examine the RELiZORB.

Do not use the RELiZORB if:

- the RELiZORB is damaged
- the RELiZORB has been previously used

4. Secure the RELiZORB to the end of the enteral feeding pump tubing set by inserting the outlet fitting from the pump tubing into the inlet of the RELiZORB with a twisting motion until secure.

5. Follow the pump manufacturer’s instructions to prime the feeding formula through to the outlet of the RELiZORB.

6. Connect the RELiZORB outlet fitting to the inlet fitting of the patient extension set or enteral feeding tube that connects to the patient.

7. If a patient extension set is being used, follow the pump manufacturer’s instructions to prime the feeding formula to the end of the patient extension set.

8. Set the pump to the prescribed flow rate between 10 and 120 mL/hr and proceed with feeding.

Feedings should be based on PI’s assessment of the patient’s daily needs and tolerance. Instructions will be provided to empty the feeding bag over a period of no more than 4 hours. The entire contents of the bag should be used to deliver the maximum dose possible. The volume of any remaining contents will be measured and recorded in the patient’s diary.

**Medication Administration:**

If medications, saline flushes or other non-enteral formula materials are to be added, they must be introduced AFTER RELiZORB (i.e. between RELiZORB and the patient). They may be added to the side-port of a Y-connector extension set located between the RELiZORB and the patient as shown below.

NOTE: If medications or flush solutions are added BEFORE the RELiZORB cartridge, then RELiZORB, all tubing and formula must be discarded. The feeding may be re-started using a new RELiZORB and patient extension set. Please follow Steps 1-7 above to re-start the process.

NOTE: If a second RELiZORB is required to be installed to replace an existing RELiZORB, use the following steps:

- Pause the pump following the pump manufacturer’s instructions
- Disconnect the current RELiZORB from the patient extension set or enteral feeding tube
- Remove the current RELiZORB from the enteral feeding pump tubing set
- Connect the new RELiZORB to the enteral feeding pump tubing set following Step 4 above
- Prime the enteral formula through to the end of the RELiZORB following Step 5 above
- Connect the new RELiZORB to the patient extension set or enteral feeding tube following Step 6 above
- Follow Step 7 above if a patient extension set is being used
- Follow Step 8 above to re-start enteral formula delivery

**Additional Information:**

- RELiZORB is for use with enteral feeding only.
- RELiZORB should not be connected to any intravenous (IV) line, set up, or system.
- Medications should not be administered through the RELiZORB cartridge. Do not add medications to the enteral feed line in between the pump and RELiZORB (before RELiZORB). The passage of medications through RELiZORB may adversely affect the medications or the ability of RELiZORB to hydrolyze fats.
- Do not re-use RELiZORB. RELiZORB is a single-use product. Re-use may result in contamination of the product. If re-used, RELiZORB may not effectively hydrolyze fats.
- Do not break, alter, or place excess pressure on any part of the RELiZORB cartridge. Any compromise of the structural integrity of RELiZORB may lead to improper connection to enteral feeding pump tubing sets and patient extension sets or enteral feeding tubes, enteral formula leakage or risk of contamination.
- Do not use RELiZORB after the date marked on the pouch.
- Enteral formulas containing insoluble fiber should NOT be used. Insoluble fiber may clog the RELiZORB cartridge. A detailed listing of enteral formulas compatible with RELiZORB can be found at www.relizorb.com/formulas.
- RELiZORB is designed for use with enteral feeding pump systems with low flow/no flow alarms. RELiZORB is NOT intended for use with gravity feed systems. A detailed listing of pumps, enteral feeding pump tubing sets and patient extension sets or enteral feeding tubes compatible with RELiZORB can be found at www.relizorb.com/pumps. RELiZORB has been evaluated with enteral pump flow rates between 10 and 120 mL/hr.
- Up to 2 RELiZORBs can be used in a day (24-hour period) and there are no requirements on the amount of time between using them

Further details on dosing instructions and schedule can be found in the Study Procedures Manual.

## STORAGE AND HANDLING OF INVESTIGATIONAL PRODUCT

Study device will be packaged, labeled, and shipped to the study site by the Alcresta Pharmaceuticals, Inc. Study device will be supplied in kits containing the required number of devices and labeled. The label on the kit includes a tear-off portion to be affixed to the subject’s source document or label page provided for this purpose.

Study device will be stored securely on site, in an area only accessible by site staff, at 15°C to 30°C. The subject’s parent or guardian will be instructed to keep the subject’s study device at room temperature: 15°C to 30°C. Parents or guardians will be instructed to return any used study devices at each clinic visit. Damaged or suspect study device will be returned unused to Alcresta. No special handling of the study device is required.

All study device supplies will be accompanied by accountability and shipping documents that will be maintained by the investigator or designee. Information recorded on these accountability and shipping documents will include relevant dates, batch numbers, quantities received or dispensed, to whom dispensed, returned device(s), and indication of device lost or damaged. Pharmacy dispensing records will be reviewed by a study monitor periodically to ensure adherence with procedures for dispensing of RELiZORB. At the end of the study, all used and unused RELiZORB will be accounted for. The remaining study device supplies will be destroyed on site.

## COMPLIANCE WITH INVESTIGATIONAL PRODUCT

The subject’s parent or guardian will be required to document the subject’s daily nutritional intake (24 hour oral and enteral caloric intake/composition and PN volume) using an electronic phone-based application (Cronometer; or paper diary if there is no electronic capability). The study device use and incidence of any changes in symptoms (e.g., abdominal pain, increases in stool output above baseline) will be recorded using the electronic diary (or paper diary). The subject’s parent or guardian will receive education and continued support from a study dietician to assist them in keeping track of nutritional and total caloric intake, as well as caloric composition in the diary throughout the duration of the study. Access to the electronic nutrition application (Cronometer) will facilitate this recording. The first and final clinic visits will be preceded by a required 72-hour stool collection for fat analysis.

The study staff will review the diaries and question the subject or the subject’s parent or guardian about protocol compliance at the clinic and weekly telephone visits. For hospitalized subjects, compliance will be assessed through daily monitoring of nutritional markers and review of medication administration records which utilize point of care bar code technology. Details of the study device use will be recorded on the CRF.

In the event that an out-of-state study patient is hospitalized at a hospital other than Boston Children’s and to ensure patients are able to remain in the study at an outside institution, at the time the patients are enrolled in the study, they will be provided a copy of their signed consent and the study protocol with study team contact information with instructions to bring these materials, along with the supply of RELiZORB devices, to the hospital so that they can use them in the event of an inpatient admission.

If a subject fails to comply with protocol requirements, the subject may be discontinued from the study. Compliance will be considered to have been achieved if the subject receives ≥80% of the planned enteral nutrition using the device.

## CONCOMITANT MEDICATIONS

Prohibited medications or therapies and restrictions for any drugs prior to receiving the open labeled study device are provided in the sections for inclusion criteria and exclusion criteria. A complete list of current medications will be ascertained at enrollment and each successive study visit.

Patients using enteral bile salt sequestering agents (e.g., cholestyramine, colestipol, colesevelam) at enrollment must continue to receive them for the duration of the 90 day study period. In patients who are not receiving these medications at enrollment, they cannot be initiated until the patient has completed the study. If a patient does start a bile salt sequestering agent during the study, they must be withdrawn from the study.

Patients will continue to receive the same lipid emulsion product throughout the study period. If a patient switches lipid emulsion products during the study period, they will be withdrawn from the study.

Patients who are using medications such as probiotics, antibiotics utilized for small intestinal bacterial overgrowth, fiber supplementation, and/or proton pump inhibitors must continue to receive them for the duration of the 90 day study period. In patients who are not receiving these medications at baseline, they cannot be initiated until the patient has completed the study. If the patient does start these medications during the study, they must be withdrawn from the study.

- 1. **INPATIENT USE**

In the event the patient does get admitted to hospital during the study period, they will be allowed to continue to use the RELiZORB device to administer their enteral nutrition. Since 2017, RELiZORB has been approved for use in Boston Children’s Hospital without any restrictions to nursing unit, patient population or clinician group. Copies of the Patient Care Manual policy and corresponding reference tool are located in the appendix.

# MANAGEMENT OF NUTRITIONAL SUPPORT DURING THE STUDY

Clinical parameters for management of pediatric short bowel syndrome will be considered for advancing nutritional support (PN/IV and/or oral/enteral feeding) in volume and calories during the 90 day study. Adjustment of nutritional support in volume and calories will be made at all planned study visits and during weekly phone calls as deemed appropriate (Figure 2). Clinical parameters to be considered include the following:

- Maintaining growth (including weight and height) by the subjects trending along their growth chart and adjusting calories/volumes to maintain the trend
- Serum electrolytes (assessed during study visits and, if needed for PN adjustment, will also be assessed in between visits)
- Increase in the Volume Intake/Output ratio, including mixed output that can be collected
- Consideration of clinical deterioration in SBS
- Weight loss
- Diarrhea (> 50 mL/kg/day with ostomy)
- Vomiting frequency
- Fluid balance
- Skin breakdown of perianal area

Adjustments will be based on the actual nutritional support in volume and calories the subject infuses. Subjects are expected to remain compliant with the nutritional support prescription in volume and calories during the study. Nutritional support constituents may be adjusted at the discretion of the investigator. During the 48-hour Intake/Output measurement period, no significant changes to nutritional support will be made. If there is a change in oral fluid intake, the investigator will consider this when adjusting the nutritional support in volume and calories.

## STEPS FOR ADJUSTING NUTRITIONAL SUPPORT VOLUME AND CALORIES (Figure 2)

1. Subjects will be assessed during weekly telephone visits and at each study visit for hydration and nutrition. A diary will be kept by the caregiver. The caregiver will make all measurements prior and after the scheduled study visits.
2. Nutritional Support changes will be made in accordance with Figure 2. Additional considerations include the following:

- Consideration for nutritional support reduction and advancement of enteral feeds will be in the context of maintaining clinical status and the opportunity to gain additional days off per week of infusions or be completely weaned off nutritional support.
- Reductions in nutritional support will be considered in terms of calories and/or volume per week, hours per day, and complete days off.
- This adjustment will be done under the supervision of and consultation with the investigators.

Figure 2. Nutritional support adjustments based on calories


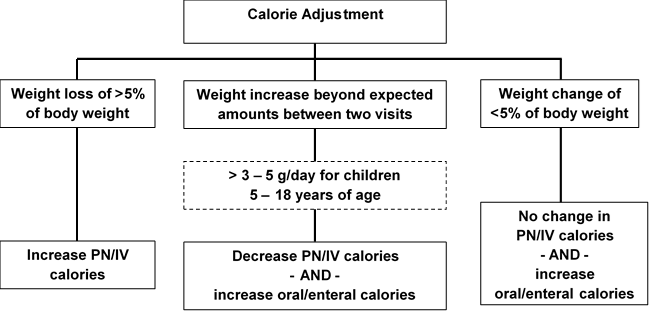


# EXPERIMENTAL PROCEDURES

## OVERVIEW: SCHEDULE OF TIME AND EVENTS

This clinical trial includes an initial screening visit in which subjects will be screened for eligibility. At screening the investigator or designee will explain the purpose and evaluations of the study to the subject’s parent or legal guardian, and obtain informed consent (and assent, if applicable). If consent is given and based on the results of the screening assessments, the subject will be enrolled in the study. Parents or guardians will receive education and support from a study dietician to assist them in keeping track of daily caloric intake and composition (fat, carbohydrates, protein) in oral and enteral feeds, and TPN. As part of this education, the subject’s parent or guardian will receive access to a nutrition/food tracking phone application (Cronometer) to assist them with these tasks throughout the study. A registered nurse will review how to perform accurate stool collection and subjects will be provided with a stool container in the event they are eligible.

If enrolled based on the results of screening, the subject and legal guardian will then be required to perform a 72-hour stool collection and caloric intake recording during the enrollment period (Days -3 to -1) of the study. The subject will return to the clinic on Day 1. At this clinic visit, any baseline assessments not obtained during the enrollment period will be performed, and the first treatment will be administered. Baseline assessments will include physical examination, nutritional assessment, vital signs, clinical evaluations and blood collection for lipid/FA profiles. Physical examinations performed during the 3-day enrollment period will be used for the baseline assessment when available. The subject’s nutritional intake (enteral and oral intake, and PN volume) and stool consistency/amount/frequency (if applicable) or ostomy output (if applicable) will be assessed. At this and each subsequent visit, parents or guardians will continue to receive nutrition education and support from a study dietician.

During the treatment period, all enteral nutrition will be administered using the RELiZORB enzyme cartridge. Nutritional intake and composition (24 hour oral and enteral intake and PN volume) will be recorded on the phone based application (Cronometer; or paper). Stool consistency (assessed through the Bristol scale), stool amount/frequency (if applicable) or ostomy output (if applicable), study device use, and incidence of any changes in symptoms (e.g., abdominal pain, increases in stool output above baseline, change in urine color/frequency) will be recorded by the subject’s parent or guardian in their electronic or paper diary. After the daily phone call with a study coordinator, the coordinator will email the study team list serve to address any issues or concerns as these occur. Advancement of oral or enteral feedings and weaning from PN feeding will be determined by the investigator weekly and at each clinic visit based on the subject’s clinical status, which will include assessment of weight, growth and hydration status. Assessments for the study endpoints and safety will be collected at clinic visits, by physical examination, clinical evaluations and review of the subject’s diary. Assessments will also occur at interim weekly telephone visits after the Day 14 clinic visit as above.

A complete overview of the time and events schedule of this study is presented in Table 2.

Table 2. Schedule of Time and Events

|  | **Study Period** | | | | | | | | |
| --- | --- | --- | --- | --- | --- | --- | --- | --- | --- |
|  |  | | **Treatment Period** | | | | | | |
| **Measurements/Evaluations** | **Screen-**  **ing**  **Day -3** | **Enroll-ment**  **Days**  **-3 to -1** | **Baseline and first treatment**  **Day 1** | **Day**  **2-6** | **Day 7±3** | **Day**  **8-13** | **Day 14±3** | **Day**  **15-27**  **Day**  **29-59**  **Day**  **61-89** | **Day**  **28±3**  **Day**  **60±3**  **Day**  **90±3** |
| I/E criteria | X |  |  |  |  |  |  |  |  |
| Informed consent | X |  |  |  |  |  |  |  |  |
| Demographics | X |  |  |  |  |  |  |  |  |
| Bowel length^a^ | X |  |  |  |  |  |  |  |  |
| Standard of care | X | Daily | X | Daily | X | Daily | X | Daily | X |
| Medical history | X |  |  |  |  |  |  |  |  |
| Vital signs^b^ | X |  | X |  | X |  | X |  | X |
| Anthropometry/Physical exam^c^ | X |  | X |  | X |  | X |  | X |
| Study Coordinator Call |  | Daily |  | Daily |  | Daily |  | Daily |  |
| Clinical laboratory evaluations^d^ | X |  |  |  | X |  | X |  | X |
| Dietician Education | X |  | X |  | X |  | X |  | X |
| 72-hour fecal collection^e^ |  | X |  |  |  |  |  |  | X (final visit) |
| Lipid profile^f^ | X |  |  |  | X |  | X |  | X |
| Total plasma FAs^g^ | X |  |  |  | X |  | X |  | X |
| Liver tests^h^ | X |  |  |  | X |  | X |  | X |
| Nutritional assessment | X |  | X |  | X |  | X | Weekly | X |
| Record nutritional intake^i^ |  | Daily | X | Daily | X | Daily | X | Daily | X |
| Change in PN/ enteral feeding | X | X | X |  | X |  | X | Weekly | X |
| Home Body weight (WAZ) |  |  |  |  |  |  |  | Weekly |  |
| RELiZORB |  |  | X | Daily | X | Daily | X | Daily | X |
| Adverse Events^j^ |  |  | X | Daily | X | Daily | X | Daily | X |
| Concomitant Medications |  |  | X | Daily | X | Daily | X | Daily | X |
| Assess medical/ surgical procedures | X |  | X |  |  |  | X |  | X |
| Supply Investigational Product |  |  | X |  | X |  | X |  | X |
| Review Daily Patient Diary |  |  |  |  | X |  | X | Weekly | X |

I/E criteria = Inclusion and exclusion criteria.

FAs =fatty acids.

PN = Parenteral nutrition.

^a^ Record known bowel length/type from last surgical resection or other measurement.

^b^ Blood pressure, pulse rate, respiratory rate and temperature.

^c^ Includes height or length, head circumference (for subjects < 36 months), and weight. If a physical examination or nutrition assessment was performed during the 3-day screening period, it will be used for the baseline assessment when available.

^d^ Includes chemistry (including fat-soluble Vitamins A, D, E, and K), hematology, coagulation and C-reactive protein (CRP). Clinical laboratory evaluations obtained at time of screening will serve as the baseline assessment.

^e^ 72-hour fecal collection will occur in the 72 hours preceding treatment day 1 and the final clinic visit day 90

^f^ Samples of the lipid profile will include cholesterol, high-density lipoprotein (HDL), very low-density lipoprotein (VLDL), and low-density lipoprotein (LDL). Results of samples obtained at time of screening will be analyzed during enrollment period, prior to participant receiving device.

^g^ Samples of total plasma fatty acids will include total saturated, total monosaturated, total polyunsaturated, total Omega-3, total Omega 6, total fatty acids, a-linolenic Acid, linoleic acid, oleic acid, EPA, arachidonic acid, mead acid, DHA, and triene:tetraene ratio.

^h^ Serum alanine aminotransferase (ALT), serum aspartate aminotransferase (AST), and serum gamma-glutamyl transpeptidase (GGT)

^i^ 24-hour nutritional intake/composition (PO and enteral dietary intake and PN volume) will be recorded using a phone-based application or paper diary. This will be especially important during the 72-hour stool collection periods for calculation of the coefficient of fat absorption.

^j^ Adverse events will be recorded daily in an electronic or paper format by the subject’s parent or guardian, and by study staff during clinic visits. Monitoring of these events will also occur through a daily phone call from a study coordinator. If any AEs or concerns are noted by the family, the study coordinator will alert the study team by email. PI or other delegated investigator will determine next steps

# MEASUREMENTS AND EVALUATIONS

### Screening Period (Day -3)

Before the initiation of screening assessments, the subject and/or subject’s parent/guardian must be given a complete explanation of the purpose and evaluations of the study. Subsequently, the subject and/or subject’s parent or guardian must sign and receive a copy of an informed consent form and an authorization for use and disclosure of protected health information that was approved by the institutional review board or independent ethics committee. Once informed consent and authorization for use and disclosure of protected health information have been obtained, the screening assessments will be performed, and the eligibility of the subject will be determined*.*

The following evaluations will be performed to assess the subject’s eligibility for the study:

- Complete medical history, including bowel length from last surgical resection and any other medical/surgical procedures done.
- Vital signs
  - Temperature, blood pressure, pulse rate, and respiratory rate
- Overview with study participant and parent/guardian of methods of stool collection and storage to ensure compliance
- Clinical lab evaluations including:
  - Chemistry (sodium, potassium, chloride, CO2, glucose, blood urea nitrogen (BUN), creatinine, calcium, phosphorus, magnesium, alkaline phosphatase, albumin, pre-albumin, total protein, total and direct bilirubin
  - Hematology
  - Coagulation
  - Fat-soluble Vitamins A, D, E and K
  - Serum C-reactive protein (CRP), fatty acid profile
- Lipid Profile including:
- Triglycerides, cholesterol, high-density lipoprotein (HDL), very low-density lipoprotein (VLDL), low-density lipoprotein (LDL))
- Total plasma Fatty Acids including:
- Total saturated, total monosaturated, total polyunsaturated, total Omega-3, total Omega 6, total fatty acids, a-linolenic Acid, linoleic acid, oleic acid, EPA, arachidonic acid, mead acid, DHA, and triene:tetraene ratio
- Liver tests including:
- Serum alanine aminotransferase (ALT), Serum aspartate aminotransferase (AST), Serum gamma-glutamyl transpeptidase (GGT)
- Nutritional intake
  - Oral and enteral intake and PN volume intake (total calories and composition in terms of fat, carbohydrate, proteins)
  - Stool consistency (Bristol scale)/amount/frequency (if applicable)
  - Ostomy (if applicable) output

### Enrollment Period (Day -3 to -1)

After completion of screening and if eligible, the subject will be enrolled in the study. During this enrollment period, the subject’s parent or guardian will be responsible for a 72-hour stool collection preceding Day 1. If necessary (for example, out of state participants, excessively watery stools), subjects will be admitted to a dedicated research center at our institution during this collection period. This stool collection will be repeated prior to the final Day of the study (Day 90).

### First Day of Treatment (Day 1)

Baseline evaluations will be performed for assessment of safety and clinical endpoints.

Assessments of safety will include:

- Vital signs
  - Temperature, blood pressure, pulse rate, and respiratory rate
- Physical examination
  - Height or length, head circumference (for subjects < 36 months), and weight.
  - Physical examinations performed during the 3-day screening period will be used for baseline assessment when available

• Concomitant Medications

• Medical/ surgical procedures

**Assessments of clinical endpoints will include:**

- Review of results of 72-hour stool collection that occurred during enrollment period as noted in section 7.1.2:
  - Stool total fat and fatty acid measurements
  - Measurements of dietary (oral/enteral) and PN intake of fat and during the 72-hour collection
- Review of lipid profile results from samples collected at screening visit as noted in section 7.1.1.
  - Cholesterol, high-density lipoprotein (HDL), very low-density lipoprotein (VLDL), low-density lipoprotein (LDL), triglycerides.
  - The lipid profile will be obtained at time of screening but will not be analyzed until patient has been enrolled into the protocol.
- Review of fat-soluble vitamins in plasma samples (Vitamin A, D, E, and K) results from samples collected at screening visit as noted in section 7.1.1.
- Nutritional intake
  - Oral and enteral intake and PN volume intake will be documented in the electronic phone application (Cronometer) or paper diary and will be available to study staff in real time.
  - Stool amount/frequency or ostomy (if applicable) output

On study Day 1, subjects will receive their enteral nutrition through the RELiZORB enzyme cartridge.

The subject’s parent or guardian will be given access to a phone-based nutrition tracking application (Cronometer) for recording nutritional intake (oral and enteral intake and PN volume). The subject’s parent or guardian will also have access to an electronic diary (or paper if no electronic capabilities) and instructed to record any changes in symptoms [e.g., abdominal pain, changes in stool consistency based on the Bristol scale, increases in stool output above baseline, stool amount/frequency (if applicable) or ostomy (if applicable) output, changes in urine color (through dehydration color scale) and frequency and administration of the study device]. The subject’s parent or guardian will be instructed how to store and administer the study device and will be provided with the study device to take home.

The first time the device is used to administer enteral nutrition will be done so under the supervision of a member of the research team; the patient’s response will be monitored at clinic. Investigational product will be supplied at each clinic visit. The feeding regimen, including type of formula, will not be changed for research purposes. Subjects will remain on their current regimen. Additionally, only standard (non-blenderized) formulas will be permitted to be used with the RELiZORB enzyme cartridge. Amino-acid based formulas that are compatible with the RELiZORB enzyme cartridge will be permitted.

### Treatment Period (Day 1 to 90)

During the treatment period (Day 1 to Day 90) the study device will be used daily with the administration of enteral nutrition.

At each visit and during weekly phone calls, the investigator will assess the subject’s nutritional intake, weight, growth and hydration status to determine if, based on their medical judgment, changes to the subject’s nutritional treatment should be made.

The subject’s parent or guardian will record the use of the study device, the subject’s nutritional intake (24 hour oral and enteral intake and PN volume), stool amount/frequency or ostomy (if applicable) output, and any changes in symptoms (e.g., abdominal pain, increases in stool output above baseline using the diary). In addition, if the subject experiences any unexpected physical or medical condition, the subject’s parent or guardian will be directed to call the clinic immediately and report it to the study staff.

Subjects will return to the clinic on Day 7, Day 14, Day 28, Day 60, and Day 90 of the treatment period. Site staff will review the information in the subject diary, review study device compliance, and discuss findings with the subject and subject’s parent or guardian during the visit. A study dietician will visit with the subject and subject’s parent or guardian to review nutrition information and address any issues or concerns.

At each clinic visit, vital signs, physical examination, adverse event assessment, concomitant medication review and review of any medical/ surgical procedures, clinical evaluations and liver enzymes will be performed to assess safety. Blood samples will be collected to analyze lipid profiles. Prior to the final visit, 72-hour fecal samples will be collected to analyze fecal composition. Any AEs that occur will be recorded in the CRF.

A daily study coordinator phone call will be done throughout the study (Day 1 to Day 90). The study dietician and investigators will have telephone contact with families during interim weeks after the day 14 visit. Study staff will monitor safety, diaries, and any changes in nutritional support. If necessary, unscheduled visits can be arranged in place of the telephone contacts.

The investigator will assess the subject’s nutritional treatment, weight, growth and hydration status to determine if, based on their medical judgment, advancement of oral or enteral feeding and weaning from PN should occur. This will occur during study visits as well as through the weekly telephone contact with families. Families will be provided with a standardized weight scale at the time of inclusion of the study for consistent weight measurements.

Any AEs that occur after the start of use of the study device will be recorded according to the procedures in Section 9.0[_RECORDING_ADVERSE_EVENTS](#_RECORDING_ADVERSE_EVENTS). Any significant clinical finding prior to administration of the study device will be included in the subject’s medical history.

### Standard of Care

All subjects will receive standard medical, surgical, and nutritional care during the study. Standard nutritional care will be provided as follows:

- All subjects without existing central venous catheters will undergo placement of these catheters for intravenous fluid management and administration of hypertonic nutrition solutions.
- PN will be increased when oral energy intake declines to less than baseline resting energy requirements for more than 3 days or the patient’s underlying clinical condition is such that PN should be initiated immediately so as to maintain protein stores.
- Maintenance fluid requirements for all subjects will be calculated according to the Holliday-Segar Method, detailed in Table 3.
- Amino acids will be provided according to standard, age-adjusted estimations for subjects receiving parenteral nutrition, detailed in Table 4.
- The nutritional regiment for subjects is provided in Table 5.

Table 3. Daily Fluid Requirements by Body Weight, According to the Holliday-Segar Method

| **Daily Fluid Requirements** | |
| --- | --- |
| **Body weight** | **Maintenance Parenteral Fluid Requirements** |
| 0 to 10kg | 100 mL / kg |
| 10 - 20 kg | 1000 mL + 50 mL / kg over 10 |
| Over 20 kg | 1500 mL + 20 mL / kg over 20 |

Table 4. Daily Caloric and Protein Requirements

| **Daily Caloric and Protein Requirements** | | |
| --- | --- | --- |
| **Age** | **kcal/kg/day** | **Protein g/kg/day** |
| 1 to 7 years | 75 to 90 | 1.5 to 3 |
| 7 to 10 years | 50 to 75 | 1.5 to 3 |
| 11 to 12 years | 50 to 75 | 0.8 to 2.5 |
| 13 to 18 years | 30 to 50 | 0.8 to 2.5 |

Table 5. Nutritional Regimen

| Dietary Component | Dose |
| --- | --- |
| Fat (enteral) | No more than 40% of total calories (PN + EN) |
| Electrolytes | As per clinical needs |
| Vitamins | >3 kg and <11 years of age: 5 ml/day MVI Pediatric^®^  >11 years of age: 1 vial MVI 13 (contains 150ug vitamin K) |
| Trace elements | As per clinical needs |

[**7.1.6**](#_MEASUREMENTS_AND_EVALUATIONS)  **Analysis of fecal fat and fatty acids profiles**

Prior to receiving the study device, a 72-hour stool collection will be obtained during the enrollment period for fecal fat and fatty acid analysis. This process will be repeated prior to the final study visits. Throughout the study, plasma samples will also be collected for fatty acid analysis.

*Protocol:*

1. Collected stool is homogenized, weighed and a representative sample is taken.
2. *Quantitative total fat*: The collected stool sample is sent to BCH central lab for quantitative total fecal fat measurement
   1. The coefficient of fat absorption will be calculated using the total stool mass, total fat analysis, and the following equation:

CFA= [Total Dietary fat (72 hours) – Total Stool Fat (72 hours)] ÷ Total Dietary Fat (72 hours)

1. *Stool and Plasma Fatty acid profile* :

Stool and plasma fatty acid composition will be analyzed at OmegaQuant Analytics by gas chromatography (GC) with flame ionization detection. Stool samples will be weighed into a screw-cap glass vial which contain tritricosanoin as an internal standard (tri-C23:0 TG) (NuCheck Prep, Elysian, MN), homogenized and then extracted with a modified Folch extraction. A portion of the organic layer will be transferred to a screw-cap glass vial and dried in a speed vac. After samples are dried BTM (methanol containing 14% boron trifluoride, toluene, methanol; 35:30:35 v/v/v) (Sigma-Aldrich, St. Louis, MO) is added. The vial is briefly vortexed and heated in a hot bath at 100˚C for 45 minutes. After cooling, hexane (EMD Chemicals, USA) and HPLC grade water is added, the tubes are recapped, vortexed and centrifuged help to separate layers. An aliquot of the hexane layer will be transferred to a GC vial. GC will be carried out using a GC-2010 Gas Chromatograph (Shimadzu Corporation, Columbia, MD) equipped with a SP-2560, 100-m fused silica capillary column (0.25 mm internal diameter, 0.2 um film thickness; Supelco, Bellefonte, PA).

1. Fatty acids are identified by comparison with a standard mixture of fatty acids (GLC OQ-A, NuCheck Prep, Elysian, MN) which was also used to determine individual fatty acid calibration curves. Fatty acid composition is expressed as a percent of total identified fatty acids and concentrations as µg/mg of stool.

# DATA AND SAFETY MONITORING BOARD (DSMB)

The Data Safety Monitoring Board (DSMB) will be an independent board comprised of three physicians and a biostatistician not participating in this study. Considering this being an open label study committee, membership will include three independent clinicians who are expert in the field, and one biostatistician. One of the clinicians will be appointed as chairman of the DSMB. The DSMB is responsible for safeguarding the interests of study participants, assessing the safety and efficacy of study procedures, and for monitoring the overall conduct of the study. Specific details about the DSMB composition, responsibilities, organization and meeting frequency can be found in the DSMB Charter.

# PROCEDURES FOR HANDLING ADVERSE EVENTS AND SERIOUS ADVERSE EVENTS

## DEFINITION OF AN ADVERSE EVENT

The following definition of adverse event (AE) will be used for this study:

- Adverse event means any untoward medical occurrence associated with the use of a device in humans, whether or not considered device-related.
- All AE’s post-baseline will be captured. Adverse events grade 2 or higher will be analyzed.

An adverse event can be any unfavorable and unintended sign (including an abnormal laboratory finding), symptom, or disease (new or exacerbated) temporally associated with the use of the investigational product, regardless of whether it is considered to be related to the investigational product.

Examples of AEs include the following:

- Significant or unexpected worsening or exacerbation of the indication under study
- Exacerbation of a chronic or intermittent preexisting condition including an increase in frequency or intensity of the condition
- New conditions detected or diagnosed after investigational product administration even if they were present before the start of the study
- Signs, symptoms, or the clinical sequelae of a suspected interaction with another medical product
- Signs, symptoms or the clinical sequelae of a suspected overdose of either investigational product or a concurrent medication

Overdose will not be reported as an AE or serious adverse event (SAE), but rather the symptoms resulting from the overdose.

Examples of AEs do not include the following:

- Situations that are unwanted by the subject but in which an untoward medical occurrence did not occur, for example social inconvenience after admission to a hospital
- Anticipated day-to-day fluctuations of a preexisting disease or condition (present or detected before enrollment) that does not worsen overall
- Expected progression of the disease being studied, including signs or symptoms of the disease, unless progression is more severe than expected for the subject’s condition
- The Common Terminology Criteria for Adverse Events (CTCAE) version 5 (https://ctep.cancer.gov/protocoldevelopment/electronic_applications/docs/CTCAE_v5_Quick_Reference_5x7.pdf) will be used to grade toxicities.

The investigator is responsible for performing periodic and special assessments for AEs. The investigator and study personnel will note all AEs mentioned by the subject after administration of the investigational product. Any significant clinical finding detected prior to administration of the study device will be included in the subject’s medical history. All clinical complaints volunteered by or elicited from the subject during the study will be recorded on the appropriate page of the CRF for the study period indicated. The subject will receive appropriate treatment and medical supervision for any AE that occurs. Subjects with anaphylactic or allergic reactions will not continue to receive the study device.

All AEs judged to be clinically significant, including clinically significant laboratory abnormalities, will be followed until resolution. All AEs will be summarized in the annual report or more frequently if requested by the regulatory agency. SAEs require special reporting in addition to documentation in the CRF as described in Section 9.8.

## DEFINITION OF A SERIOUS ADVERSE EVENT

In this clinical trial, a serious adverse event is defined as an AE that meets any of the following criteria:

- Results in death
- Is life-threatening

The term life-threatening in the definition of an SAE refers to an event in which the subject was at risk of death at the time of the event. Life‑threatening does not refer to an event that hypothetically might have caused death if it were more severe.

- Requires hospitalization or a prolongation of an existing hospitalization

In general, hospitalization signifies that the subject has been detained at the hospital or emergency ward for observation or treatment that would not have been appropriate in the physician’s office or out-patient setting. Complications that occur during hospitalization are AEs, but not necessarily SAEs. An occurrence or complication that prolongs hospitalization is an SAE. When there is doubt as to whether hospitalization occurred or was necessary, the AE will be considered an SAE. Hospitalization for elective treatments of a preexisting condition that did not worsen from its original baseline level is not considered an SAE.

- A persistent or significant incapacity or substantial disruption of the ability to conduct normal life functions

This definition is not intended to include AEs of relatively minor medical significance such as uncomplicated headache, nausea, vomiting, diarrhea that may interfere or prevent everyday life functions but do not constitute a substantial disruption.

- Other important medical event

Medical or scientific judgment will be exercised in deciding whether reporting is appropriate for other important medical events that may not result in death, be life-threatening, or require hospitalization but still may jeopardize the subject or may require medical intervention to prevent one of the outcomes listed in this definition. These events will also be considered serious. Examples of such events are intensive treatment in an emergency room or at home for allergic bronchospasm, blood dyscrasias or convulsions that do not result in hospitalization, or development of drug dependency or drug abuse.

An SAE requires additional detailed reports and follow-up. The content of these detailed reports must address the investigator’s estimate of causality. The principal investigator will review the SAE to determine if it is an expected SAE (i.e., whether or not the SAE is identified in nature, severity, and frequency in the Investigator’s Brochure).

## DEFINITION OF UNANTICIPATED ADVERSE DEVICE EFFECT

Per 21 CFR 812.3(s), an unanticipated adverse device effect is defined as “any serious adverse effect on health or safety or any life-threatening problem or death caused by, or associated with, a device, if that effect, problem, or death was not previously identified in nature, severity, or degree of incidence in the investigational plan or application (including a supplementary plan or application), or any other unanticipated serious problem associated with a device that relates to the rights, safety, or welfare of subjects.”

## RECORDING ADVERSE EVENTS AND SERIOUS ADVERSE EVENTS

When an AE, SAE, or UADE occurs, the investigator will be responsible for reviewing all documentation (e.g., hospital progress notes, laboratory, and diagnostic reports) relative to the event(s). The investigator will record all relevant information about any AE (including SAEs and UADEs) on the AE log. It is not acceptable for the investigator to send photocopies of the subject’s medical records in lieu of the properly completed AE or SAE log. If this request occurs, all subject identifiers and protected health information will be blinded on the copies of the medical records, before submission to the appropriate authorities.

The investigator will also attempt to report a diagnosis versus signs, symptoms, or other clinical information for the AE. The diagnosis, not the individual signs and symptoms, will be documented on the appropriate page of the AE or SAE log. In addition, SAEs need to be reported on the SAE report form.

## ASSESSMENT OF INTENSITY

The investigator will assess the intensity for each AE and SAE reported during the study. The assessment will be based on the investigator’s clinical judgment.

The classifications in Table 6 will be used in assigning intensity of each AE recorded in the case report form. The Common Terminology Criteria for Adverse Events (CTCAE) version 5.0 will be used to grade toxicities. (https://ctep.cancer.gov/protocolDevelopment/electronic_applications/ctc.htm)

Table 6. Classification of AEs by Intensity

| **Intensity** ^a^ | **Definition** |
| --- | --- |
| Mild AE (Grade 1) | An event that is easily tolerated by the subject, causing minimal discomfort and not interfering with everyday activities. |
| Moderate AE (Grade 2) | An event that is sufficiently discomforting to the extent of interfering with normal everyday activities. |
| Severe AE (Grade 3) | An event that prevents the subject from performing normal everyday activities. |
| Life-threatening or disabling AE (Grade 4) | An event that, at the time of occurrence, put the subject at risk of death or resulted in a persistent or significant disability or incapacity |
| Death related to AE  (Grade 5) | An event that resulted in death |

AE = adverse event

a From Common Terminology Criteria for Adverse Events (https://ctep.cancer.gov/protocolDevelopment/electronic_applications/ctc.htm)

Any AE that changes in intensity or grade during its course will be recorded in the CRF at the highest-level experience by the subject during a single course.

An AE that is assessed as severe should not be confused with an SAE. Severity is a category used for rating the intensity of an AE (such as mild, moderate, or severe myocardial infarction). However, the event itself may be of relatively minor medical significance, such as a severe headache. Both AEs and SAEs can be assessed as severe. An AE is defined as serious when it meets one of the pre‑defined outcomes as described in Section 9.2.

## ASSESSMENT OF CAUSALITY

The investigator must estimate the relationship between the investigational product and the occurrence of each AE or SAE by using his or her best clinical judgment. Elements to consider for this estimate include the history of the underlying disease, concomitant therapy, other risk factors, and the temporal relationship of the event to the investigational product. Because of reporting timelines, the investigator might have minimal information to include in the initial SAE report. However, the investigator must always make an assessment of causality for every event before the transmission of the SAE report. The investigator may change his or her opinion of the causality in light of follow-up information, with subsequent amendment of the SAE report. Causality assessment is one of the criteria used to determine regulatory reporting requirements and should not be left blank. Table 7 provides some definitions to use in the assessment.

Table 7. Assessment of Causality of AEs

| **Term** | **Definition** |
| --- | --- |
| **Definitely related** | The AE is clearly related to the investigational agent(s) or research intervention: the AE has a temporal relationship to the administration of the investigational agent(s) or research intervention and follows a known pattern of response, and no alternative cause is present. |
| **Possibly related** | The AE may be related to the investigational agent(s) or intervention: the AE has a temporal relationship to the administration of the investigational agent(s) or research intervention and follows a suspected pattern of response, but an alternative cause is present. |
| **Probably related** | The AE is likely related to the investigational agent(s) or intervention: the AE has a temporal relationship to the administration of the investigational agent(s) or research intervention and follows a known or suspected pattern of response, but an alternative cause may be present. |
| **Unrelated**  **(or Not Related)** | The AE is clearly not related to the investigational agent(s) or intervention: the AE has no temporal relationship to the administration of the investigational agent(s) or research intervention, and follows no known or suspected pattern of response, and an alternative cause is present. |

AE = adverse event.

## EXPECTEDNESS OF SERIOUS ADVERSE EVENTS

An expected AE is one that is consistent with the known risk information described in the product label (if applicable) or the current Investigator’s Brochure. The assessment of the expectedness of an SAE will be done by the Principal Investigator, upon receipt of the initial SAE report.

## REPORTING OF SERIOUS ADVERSE EVENTS

Any SAE occurring after use of the study device must be reported to the IRB and the Data and Safety Monitoring Board (DSMB) by phone or in person within 72 hours of the time the investigator becomes aware of the SAE, or within 24 hours if the event is fatal or life threatening. Urgent reporting of SAEs is required for the following reasons:

1. To enable the Investigational Product developer/distributer to fulfill the reporting requirements to the appropriate regulatory authority
2. To facilitate discussion between the Investigational Product developer/distributer and the sponsor-investigator about appropriate follow-up measures (if necessary)
3. To facilitate reporting unanticipated problems involving risk to subjects to the institutional review board (IRB) or independent ethics committee (IEC)

The SAE report form will be completed as thoroughly as possible, including:

- Subject identification information
- All available details about the event
- Causality of each SAE
- Signature of the investigator

The SAE report form will be forwarded to the safety department within the designated time frames. If additional information to complete the SAE report form is needed, the investigator will not wait before notifying the safety department of the SAE. The SAE report form will be updated by the investigator when additional information is received.

SAEs and UADEs will be reported to the FDA in accordance with FDA reporting requirements.

## FOLLOW-UP OF ADVERSE EVENTS AND SERIOUS ADVERSE EVENTS

After the initial AE or SAE report, the investigator is required to proactively follow each subject and provide further information to the safety group about the subject’s condition.

All AEs and SAEs will be followed until the occurrence of one of the following:

- Resolution
- The condition stabilizes
- The event is otherwise explained
- The subject is lost to follow-up

The appropriate SAE report form will be updated once the SAE resolves, stabilizes, is otherwise explained, or the subject is lost to follow-up. The investigator will also ensure that updates include any supplemental data that may explain causality of the event(s).

New or updated information will be recorded on a copy of the initial SAE report form, with all the changes signed and dated by the investigator or designee. The updated SAE report form will then be signed by the investigator and resubmitted to the safety department.

## LIVER EVENTS

When either serum alanine aminotransferase (ALT) or serum aspartate aminotransferase (AST) levels exceed three times higher than values recorded at screening, serum ALT, AST, alkaline phosphatase, and total bilirubin will be repeated within 48 to 72 hours. Alcresta will be notified, and liver event pages of the case report form will be completed. If the ALT or AST remain more than three times the values recorded at screening, or total bilirubin remains greater than two times ULN, then repeat liver enzymes will be obtained every 3 days and the appropriate case report form page will be completed with available history of symptoms and other assessments (e.g., ultrasound or computerized tomography of the liver and tests for hepatitis).

Investigational product use will stop if one of the following occurs:

- ALT or AST exceed eight times the values recorded at screening.
- ALT or AST exceed five times the values recorded at screening for more than 2 weeks.
- ALT or AST exceed three times the values recorded at screening and total bilirubin exceeds two times ULN or international normalized ratio exceeds 1.5.
- ALT or AST exceed three times the values recorded at screening, with the appearance of fatigue, nausea, vomiting, right upper quadrant pain or tenderness, fever, rash, or eosinophilia (>5%).
- Monitoring will be continued until abnormalities return to reference range or baseline levels.

## TREATMENT INTERRUPTION

## Treatment interruption will be allowed for subjects who require temporary reduction or elimination of enteral nutrition due to intercurrent illness. The RELiZORB enzyme cartridge will continue to be used for any enteral nutrition that is administered during this time. Periods of intercurrent illness will be recorded on the adverse event form and addressed at the time of data analysis (see analysis section).

## SUBJECT DISCONTUNUATION

Subjects will be encouraged to complete the study; however, they may voluntarily withdraw at any time. The investigator will provide a written explanation of the reason for discontinuation in a source document, which will be transcribed to the appropriate CRF page. If a subject withdraws before completion, every effort will be made to complete the assessments scheduled during the final clinic visit.

A subject may be removed from the study for the reasons described in the sections below. Additionally, the subject will be removed from the study if any of the events described below (i.e., adverse event or intercurrent illness) result in a feed being withheld for more than 24 hours.

### Adverse Event

If a subject suffers an AE that, in the judgment of the investigator, the investigational product developer/distributer, or the DSMB presents an unacceptable consequence or risk to the subject, the subject may be discontinued from the study.

### Intercurrent Illness

A subject may be discontinued from the study if, in the judgment of the investigator, the subject develops an intercurrent illness or complication that is not consistent with the protocol requirements or that, in any way, justifies withdrawal from the study.

### Noncompliance

After the sponsor-investigator and study monitor consult (and Alcresta if appropriate), a subject may be discontinued from the study for the following administrative reasons:

- Failure to receive study device or treatment as mandated by the specific protocol.
- Failure to comply with protocol requirements
- Unauthorized, subject-initiated changes in dosing regimen

### Refusal of Investigational Product Administration

Any subject refusing clinical trial material for any reason will be discontinued from the study, and the reason(s) will be documented on the appropriate CRF. Reasonable efforts will be made to monitor the subject for AEs and to complete follow-up assessments after discontinuation. These efforts will be documented on the appropriate CRF page.

### Withdrawal of Consent

Any subject that withdraws consent for any reason at any time during the study will be discontinued from the study, and the reason(s) will be documented on the appropriate CRF.

## STOPPING RULES

If the sponsor-investigator, study monitor, or appropriate regulatory officials discover conditions due to reasons including but not limited to futility or risk(s) to the participants that arise during the study, the study should be paused or halted. This action may be taken after appropriate consultation among the sponsor-investigator, developer/distributer of the investigational product, , and DSMB. The trigger point for AE and study pause: Severity Level 3 and 4 AEs will be reviewed by PI and determined if it is worthy of study pause. Any change in stool that is higher than baseline will be discussed on a patient to patient basis.

## PREMATURE STUDY TERMINATION

If the sponsor-investigator, study monitor, or appropriate regulatory officials discover conditions arising during the study that indicate that the study should be halted, this action may be taken after appropriate consultation among the sponsor-investigator, developer/distributer of the investigational product, study monitor, and DSMB. Conditions that may warrant termination of the study include, but are not limited to, the following:

- The discovery of an unexpected, serious, or unacceptable risk to the subjects enrolled in the study
- A decision on the part of the developer/distributer of the investigational product to suspend or discontinue testing, evaluation, or development of the product.

A study may also warrant termination under the following conditions:

- Failure of the investigator to enroll subjects into the study at an acceptable rate
- Failure of the investigator to comply with pertinent regulations of appropriate regulatory authorities
- Submission of knowingly false information from the site to the Investigational Product developer/distributer, study monitor, or appropriate regulatory authority
- Insufficient adherence to protocol requirements

Study termination and follow-up will comply with the conditions set forth in the International Council on Harmonisation E6 Guideline for Good Clinical Practice, Sections 4.12, 4.13, 5.20, and 5.21.

# DATA COLLECTION AND PROCESSING AND STATISTICAL ANALYSIS

## DATA COLLECTION AND PROCESSING

Paper case report forms (CRFs) will be used to capture study assessments and data. The study coordinator or other delegated study personnel will enter data from source documents into the CRFs and then into the electronic database. The investigator will sign off on appropriate CRFs. Study documents related to consent, eligibility Unanticipated Adverse Device Effects (UADESs), and device accountability will be reviewed by the study monitor during periodic site visits. This can include consent forms, CRFs, study logs and source documents.

Training will be provided for the electronic data capture (EDC) system, InForm. All clinical trial personnel using the EDC system must have the necessary education, training, and experience or any combination of these. The investigator will be responsible for documenting employee education, training, and previous experience that pertain to the EDC system for all site personnel using the EDC system.

The investigator must maintain adequate security of the EDC system, including documentation that all users have been trained on the appropriate standard operating procedure and a list of authorized users. To ensure all data entries can be tracked, all personnel responsible for data entry must obtain InForm training certificates before any data can be entered in the database. The EDC system is configured to track all user entries and edits to the data.. Authorized study personnel will be assigned a unique password after receiving database training.

The sponsor-investigator must ensure that the electronic data systems are validated, and that data are backed up.

## STATISTICAL ANALYSIS

### General Overview

Data will be summarized in tables listing the number of subjects, mean, standard deviation, standard error, 95% confidence interval (CI), median, interquartile range (IQR), minimum, and maximum for continuous data and number of subjects, frequency and percentage for categorical data. Summaries will be presented by subject and visit, as well as in aggregate when appropriate. Outcomes will be presented in their native units (i.e. as collected), in addition to transformed units when necessary (for example logarithmic). Anthropometry outcomes (weight, height, head circumference, weight-for-height, body mass index) will be examined as z-scores using the Centers for Disease Control growth charts. All outcomes will be explored visually using various graphing methods, including (but not limited to) box-whisker, profile, and bar plots. All statistical analyses will be performed with SAS version 9.4 or later (Cary NC).

### Outcomes Evaluation

Continuous outcomes, which include all outcomes except the incidence of adverse events (AEs) and incidence of weaning from PN, will be described as indicated in the general overview above using summary measures at each visit. Incidence of ever weaning from PN will be summarized by frequency count and percentage. Analysis for incidence of AEs grade 2 or higher is described in the Safety Analysis section below.

Assessment of change in continuous outcomes will be made using the area under the curve between study day 7 and 90 (AUC_7-90_), adjusted for baseline, calculated over all relevant assessment timepoints (5 and 13 post-baseline weekly assessments for measurements obtained in clinic and at home, respectively). Measurements obtained in clinic will be assessed at study days 7, 14, 28, 60 and 90, while those obtained at home will be assessed on study days 7, 14, 21, 28, 35, 42, 49, 56, 63, 70, 77, 84, 90.

Adjustment for baseline will be made by dividing the outcome value at each assessment by the baseline value, leading to interpretation of AUC_7-90_ as a mean relative percent increase or decrease in outcome depending on whether AUC_7-90_ is greater or less than unity (1), respectively. AUC_7-90_ will be calculated as a time-weighted average, given by the equation:

$$\frac{0.5\cdot\sum\left( t_{i+1}-t_{i} \right)\left( r_{i}+r_{i+1} \right)}{\sum\left( t_{i+1}-t_{i} \right)}$$

where $t_{i}$ denotes the assessment day as described above,, and $r_{i}= y_{i}/y_{1}$ denotes the outcome at assessment day $t_{i}$ divided by the baseline (day 1) value. Hypothetical examples for a single subject with reduction in outcome at each visit is shown below in Figure 3 for measurements obtained in clinic (a) and measurements obtained at home (b). The dotted lines enclose a reference AUC_7-90_=1 (indicating no change in outcome), and the gray shaded area indicates AUC_7-90_.

Figure 3. Hypothetical examples of area under the curve for one subject

| 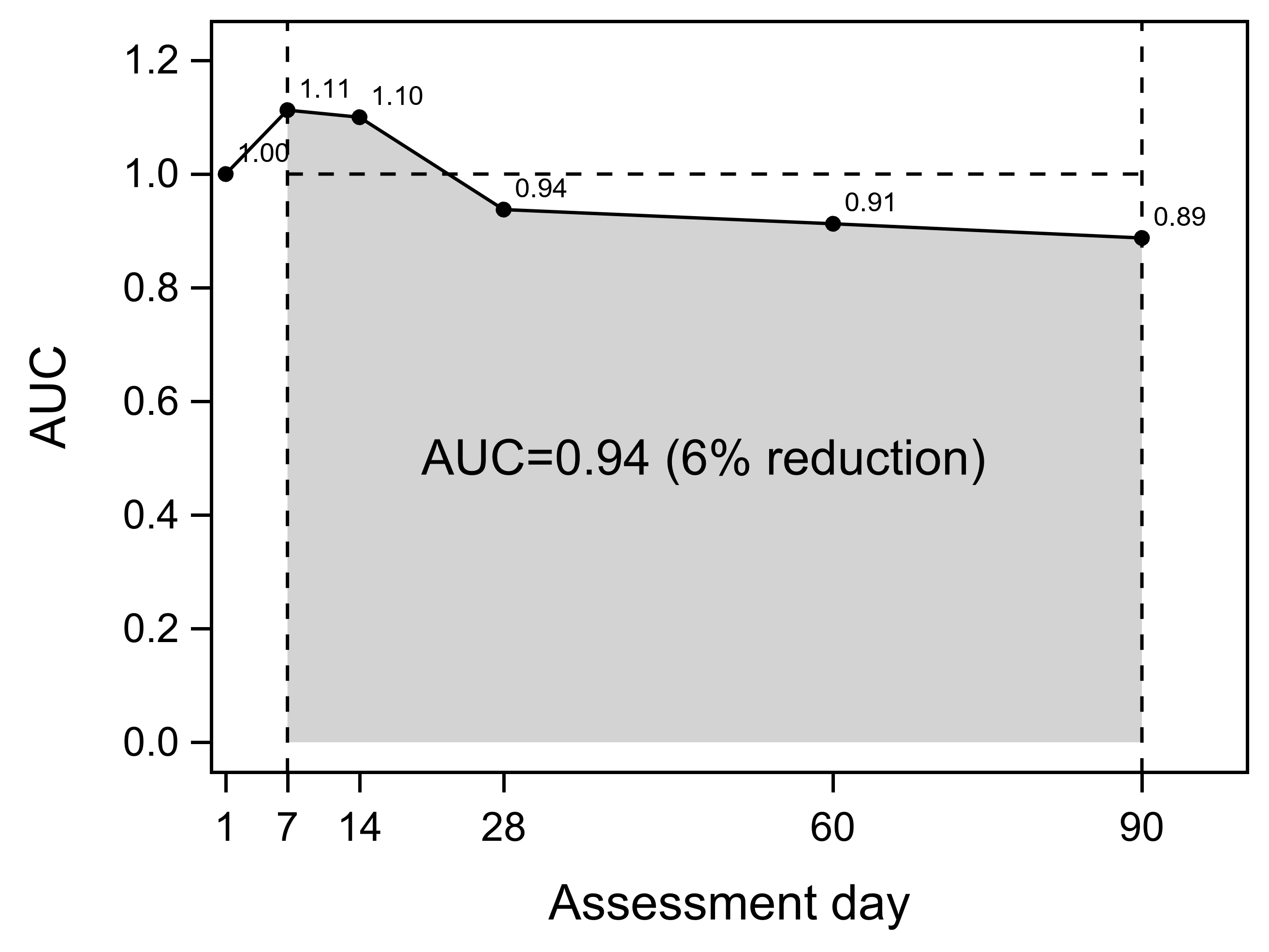 | 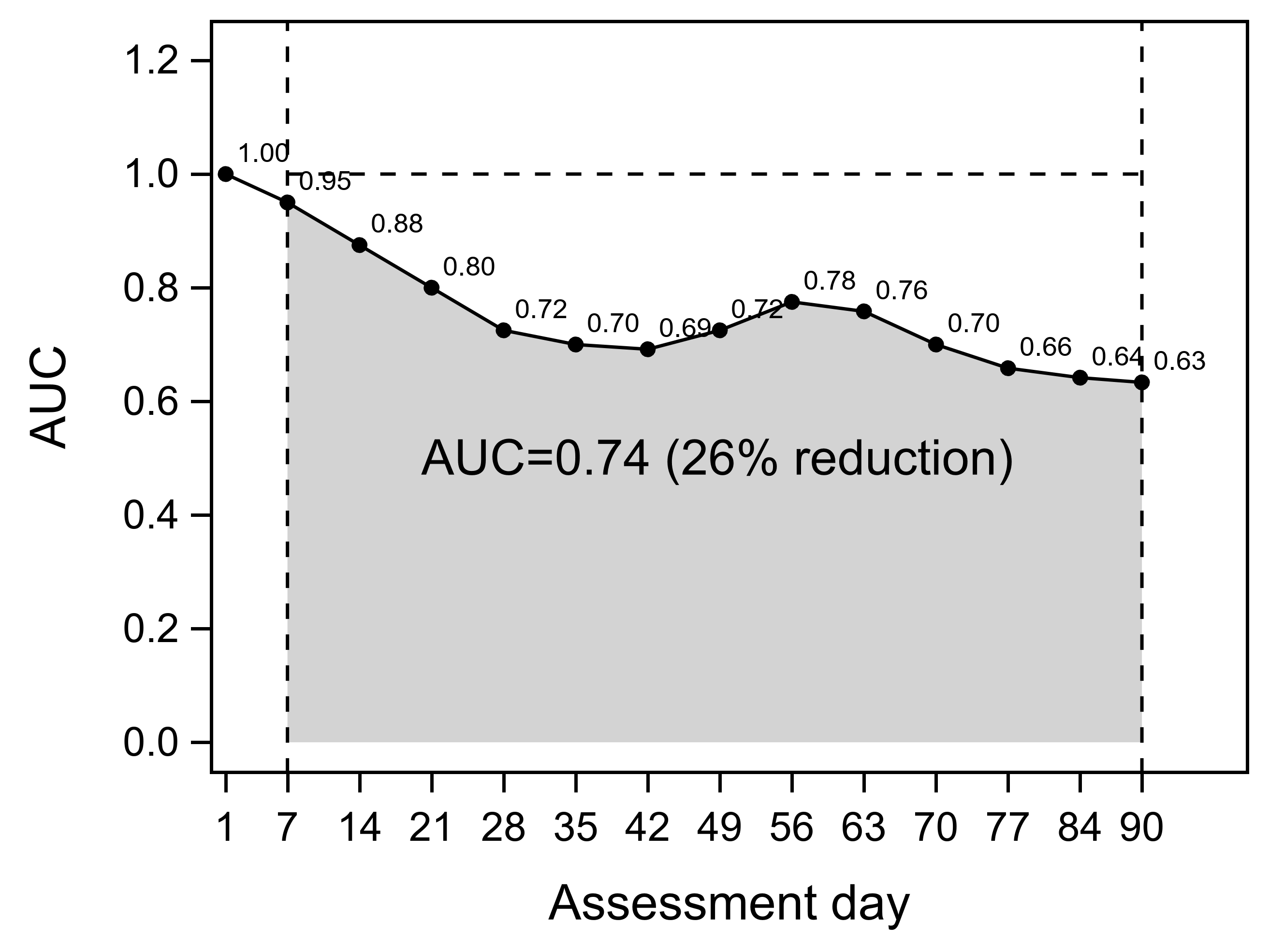 |
| --- | --- |
| a. Example of in-clinic outcome assessment. | b. Example of at-home outcome assessment. |

The assumption of normality will be assessed for AUC_7-90_ for each continuous outcome using quantile-quantile (Q-Q) plots and the Shapiro-Wilk test. Outcomes determined to be skewed will be normalized using an appropriate transformation (e.g. logarithm; normal scores based on ranks). AUC_7-90_ for each outcome will be summarized as described above in the General Overview section.

### Safety Analysis

Safety analysis will be performed on all subjects enrolled in the study with a follow-up safety assessment. Adverse event data will be listed individually and summarized by system organ class and preferred terms within a system organ class. The number and percentage of subjects with adverse events (AEs), serious adverse events (SAEs), AEs that lead to discontinuation, study device-related AEs (determined by investigator), and AEs that lead to death will be summarized. Only AEs grade 2 or higher will be presented. When calculating the incidence of adverse events, each adverse event will be counted only once for a given subject. If the same adverse event occurs on multiple occasions in a subject, the occurrence with the highest severity and relationship to study device will be reported. If two or more adverse events are reported as a unit, the individual terms will be reported as separate events. AEs will also be summarized with regards to severity and relation to study drug. Additionally, the frequency of each AE within-subject will be calculated and summarized across all subjects in order to rank the commonality of each particular AE.

Vital signs, hematology, and clinical chemistry parameters from baseline to the end of the study will be presented as outlined above for continuous outcomes and reported to the DSMB at the regularly scheduled meetings. Incidence of changes in laboratory parameters from normal to abnormal, based on Boston Children’s Hospital reference ranges, will be identified and lab values listed by subject.

### Other considerations

Subjects will be considered to have complied with study treatment if ≥80% of the planned enteral nutrition was administered using the RELiZORB device. The frequency and percentage of subjects who were compliant will be presented.

The primary population analyzed for efficacy will be the full-analysis population (often referred to as intention-to-treat), defined as all subjects enrolled into the study. This population will include all enrolled subjects regardless of protocol deviations, compliance, or number of visits completed. A per-protocol population will be used for sensitivity analysis where results will be compared to the full-analysis population. The per-protocol population will exclude subjects with substantial deviations from the protocol (as determined by the investigator prior to database lock), <80% compliance, intercurrent illness for >20% of follow-up, or insufficient study visits (as determined by the investigator prior to database lock). The safety population will include a subset of the full-analysis population who received at least at least one treatment with RELiZORB and who have at least one adverse event grade 2 or higher.

The default method for calculating area under the curve when either the first follow-up visit (Day 7), last follow-up visit (Day 90), or both first and last follow-up visits are missing will be to evaluate from the first available follow-up visit to the last available follow-up visit. That is, AUC_F-L_ (area under the curve from first available until last available) will be calculated with no change in outcome indicated by AUC_F-L_ = 1. Sensitivity of the area under the curve to unknown data will be investigated using a best- and worse-case scenario method:

- Let $r_{i}= y_{i}/y_{1}$ denote the outcome at follow-up assessment day $t_{i}$ divided by the baseline value, where 𝑡 $\in$ **T** = (7, 14, 21, 28, 35, 42, 49, 60, 63, 70, 77, 84, 90).
- Best-case scenario: replace all missing $r_{c}$ with the value of **T** – {$r_{c}$} that is farthest from unity (1), representing the maximum change from baseline experienced by a given subject.
- Worse-case scenario: replace all missing $r_{c}$ with the value of **T** – {$r_{c}$} that is nearest to unity (1), representing the minimum change from baseline experienced by a given subject.

Outcomes assessed by area under the curve will be presented using the default method, the best-case scenario, and the worse-case scenario.

No interim analysis or early stopping rules are planned. The study will be monitored by an independent safety monitoring committee as outlined in section 8.0 Data and Safety Monitoring Board. The DSMB will have the right to recommend stopping the trial early if safety concerns arise. Additionally, an independent site monitor will perform periodic visits to review consent, eligibility and unanticipated adverse device effects (UADEs) for all enrolled subjects, as well as device accountability documents.

With a sample size of 32, the power to investigate subgroup effects will be limited even for large effect sizes; however, regression coefficient estimates will nevertheless be unbiased^22,23^. We will therefore explore the influence of several covariates, including gastrointestinal diagnosis, bowel length, presence/absence of colon, ileocecal valve, and stoma, as well as route of enteral nutrition administration. We will also adjust for temporary reduction or elimination of enteral nutrition due to intercurrent illness by including a yes/no indicator for any such reductions/eliminations.

### Sample Size

Historical review of patients followed by the Center for Advanced Intestinal Failure (CAIR) at Boston Children’s Hospital suggests that an average of 8 patients per year can be expected to meet the eligibility criteria, for a total of 32 subjects over 48 months. Additionally, patients currently followed by CAIR who are eligible and consent to the study will be enrolled, thus providing additional protection against attrition. Precision-based power calculations were determined with nQuery Advisor 7.0.

The primary outcome is the change in PN calories from baseline, assessed weekly at 13 assessments (study days 7, 14, 21, 28, 35, 42, 49, 56, 63, 70, 77, 84, and 90) using a baseline-adjusted time-weighted average (AUC_7-90_). It is believed that even a 10% reduction in PN calories would be clinically meaningful. In adults receiving teduglutide intended to improve enteral absorption in PN dependent patients with intestinal failure, a similar aim of 20% reduction was used to assess efficacy^24^. When this adult protocol was refined for a pediatric population, it was felt at least a 10% reduction in the volume of PN/IV support from baseline after 12 weeks of treatment was a reliable pharmacodynamic marker of increased intestinal absorptive capacity in pediatric short bowel syndrome patients who were dependent on PN/IV support. Although a 20% reduction from baseline in volume of PN/IV was used as the primary endpoint in the adult study, when adapted for a pediatric population, a ≥10% reduction was considered a valid pharmacodynamics (PD) marker of increased intestinal absorptive capacity, as agreed to by the expert clinicians with whom that study’s sponsors consulted (NPS Pharmaceuticals, Inc.). Based on the design of the teduglutide studies it was felt that the data provide similar support for a ≥10% reduction at Day 90 as a predictor of pharmacodynamic effect with the use of the RELiZORB device.

A reliable estimate for the standard deviation (SD) of AUC_7-90_ for the primary outcome in this patient population is unavailable. Unpublished data for 7 patients are available, but at least 4 of these would not meet the eligibility criteria for this study (ages 1-10; 1 had a STEP procedure; 3 have pancreatic involvement). Nevertheless, the mean±SD reduction (AUC_7-90_) for these 7 patients was 0.81±0.14, representing a 19% decrease over 90 days. Using this SD with a sample size of n=32, a 20% observed reduction in outcome would provide a 95% confidence interval (CI) = (0.75, 0.85), ruling out a true mean population reduction smaller than 15%. Conservatively, if the SD was doubled (SD=0.28), the 95% CI = (0.7, 0.9), excluding a true mean population reduction smaller than 10%, our target threshold In general, for any continuous outcome, a sample size of n=32 with two-side alpha=0.05 will provide 80% power to detect an effect size (mean/SD) as small as 0.51, considered a medium-sized effect^25^.

Dichotomous outcomes and will be presented as proportions. The variance for a proportion (p) is at a maximum, and its CI at its widest, when p=0.50. Proportions farther from 0.50 (in either direction) have smaller variances, and hence narrower CIs. A range of proportions with 95% CIs for a sample of 32 subjects is provided; small: p=0.20 (0.06 – 0.34); medium: p=0.50 (0.33 – 0.67); and large: p=0.80 (0.66 – 0.94).

## INFORMED CONSENT AND AUTHORIZATION FOR USE AND DISCLOSURE OF PROTECTED HEALTH INFORMATION

Written informed consent and authorization of use and disclosure of protected health information must be obtained from each subject (or the subject’s legally acceptable representative) before performing any study-specific screening/baseline period evaluations. For subjects who are minors, parents and guardians are asked to document their permission to allow their children to participate in research by signing an Informed Consent Form prior to enrollment. Consents will be available in English, Spanish, Portuguese, Mandarin, and Arabic. Additional translations will be made available as needed.

One copy of the signed informed consent form and authorization for use and disclosure of protected health information form will be given to the subject, and the investigator will retain the original. The informed consent form and authorization for use and disclosure of protected health information, which is prepared by the investigator or the site, must have been reviewed and approved by the investigator’s IRB/IEC and privacy board (if separate from the IRB/IEC) before the initiation of the study. The informed consent form must contain the 20 elements of informed consent described in International Council on Harmonization E6, Section 4.8. The authorization for use and disclosure of protected health information must contain the elements required by Title 45 of the Code of Federal Regulations, Section 164.508(b) and any local regulations for valid authorizations.

## STUDY DOCUMENTATION

### Investigator Information

Investigator information is included in the study procedures manual, which is updated as needed.

### Investigator’s Study Files

Documentation about the investigator and study staff, the IRB, and the institution is required before study site initiation. Copies of these documents will be kept electronically in a BCH firewall secured SharePoint site along with the following supplemental information: a delegation of authority log, the Investigator’s Brochure, the clinical protocol and amendments, safety information, information about investigational product, information about biological samples, and information about the laboratory, the study procedures manual and study logs, CRFs*,* records of site monitoring activities, and correspondence between sponsor-investigator and the study monitor.

The sponsor-investigator will be responsible for maintaining backup of all CRF data. The investigator is responsible for maintaining backup of all electronic data systems used for primary documentation or source documentation. Electronic data will be backed up periodically. Backup records must be stored at a secure location on site, and backup and recovery logs must be maintained to facilitate data recovery.

Changes to any electronic records require an audit trail, in accordance with Title 21 of the Code of Federal Regulations, Section 11.10(e), and should indicate who made the changes, when the changes were made, and why the changes were made. An audit trail is defined as a secure, computer-generated, time-stamped electronic record that will allow reconstruction of the course of events relating to the creation, modification, and deletion of an electronic record. Audit trails must be created incrementally, in chronological order, and in a manner that does not allow new audit trail information to overwrite existing data. Finally, audit trails should be in a readable format and readily available at the site and any other location where electronic study records are maintained.

Audit trails are generated automatically for database entries. The investigator is responsible for maintaining audit trails of all electronic data systems used for source documentation.

### Case Report Forms and Source Documentation

The investigator must make study data accessible to the site monitor and the appropriate regulatory authority inspectors. The site monitor will be responsible for reviewing study documents associated with consent, eligibility unanticipated adverse device effects (UADEs), and device accountability, including CRFs, source documents and study logs.. A copy of the final CRFs will be provided to the investigator in portable document format on computer disc after study closure to be kept in the investigator’s study files.

### Retention of Study Documents

According to International Council on Harmonization E6, Section 4.9, all CRFs, as well as supporting paper and electronic source documentation and administrative records, must be retained by the investigator for a minimum of 2 years after notification that the appropriate regulatory authority has approved the product for the indication under study, notification that the entire clinical investigation will not be used in support of a marketing application, or notification that the marketing application was not approved. These documents will be retained for a longer period, however, in accordance with BCH policy for research records. If the investigator relocates, retires, or withdraws from the clinical study for any reason, all records required to be maintained for the study will be transferred to an agreed-upon designee, such as another investigator at the institution where the study was conducted.

Audit trails for electronic documents must be retained for a period at least as long as that required for the subject electronic records to which they pertain. The investigator must retain either the original of the audit trails or a certified copy of the audit trails.

## CONFIDENTIALITY

### Data

The investigator must keep all information confidential about the nature of the proposed investigation provided by the study monitor to the investigator (with the exception of information required by law or regulations to be disclosed to the IRB, the subject, or the appropriate regulatory authority).

### Subject Anonymity

The anonymity of participating subjects must be maintained. Subjects will be identified by an assigned subject number on CRFs and other documents retrieved from the site or sent to the study monitor, regulatory agencies, central laboratories, or blinded reviewers. Documents that identify the subject (e.g., the signed informed consent form) must be maintained in strict confidence by the investigator, except to the extent necessary to allow auditing by the appropriate regulatory authority, or the study monitor.

## PROTOCOL COMPLIANCE

Substantive changes in the protocol include changes that affect the safety of subjects or changes that alter the scope of the investigation, the scientific quality of the study, the experimental design, dosages, assessment variable(s), the number of subjects treated, or the subject-selection criteria. Such changes must be prepared as a protocol amendment by the sponsor-investigator. A protocol amendment must receive IRB approval before implementation. In parallel with the IRB approval process, the protocol amendment will be submitted to the appropriate regulatory authority as an amendment to the regulatory submission under which the study is being conducted. If a protocol amendment requires changes in the informed consent form, the revised informed consent form prepared by the investigator must also be approved by the IRB before implementation.

Departures from the protocol are allowed only in situations that eliminate an immediate risk to a subject and that are deemed crucial for the safety and well‑being of that subject. The investigator or the attending physician also will contact the IRB as soon as possible in the case of such a departure. These departures do not require preapproval by the IRB; however, the IRB must be notified in writing as soon as possible after the departure has been made. In addition, the investigator will document in the subject’s CRF the reasons for the protocol deviation and the ensuing events.

## STUDY MONITOR FUNCTIONS AND RESPONSIBILITY

The study monitor, in accordance with the sponsor-investigator’s requirements, will ensure that the clinical study is conducted and documented properly by carrying out the activities outlined in International Council on Harmonization E6, Section 5.18.4. The study monitor will perform periodic visits to review consent, eligibility and unanticipated adverse device effects (UADEs) for all enrolled subjects, as well as device accountability documents.

## GENERAL INFORMATION

The investigator will refer to the study procedures manual, and any other information provided about this investigational product and details of the procedures to be followed during the conduct of this clinical study.

# REFERENCES

1. Cagir, B., Short-Bowel Syndrome, Medscape.com, available at emedicine.medscape.com/article/193391-overview#showall; accessed 26 December 2012.
2. Vanderhoof JA, Langnas AN. Short-bowel syndrome in children and adults. Gastroenterology. 1997 Nov;113(5):1767-78.
3. Duro D, Kamin D, Duggan C. Overview of pediatric short bowel syndrome. J Pediatr Gastroenterol Nutr. 2008 Aug;47 Suppl 1:S33-6.
4. Chang MI, Puder M, Gura KM. The use of fish oil lipid emulsion in the treatment of intestinal failure associated liver disease (IFALD).Nutrients. 2012 Nov 27;4(12):1828-50.
5. Quirós-Tejeira RE, Ament ME, Reyen L, Herzog F, Merjanian M, Olivares-Serrano N, Vargas JH. Long-term parenteral nutritional support and intestinal adaptation in children with short bowel syndrome: a 25-year experience. J Pediatr. 2004 Aug;145(2):157-63.
6. Cole CR, Hansen NI, Higgins RD, Ziegler TR, Stoll BJ; Eunice Kennedy Shriver NICHD Neonatal Research Network. Very low birth weight preterm infants with surgical short bowel syndrome: incidence, morbidity and mortality, and growth outcomes at 18 to 22 months. Pediatrics. 2008 Sep;122(3):e573-82.
7. Squires RH, Duggan C, Teitelbaum DH, Wales PW, Balint J, Venick R, Rhee S, Sudan D, Mercer D, Martinez JA, Carter BA, Soden J, Horslen S, Rudolph JA, Kocoshis S, Superina R, Lawlor S, Haller T, Kurs-Lasky M, Belle SH; Pediatric Intestinal Failure Consortium. Natural history of pediatric intestinal failure: initial report from the Pediatric Intestinal Failure Consortium. J Pediatr. 2012 Oct;161(4):723-8.e2.
8. Colomb V, Jobert-Giraud A, Lacaille F, Goulet O, Fournet JC, Ricour C. Role of lipid emulsions in cholestasis associated with long-term parenteral nutrition in children. JPEN J Parenter Enteral Nutr. 2000 Nov-Dec;24(6):345-50.
9. Buchman AL: Complications of long-term home total parenteral nutrition: their identification, prevention and treatment. Dig Dis Sci 2001, 46:1-18.
10. Gura KM, Duggan CP, Collier SB, Jennings RW, Folkman J, Bistrian BR, Puder M. Reversal of parenteral nutrition-associated liver disease in two infants with short bowel syndrome using parenteral fish oil: implications for future management. Pediatrics. 2006 Jul;118(1):e197-201.
11. Suita S, Masumoto K, Yamanouchi T, Nagano M, Nakamoura M. Complications in neonates with short bowel syndrome and long-term parenteral nutrition. J Parenteral Enteral Nutr 1999;23:S106-9.
12. Javid PJ, Collier Sharon, Richardson D, Iglesias J, Gura K, Lo C, Kim HB, Duggan CP, Jaksic T. The role of enteral nutrition in the reversal of parenteral nutrition-associated liver dysfunction in infants. J Pediatr Surg (2005) 40, 1015-1018.
13. Yang CF, Lee M, Valim C, et al. Persistent alanine aminotransferase elevations in children with parenteral nutrition-associated liver disease. J Pediatr Surg. 2009 Jun;44(6):1084-7.
14. Tillman et al Enteral fish oil for treatment of parenteral nutrition-associated liver disease in six infants with short-bowel syndrome. Pharmacotherapy. 2011 May;31(5):503-9.
15. Yang Q, Ayers K, Chen Y, Helderman J, Welch CD, O'Shea TM. Early enteral fat supplement and fish oil increases fat absorption in the premature infant with an enterostomy. J Pediatr. 2013 Aug;163(2):429-34.
16. Yang Q, Ayers K, Chen Y, O'Shea TM. Early enteral fat supplementation improves protein absorption in premature infants with an enterostomy. Neonatology. 2014;106(1):10-6.
17. Yang Q, Ayers K, Welch CD, O'Shea TM. Randomized controlled trial of early enteral fat supplement and fish oil to promote intestinal adaptation in premature infants with an enterostomy. J Pediatr. 2014 Mar 12.
18. Donaldson, J et al. The effectiveness of enzymatic replacement therapy measured by turbidimetry and the lipaemic index in exocrine pancreatic insufficient young, growing pigs, fed a high-fat diet. Advances in Medical Science 2009, 54, 7-13.
19. Bligh EG, Dyer WJ. A rapid method of total lipid extraction and purification. Can J Biochem Physiol 1959; 37:911-7.
20. Freedman S, Orenstein D, Black P, Brown P, McCoy K, Stevens J, Grujic D, Clayton R. Increased Fat Absorption From Enteral Formula Through an In-line Digestive Cartridge in Patients With Cystic Fibrosis. J Pediatr Gastroenterol Nutr. 2017 Jul;65(1):97-101.
21. Stevens J, Wyatt C, Brown P, Patel D, Grujic D, Freedman SD.Absorption and Safety With Sustained Use of RELiZORB Evaluation (ASSURE) Study in Patients With Cystic Fibrosis Receiving Enteral Feeding.. J Pediatr Gastroenterol Nutr. 2018 Oct;67(4):527-532.
22. Green S. How many subjects does it take to do a regression analysis? Multivariate Behav Res 1991;26(3):499e510.
23. Austin PC, Steyerberg EW. The number of subjects per variable required in linear regression analysis. J Clin Epidemiol 2015 Jun;68(6):627-36.
24. Jeppesen, P. B., et al. Teduglutide reduces need for parenteral support among patients with short bowel syndrome with intestinal failure. Gastroenterology 2012 143(6): 1473-1481 e1473.
25. Cohen J. A Power Primer. Psychol Bull. 1992 Jul;112(1):155-9. doi: 10.1037//0033-2909.112.1.155. PMID: 19565683
