# Supplementary material for: Fat digestion using RELiZORB in children with short bowel syndrome who are dependent on parenteral nutrition: Protocol for a 90-day, phase 3, open labeled study
Source: PLoS One. 2023 Mar 1;18(3):e0282248. doi: 10.1371/journal.pone.0282248 (PMC9977023; doi:10.1371/journal.pone.0282248)
Supplement: S3 File — (PDF) [file pone.0282248.s003.pdf]

RESEARCH CONSENT FORM

Use Plate or Print:

MRN#:

DOB:

Subject's Name:

Gender

Protocol Title: A 90 day, Phase 3, Open Labeled Exploratory Study of RELiZORB to Evaluate Safety, Tolerability, and Nutrient Absorption in Children with Short Bowel Syndrome who are Dependent on Parenteral Nutrition

Principal Investigator: Mark Puder, MD, Ph.D.

This consent form gives you important information about a research study. A research study helps scientists and doctors learn new information to improve medical practice and patient care.

Participation in this research study is voluntary. You are free to say yes or no and your decision will not impact the care your child receives at Boston Children's Hospital. You or your child can withdraw from the study at any time.

A description of the study and its risks, potential benefits and other important information are in this consent form. Please read this consent form carefully and take your time making a decision. The form may contain words that you do not understand. Please ask questions about anything you do not understand. We encourage you to talk to others (for example, your friends, family, or other doctors) before you decide to participate in this research study.

How are individuals selected for this research study?

Your child is being invited to take part in a research study for a device called RELiZORB because your child has short bowel syndrome (SBS) and receives both parenteral (IV) nutrition and enteral nutrition (tube feeding). RELiZORB has been approved by the FDA to be used in patients 5 years and older to digest fats in tube feed formulas, but it has not been studied yet in children with SBS. SBS is a life-long condition caused by loss of most of the small intestine, such that your child will need special nutrients given through the veins (known as intravenous or IV parenteral nutrition/IV fluids or PN/IV).

Why is this research study being conducted?

In this research study we want to learn more about the RELiZORB device that has been FDA cleared for use in patients who receive tube feeds to help them digest fats. We want to learn if using this device when administering enteral nutrition (tube feeds) will allow children with short bowel syndrome to absorb the nutrition that they get from their food better and have better weight gain and growth. The purpose of this study is to help answer the following questions:

- How safe is the RELiZORB device to use with enteral nutrition (tube feeding) in children with SBS?
- Can using the RELiZORB device with enteral nutrition (tube feeding) help children with SBS absorb more food?
- Will using the RELiZORB device with enteral nutrition (tube feeding) decrease the amount of PN (IV

nutrition) a child with SBS needs?

This study involves testing the Food and Drug Administration (FDA)-approved device, RELiZORB that is used in patients 5 years and older to digest fats in tube feed formulas. This device has not yet been evaluated by the FDA for treatment of SBS in children. This device has also not yet been evaluated in children under the age of 5. In this study, we want to find out more about the side effects (problems and reactions) of the RELiZORB device when used in children with short bowel syndrome. Every child in this study will receive the study device RELiZORB to administer their child's enteral nutrition (tube feeding). Information from this research may help decide whether the study device should be approved by the FDA for use in children with short bowel syndrome.

Who is conducting this research study, and where is it being conducted?

The study being conducted is a single-center study; currently only at Boston Children's Hospital. Dr. Mark Puder (Research investigator) will conduct the study.

Your child's health care provider may be a research investigator for this research and as an investigator, is interested in both your child's clinical welfare and in the conduct of this study. Before entering this study or at any time during the research, you may ask for a second opinion about your child's care from another health care provider who is in no way associated with this study. You are not under any obligation to participate in any research project offered by your child's health care provider. If you choose not to allow your child to participate, your child's care at Boston Children's Hospital and/or with your child's health care provider will not be affected in any way at all.

How many people will participate in this research study?

Up to 32 children will take part in this study at Boston Children's Hospital.

What do I have to do if I am in this research study?

Taking part in the study is entirely voluntary. It is up to you whether or not to allow your child to take part. If you choose to allow your child to participate she/he/they will be in this research study for approximately 90 days.

If you decide to allow your child to take part, you must sign and date the consent at the end of this form. Even after signing the consent, you are free to stop your child's participation and leave the study at any time without giving a reason.

There may be reasons why it would not be good for your child to be in the study. The study doctor will ask you about your child's health and medical history. This information, along with the results of screening tests, will help your study doctor decide whether or not your child can take part in the study. If your child is not allowed to take part in the study, the study doctor will have you continue with the routine medical care that is provided to your child now.

If you decide not to allow your child to take part or if you withdraw your child from the study at a later time, there will be no penalty or loss of benefits to which your child is otherwise entitled. This would not change the routine medical care your child would otherwise receive.

In this study all children will receive their enteral nutrition (tube feeds) through a RELiZORB enzyme cartridge. They will do this for a total of 90 days.

During this time your child will be seen at Boston Children's Hospital for these visits:

- Screening/Enrollment (3 days)
- Baseline (first day of treatment)
- Follow up visits (Days 7, 14, 28, 60, 90)

If the screening information shows that your child meets the requirements, then you will be asked to participate in the study. If the screening information shows that your child cannot be in the research study, the research investigator will discuss other options with you and/or refer you back to your child's regular health care provided. This may also be discussed at the baseline visit as well.

You will be asked to keep diaries of when your child used the study device, and if he/she/they had any tube feeds that did not use the device, and how frequently they go to the bathroom. To assist with recording your child's food, enteral and parenteral intake, we will provide access to a phone application (app) to help with keeping track of your child's nutritional intake. We also want to know how your child is feeling during this study and will ask you to pay close attention if your child feels different from how they feel usually. The diaries will have questions about if they have more frequent stools or if the stool changes to be more like diarrhea. It will also have questions about urine color. Your child will have blood drawn at every visit, except the first day of treatment visit.

A study coordinator will call you each day to check in, answer questions you may have, ensure you are using the device correctly, ask about any side effects, and remind you to complete your daily diary.

The table below shows what will occur at each study visit if you decide to join the research study.

Measurements/ Evaluations	Screening	Enrollment Period (3-days)	Day 1 (Baseline)	Day 7	Day 14 and Day 28 And Day 60	Day 90
Medical history	X					
Study medication instructions			X			
Physical examination + vital signs	X		X	X	X	X
Blood Draw	X			X	X	X
Education with Study Dietician (includes nutritional assessment and nutritional diary review)	X		X	X	Weekly	X
Three-day continuous stool collection		X				X
Study Coordinator phone call		Daily	Daily	Daily	Daily	Daily
Device assessment	X		X	X	X	X
Review of daily diaries for nutrition, Adverse events and device assessment			X	X	Weekly	X
Assess if any medical/surgical procedures have been done			X	X	X	X
Length of Visit	2-3 hours		2-3 hours	1-2 hours	1-2 hours	1-2 hours

Medical history

We will ask you about your child's past medical history. This will only occur once at the initial screening visit.

Study medication instructions

We will show you how to use the device to administer your child's enteral nutrition (tube feeds) at home. You will receive additional instructions to take home. We will ask you to give your child the first feeding using the device with our supervision so that we know that you are comfortable with how to do this. You will be given instructions on how to store and give the study device to your child, and then we will give you a supply of the study devices to take home.

Vital signs and physical examination

The study doctor will conduct a physical exam that will include listening to your child's heart and lungs, taking their blood pressure, pulse rate and temperature. Your child will also be weighed and have their height measured. These procedures will occur at every visit.

Blood draw

At every visit except the baseline (Day 1) visit, we will take about a teaspoon of blood from your child to look at different levels in their blood and to make sure it is safe for them to take use the RELiZORB enzyme cartridge. The total amount of blood drawn over the entire study will be about 2 tablespoons.

Stool sample

We will also ask you to collect your child's stool for 72 hours prior to the first and last study visit. We will provide you with containers to place your child's stool for the 72 hours prior to the visits. These containers should be refrigerated until the collection is complete. This stool collection will allow us to test how much fat is in your child's stool. A member of the study team will call you to remind you to bring the diaries and stool collection. It is important to collect all stool from 72 hours to accurately test how much fat is present.

Study dietician education, nutritional assessment, and nutritional diaries

You will meet with the study dietician and discuss your child's nutritional intake. This includes both enteral nutrition (tube feeds) and parenteral nutrition. The dietician will teach you how to record your child's nutritional intake each day. At every visit we will ask you about how much your child eats and drinks, and about how much of the special nutrition (parental nutrition) they get in their veins. You will be given access to an electronic diary (or paper if preferred) and access to a nutrition phone application prior to going home, to record every day how much your child eats and drinks, goes to the bathroom and about how much of the special nutrition (parental nutrition) they get in their veins. A member of the study team will call you to remind you to bring the diaries with you to the study visit. We will ask you to complete a diary for every day between all study visits.

In between Hospital Study Visits

A study team member will attempt daily telephone calls with you in-between your child's study visits at the hospital. During weekly phone calls, the study doctor or nurse will ask the same questions as during the hospital visits, including review of the nutritional and stool/urine diaries with you. After reviewing all of the information, the study doctor or your Center for Advanced Intestinal Rehabilitation (CAIR) physician/team may change your child's parenteral nutrition or G-tube feeding prescriptions. If a change is made, your child may need lab tests before the next hospital visit. You will be asked to weigh your child at home each week.

What are the risks of this research study? What could go wrong?

Some procedures or treatments used in this research may present risks that are not well-known or understood. Therefore, there may be unforeseeable risks associated with participating in this research.

RELiZORB has been studied for patients over 5 years of age with cystic fibrosis to help them absorb their enteral nutrition (tube feeds) better. RELiZORB is digestive enzyme cartridge designed to digest fats contained in enteral formulas, mimicking the function of the digestive enzyme lipase that is normally secreted by the pancreas, the body's digestive organ. By digesting fats from enteral formulas it is better absorbed by the patient. RELiZORB is a single-use, point-of-care digestive enzyme cartridge that connects in-line with existing enteral feeding pump tubing sets and patient extension sets or enteral feeding tubes.

RELiZORB is comprised of a clear cylindrical, plastic cartridge with a single inlet connection port and a single purple outlet connection port. The inlet and outlet ports of RELiZORB are intended to connect in-line with enteral feeding pump tubing sets and patient extension sets or enteral feeding sets. Inside the cartridge, there are small white beads. The digestive enzyme, lipase, is attached to the small white beads. The lipase-bead complex, iLipaseTM (immobilized lipase), is retained within the cartridge during use by filters on both ends of the cartridge. The fat in enteral formulas is digested as it comes in contact with iLipase as the formula passes through the cartridge. Your child will not ingest any of the lipase contained in the cartridge. Each cartridge comes in a single pouch which should be stored with your other enteral feeding pump supplies and should be kept away from children and not be allowed to freeze. If your child is admitted to the hospital during the study period, please make sure that you bring your supply of RELiZORB devices with you so that your child can continue to receive their enteral nutrition using the device. It is important to make sure that you take any unused devices back with you upon discharge from the hospital.

As your child advances on enteral feeding, they may experience feeding intolerances. Symptoms may include:

- Diarrhea or constipation
- Nausea or vomiting
- Bloating or swelling
- Gas and/or foul smelling stool
- Poor appetite
- Weight loss or trouble gaining weight
- Fatigue

Research on using this device in animals has not revealed any special risks. There are no contraindications in humans.

There may be other side effects of using RELiZORB enzyme cartridge that we just don't know about yet. If any new information is learned about potential risks of RELiZORB use, it will be shared with all participants.

Risks associated with procedures:

If blood is collected from a peripheral vein in your child's arm, he or she may have pain, bleeding, swelling, or bruising around the vein where blood is collected. There is a risk of infection from any blood draw. Your child may feel dizzy or faint. If blood is drawn from your child's central line there is a risk for infection.

The device to be used in this research study will not be given to you in a child resistant package. It must be stored at room temperature (between 59ºF and 86ºF) and out of the reach of children.

> I understand that the device is not in a child resistant package and understand that I must make sure that it is stored safely and out of the reach of children.____________(initials of parent/guardian)

What are the benefits of this research?

There may be a direct benefit to your child if the study device works as it is intended, meaning he/she might absorb nutrients better. However, because the effects of this study device are still not completely known in patients with short bowel syndrome, this research may not help your child right now. When we finish the research, we hope that we will know more about your child's condition. This may help other children/adults with this condition in the future.

Why would I be taken off the study early?

The study doctor may decide that it is best for your child to stop taking part in the study. If so, (s) he will discuss with you the reasons why your child may have to leave the study. For example, your child may have to leave the study without your consent if your child needs other treatment, does not follow the study plan, has a study-related injury, or for another reason. The Sponsor, the Institutional Review Board, the Food and Drug Administration or another regulatory authority may also end the study at any time.

If your child stops taking part in the study or is withdrawn from the study, your child may be asked to have medical tests and follow-up to check your child's health and safety. Your child will not be able to continue using the study device, and you will need to bring back all empty packets and unused study devices. Your child's healthcare provider may offer an alternative treatment for your child's short bowel syndrome.

Any data about your child (including your child's personal health information) that has already been collected will remain part of the study database and may not be removed. This is in order to maintain the reliability of the study's results and to satisfy legal and regulatory requirements.

If I do not want to take part in this research, what are the other choices?

If you do not want to allow your child to participate in this research, your child will continue with the current medical management he/she is receiving at Boston Children's Hospital. You are under no obligation to agree to participate in this study. Refusal to participate in this research study will in no way interfere with current or future care at Boston Children's Hospital

Will I receive any payments?

You will be paid **$***100.00* for each research visit that your child completes at Boston Children's Hospital. You will also be compensated for completion of the diaries that you have been requested to fill out daily. We understand that filling out the diary daily is a large commitment from you and we will offer you *$5.00* additionally per day for completed diaries and for bringing in the completed forms. This will add up to a total of **$**1,150 if your child completes all of the research visits.

If you live outside a 50 mile radius of BCH, you will be reimbursed for roundtrip travel expenses (i.e., airfare, gasoline, parking) and hotel stays at the Inn at Longwood Medical (or up to $225.00 a night if you stay at another hotel) .For gas milage, we will calculate the appropriate reimbursement based on the IRS standard mileage rate for the calendar year and add that amount to your ClinCard. All reimbursements will be made through ClinCard.

Additionally, at each visit, you will receive a parking voucher and $25 for meal expenses. This $25 will also be loaded onto the ClinCard at each visit.

If your child leaves the research early, or if we have to take your child out of the research, you will only be paid for the visits your child has completed.

This research study will use a service called ClinCard® by the company Greenphire, www.greenphire.com, to manage all payments associated with your child's participation in study visits, your time and travel related to participation in the study. ClinCard/Greenphire will provide documentation for filing your taxes (1099 form), to the hospital, and may ask for your name and social security number using a secure website to meet that federal requirement. Boston Children's Hospital or the sponsor has contracted with ClinCard/Greenphire to provide this service but Boston Children's Hospital and ClinCard/Greenphire are separate entities and have no other relationship. ClinCard/Greenphire is solely responsible for the security of any information you provide to them.

You will be issued a ClinCard, which is a specially designed debit card for clinical research onto which your funds will be loaded as appropriate. When a study visit is completed, funds will be loaded onto your card. The funds will be available within 1 day and can be used as you wish.

In addition to your travel expenses, you will be paid up to $1,150.00 for your time spent in the research. Since this amount will be greater than the minimum reporting requirements set by the Internal Revenue Service or IRS (>$600/year), Boston Children's Hospital must report this to the IRS and will give you a 1099 form because the payment you receive for this study will be considered taxable income. Boston Children's Hospital

will not deduct taxes from this payment. You will be responsible for reporting this payment when you file your tax return. We will ask for your social security number for tax reporting purposes, but it will not be stored with any other research data.

Are there costs associated with this research?

Although research funds will pay for some research-related items and services, we may bill your child's health insurer for routine items and services your child would have received even if your child did not take part in this research. You will be responsible for payment of any deductibles and co-payments required by your insurer for this routine care or other billed care. If you have any questions about costs to you that may result from taking part in the research, please speak with the research staff.

Compensation for Injury:

We will offer you the care needed to treat any injury your child experiences that directly results from taking part in this research. We reserve the right to bill your child's insurance company or other third parties, if appropriate, for the care your child gets for the injury. We will try to have these costs paid for, but you may be responsible for some of them. For example, if the care is billed to your insurer, you will be responsible for payment of any deductibles and co-payments required by your insurer.

Injuries sometimes happen in research even when no one is at fault. There are no plans to pay you or give you other compensation for an injury, should one occur. However, you are not giving up any of your legal rights by signing this form. If you think your child has been injured or has experienced a medical problem as a result of taking part in this research, tell the person in charge of the research as soon as possible. The researcher's name and phone number are listed in this consent form.

If you go to the Emergency Room or to another hospital or doctor it is important that you tell them that your child is in this research. If possible, you should give them a copy of this consent form.

Other information for you to know:

If we find out about new information from this research or other research that may affect your child's health, safety or willingness to stay in this research we will let you know as soon as possible.

If, during your participation in this research, there is reason to believe that child abuse is of concern, the research team must follow state law by filing a child abuse report with the Department of Children and Families (DCF). Research records might be court ordered for use in court hearings. The research team will make every reasonable effort to protect the confidentiality of research data, though it is possible that a court might demand the release of information gathered during this research.

Other information that may help you:

Boston Children's Hospital has developed a web-based, interactive educational program for parents called "A Parent's Guide to Medical Research." To find out more about research at Children's, please visit the program at www.researchchildren.org.

Boston Children's Hospital is interested in hearing your comments, answering your questions, and responding to any concerns regarding clinical research. If you have questions or concerns, you may email IRB@childrens.harvard.edu or call (617) 355-7052 between the hours of 8:30 and 5:00, Monday through Friday.

Who may see, use or share your health information?

If your child is not currently a patient at Boston Children's Hospital and does not have a medical record at Boston Children's Hospital, one may be created for your child for his/her participation in this research. Your child may also be required to register as a patient of Boston Children's Hospital in order to participate in this research.

A copy of this consent form will be placed in your child's medical record. If your child does not have a medical record at Boston Children's Hospital, one will be created for you.

Information collected during this research will become part of your child's medical record, if the information is related to the care your child receives at Boston Children's Hospital. Medical records are considered permanent records; therefore, information cannot be deleted from the record. Medical records are available to health care professionals at Boston Children's Hospital and may be reviewed by Hospital staff when carrying out their responsibilities; however, they are required to maintain confidentiality in accordance with applicable laws and Hospital policies. Information contained in your child's medical record may not be given to anyone unaffiliated with Boston Children's Hospital in a way that could identify you without written consent, except as required or permitted by law.

A description of this clinical trial will be available on http://www.ClinicalTrials.gov, as required by U.S. law. This web site will not include information that can identify you. At most, the Web site will include a summary of the results. You can search this web site at any time.

What should you know about HIPAA and confidentiality?

Your health information is protected by a law called the Health Information Portability and Accountability act (HIPAA). In general, anyone who is involved in this research, including those funding and regulating the study may see the data, including information about you. For example, the following people might see information about you:

- Research staff at Boston Children's Hospital involved in this study;
- Medical staff at Boston Children's Hospital directly involved in your care that is related to the research or arises from it;
- Other researchers and centers that are a part of this study, including people who oversee research at that hospital;
- People at Boston Children's Hospital who oversee, advise, and evaluate research and care. This includes the ethics board and quality improvement program;
- People from agencies and organizations that provide accreditation and oversight of research;
- People that oversee the study information, such as data safety monitoring boards, clinical research organizations, data coordinating centers, and others;
- Sponsors or others who fund the research, including the government or private sponsors.
- Companies that manufacture drugs or devices used in this research;
- Federal and state agencies that oversee or review research information, such as the Food and Drug Administration, the Department of Health and Human Services, the National Institutes of Health, and public health and safety authorities;
- People or groups that are hired to provide services related to this research or research at Boston Children's Hospital, including services providers, such as laboratories and others;
- And/or your health insurer, for portions of the research and related care that are considered billable.

If some law or court requires us to share the information, we would have to follow that law or final ruling.

Some people or groups who get your health information might not have to follow the same privacy rules. Once your information is shared outside of Boston Children's Hospital, we cannot promise that it will remain private. If you decide to share private information with anyone not involved in the study, the federal law designed to protect privacy may no longer apply to this information. Other laws may or may not protect sharing of private health information. If you have a question about this, you may contact the Boston Children's Hospital Privacy Officer at (857) 218-4680, which is set up to help you understand privacy and confidentiality.

Because research is ongoing, we cannot give you an exact time when we will destroy this information. Researchers continue to use data for many years, so it is not possible to know when they will be done.

We will also create a code for the research information we collect about you so identifying information will not remain with the data and will be kept separately. The results of this research may be included in data submissions or filings with regulatory agencies, published in a medical book or journal, or be used for teaching purposes. However, your name or identifying information will not be used without your specific permission.

The Sponsor may also use the information about the results of this study for other research purposes which may include:

- Reviewing the safety or effectiveness of the RELiZORB enzyme cartridge
- Conducting performance reviews of the RELiZORB enzyme cartridge or retrospective reviews of the study or the study data
- Evaluating other products or therapies for patients
- Developing a better understanding of SBS or
- Improving the design and efficiency of future clinical trials

Your privacy rights

If you want your child to participate in this research study, you must sign this form. If you do not sign this form, it will not affect your child's care at Boston Children's Hospital now or in the future and there will be no penalty or loss of benefits. You can withdraw your child from the study and end your permission for Boston Children's Hospital to use or share the protected information that was collected as part of the research; however you cannot get back information that was already shared with others. Once you remove your permission, no more private health information will be collected. If you wish to withdraw your child's health information, please contact the research team.

You may have the right to find out if information collected for this study was shared with others for research, treatment or payment. You may not be allowed to review the information, including information recorded in your child's medical record, until after the study is completed. When the study is over, you will have the right to access the information again. To request the information, please contact the Hospital's Privacy Officer at (857) 218-4680.

Contact Information

I understand that I may use the following contact information to reach the appropriate person/office to address any questions or concerns I may have about this study.

I can call…	At	If I have questions or concerns about
Investigator:<br>Mark Puder, MD, Ph.D.	Phone: 617-355-1838<br>Pager: 617-355-7243<br>Page # 1650	▪ General questions about the research<br>earch-related injuries or emergencies<br>▪ Any research-related concerns or complaints
Research Contact:<br>Savas Tsikis, MD	Phone: 732-278-5289	▪ General questions about the study<br>▪ Research-related injuries or emergencies<br>▪ Any research-related concerns or complaints
Institutional Review Board	Phone: **617-355-7052**	▪ Rights of a research participant<br>▪ Use of protected health information.<br>▪ Compensation in event of research-related injury<br>▪ Any research-related concerns or complaints.<br>▪ If investigator/research contact cannot be reached.<br>▪ If I want to speak with someone other than the Investigator, Research Contact or research staff.

Documentation of Informed Consent and Authorization

- I have read this consent form and was given enough time to consider the decision to participate in this research.
- This research has been satisfactorily explained to me, including possible risks and benefits.
- All my questions were satisfactorily answered.
- I understand that participation in this research is voluntary and that I can withdraw at any time.
- I am signing this consent form prior to participation in any research activities.
- I give permission for participation in this research and for the use of associated protected health information as described above (HIPAA).

Parent/Legal Guardian Permission:

If the child to be involved in this research is a foster child or a ward of the state please notify the researcher or their staff who is obtaining your consent.

■ ____________________ ____________________ ____________________

Date (MM/DD/YEAR) Signature of **Parent #1** or **Legal Guardian** Relationship to child

Child Assent

■ ____________________ ____________________

Date (MM/DD/YEAR) Signature of **Child/Adolescent Participant**

■ If child/adolescent's assent is **not** documented above, please indicate reason below (check one):

☐ Assent is documented on a separate IRB-approved assent form

☐ Child is too young

☐ Other reason (e.g. sedated), please specify: ____________________

____

____

Research Investigator /or Associate's Statement & Signature

- I have fully explained the research escribed above, including the possible risks and benefits, to all involved parties (participant /parents/legal guardian as applicable).
- I have answered and will answer all questions to the best of my ability.
- I will inform all involved parties of any 5d/changes (if applicable) to the research procedures or the risks and benefits during or after the course of the research. have provided a copy of the consent form signed by the participant / parent / guardian and a copy of the hospital's privacy notification (if requested).

■ ____________________ ____________________+

Date (MM/DD/YEAR) Signature of **Research Investigator or Associate**

Witness Statement & Signature

A witness must be present for the entire consent process in the following situations (please check the appropriate box)

☐ The individual cannot read and this consent document was read to the participant or legal representative, **or**

☐ The individual has certain communication impairments that limit the participant's ability to clearly express consent **or**

☐ Situations where the IRB requests a witness be present: please specify

I confirm that the information in this consent form was accurately explained to the participant, parent or legally authorized representative, the individual appeared to understand the information and had the opportunity to ask questions, and that informed consent was given freely.

____________________	______________________________
Date (MM/DD/YEAR)	Signature of Witness
