# Supplementary material for: Fat digestion using RELiZORB in children with short bowel syndrome who are dependent on parenteral nutrition: Protocol for a 90-day, phase 3, open labeled study
Source: PLoS One. 2023 Mar 1;18(3):e0282248. doi: 10.1371/journal.pone.0282248 (PMC9977023; doi:10.1371/journal.pone.0282248)
Supplement: S4 File — (DOCX) [file pone.0282248.s004.docx]

**DATA AND SAFETY MONITORING BOARD (DSMB)**

**CHARTER**

**1. Introduction**

This is the charter of the independent Data and Safety Monitoring Board (DSMB) convened for the A 90 day, Phase 3, Open Labeled Exploratory Study of RELiZORB to Evaluate Safety, Tolerability, and Nutrient Absorption in Children with Short Bowel Syndrome who are Dependent on Parenteral Nutrition study.

**2. Responsibilities of the DSMB**

The DSMB is responsible for safeguarding the interests of study participants, assessing the safety and efficacy of study procedures, and for monitoring the overall conduct of the study.

The DSMB is independent of the study team and is required to provide recommendations about starting, continuing, and stopping the study. In addition, the DSMB is asked to make recommendations, as appropriate, about:

- Adverse events
- Efficacy of the study intervention
- Benefit/risk ratio of procedures and participant burden
- Selection, recruitment, and retention of participants
- Adherence to protocol requirements
- Completeness, quality, and analysis of measurements
- Amendments to the study protocol and consent forms
- Participant safety
- Notification of and referral for abnormal findings

**3. DSMB Membership, Organization and Interactions**

The DSMB will consist of 3 physicians and a biostatistician independent of the principal investigator and her/his colleagues. The DSMB will meet quarterly to review the status of recruitment according to a written data and safety monitoring plan.

**4. Scheduling, Timing, and Organization of Meetings**

DSMB meetings will be held by Zoom meeting, unless the DSMB requests otherwise. The purposes of the first meeting are to review and discuss this Charter, review study activities, review and make recommendations about the protocol, review any potential conflicts of interest among board members, determine the frequency of interim meetings, and determine what data should be presented at subsequent meetings, including whether the data will or will not be masked to identity of randomized groups. The first meeting may occur any time before the first participant has completed treatment day 1.

Then, the DSMB will convene once the first participant has completed treatment day 1 or 6 months from the initial meeting, whichever comes first. The DSMB will then convene every 6 months and when needed to review limited cohort safety data as described in the data and safety monitoring plan. Meetings and conference calls will be scheduled by a study team member at Boston Children’s Hospital.

The agenda for DSMB meetings and calls will be drafted by the project manager but may also be completed by another study team member if coverage is required. The agenda and meeting materials should be distributed by the study team at least one week before each meeting. Each agenda should include as the first item, an inquiry about any new conflicts of interest.

At the beginning of each meeting, the project manager or delegated study team member will ask all DSMB members to state whether they have developed any new conflicts of interest since the last DSMB meeting. If a new conflict is reported, the Chair will determine if the conflict limits the ability of the DSMB member to participate in the discussion. The DSMB also will review adverse event data, other safety data, and enrollment data at each meeting to ensure proper trial conduct. At intervals, as noted above, the DSMB may also review formal interim analyses of the primary end point.

It is expected that all DSMB members will attend every meeting and call. All standing Board members are voting members. Quorum for voting requires a minimum of three standing members one of whom has to be the statistician. The Board may wish to decide if particular expertise is needed within the quorum for the meeting to be valid.

**5. Discussion of Confidential Material**

DSMB meetings and calls will be organized into open, closed, and executive sessions.

- During the **open sessions**, information will be presented to the DSMB by the study investigators and study statisticians, with time for discussion.
- Information on efficacy and safety by treatment group will be presented by the study statisticians during **closed sessions**.

The DSMB may elect to hold an **executive session** in which only the DSMB members are present in order to discuss study issues independently. If the executive session occurs on a conference call, steps will be taken to ensure that only the appropriate participants are on the call, and to invite others to re-join the call only at the conclusion of the executive session.

At the conclusion of the executive session, the DSMB Chair will provide a summary of the DSMB’s recommendations to the investigators, including the vote for continuation or discontinuation of the study and any recommendations for modifications of the study. This provides an opportunity for study investigators to ask questions to clarify the recommendations. The meeting is then adjourned.

**6. Reports to the DSMB (Open Reports)**

For each meeting, the Lead Study Statistician will work with the project manager to prepare summary reports and tables to facilitate the oversight role of the DSMB. The DSMB should discuss at the first or subsequent meetings what data the Board wishes to review and how those data should be presented. This report should include but not be limited to the following:

1. Trial Summary including a complete list of study personnel, a brief statement of the purpose of the trial, the projected timetable and the trial status.
2. Protocol amendments
3. Recruitment and Retention/Withdrawal
4. Baseline description of subjects
5. Compliance to protocol, deviations or violations
6. Adverse events summary
7. Type and severity
8. Relationship to the study intervention
9. Action Taken
10. Outcome
11. Reportable events summary

If the sponsor-investigator, study monitor, or appropriate regulatory officials discover conditions due to reasons including but not limited to futility or risk(s) to the participants that arise during the study, the study should be paused or halted. This action may be taken after appropriate consultation among the sponsor-investigator, developer/distributer of the investigational product, , study monitors and DSMB. The trigger point for AE and study pause: Severity Level 3 and 4 AEs will be reviewed by PI and determined if it is worthy of study pause. Any change in stool that is higher than baseline will be discussed on a patient to patient basis.

If the sponsor-investigator, study monitor, or appropriate regulatory officials discover conditions arising during the study that indicate that the study should be halted, this action may be taken after appropriate consultation among the sponsor-investigator, developer/distributer of the investigational product, study monitor, and DSMB. Conditions that may warrant termination of the study include, but are not limited to, the following:

- The discovery of an unexpected, serious, or unacceptable risk to the subjects enrolled in the study
- A decision on the part of the developer/distributer of the investigational product to suspend or discontinue testing, evaluation, or development of the product.

A study may also warrant termination under the following conditions:

- Failure of the investigator to enroll subjects into the study at an acceptable rate
- Failure of the investigator to comply with pertinent regulations of appropriate regulatory authorities
- Submission of knowingly false information from the site to the sponsor, study monitor, or appropriate regulatory authority
- Insufficient adherence to protocol requirements

**6. Reports of DSMB Deliberations**

**Formal minutes**

The project manager or other delegated study team member is responsible for producing the initial draft of the DSMB meeting minutes, including the recommendations. Ordinarily, these minutes will not include content or discussions from the closed or executive sessions. The initial draft will be circulated to the DSMB Chair for review and revision within 14 days of each meeting. These minutes are prepared to summarize the key points of the discussion, requests for additional information, response of the investigators to previous recommendations, and the recommendations from the current meeting. The DSMB Chair may sign the minutes or indicate approval electronically via email. The project manager or other delegated study team member will then distribute the final minutes to the study principal investigator and all other meeting attendees.

**Reports**

The DSMB Chair will prepare a report documenting Board recommendations and Board voting outcomes. The result of this vote will be to continue the trial without modification, continue with modifications (specified) or terminate the trial. The DSMB Chair will submit this report to the study principal investigator within 21 days of each meeting.

If the DSMB does not identify any safety or other protocol-related concerns, the DSMB Chair will prepare a Summary Report that will state that:

- a review of recruitment, outcome data, adverse events, and information relating to study performance (e.g., data timeliness, completeness, and quality) took place;
- the observed frequency of adverse events did not exceed what was expected and indicated in the informed consent;
- a review of recent literature relevant to the research took place and;
- the DSMB recommendation that the study was to either a) continue without modification of the protocol or informed consent, b) continue with modifications (specified), or c) discontinue the trial.

If concerns are identified, the report to the Principal Investigator will outline the concerns, the DSMB’s discussion of the concerns, and the basis for any recommendations that the DSMB has made in response to the concerns.

**8. Statistical Monitoring Guidelines**

At the first meeting, review of the protocol will include review of the statistical analysis plan. The

DSMB should discuss the adequacy of that plan and the statistical monitoring procedures they propose to follow to guide their recommendations about termination or continuation of the trial. These procedures could include guidelines for early termination for benefit, termination for futility, and termination for safety reasons.

**9. Confidentiality and Objectivity**

All materials, discussions and proceedings of the DSMB are completely confidential. Members and other participants in DSMB meetings are expected to maintain confidentiality. Closed session meeting materials should be destroyed in a secure manner (shredding) following each meeting.

In order to maintain their objectivity, DSMB members are expected not to discuss the study with the principal investigator except during DSMB meetings. Communication with DSMB members will be with the DSMB Chair through the study team or the principal investigator. It is expected that the principal investigator will not communicate with DSMB members about the study directly, except when making presentations or responding to questions at DSMB meetings.

DSMB Chair Member Signature: ______________________________ Date: ____________

DSMB Chair Member Printed Name: _________________________________

DSMB Member Signature: ____________________________________ Date: ____________

DSMB Member Printed Name: _________________________________

DSMB Member Signature: ____________________________________ Date: ____________

DSMB Member Printed Name: _________________________________

DSMB Member Signature: ____________________________________ Date: ____________

DSMB Member Printed Name: _________________________________
