# Supplementary material for: Fat digestion using RELiZORB in children with short bowel syndrome who are dependent on parenteral nutrition: Protocol for a 90-day, phase 3, open labeled study
Source: PLoS One. 2023 Mar 1;18(3):e0282248. doi: 10.1371/journal.pone.0282248 (PMC9977023; doi:10.1371/journal.pone.0282248)
Supplement: Peer review history

# Editor report: Decision Letter 0

**DOI:** 10.1371/journal.pone.0282248.r001

**Authors:** Richard Ali

13 Feb 2023

Fat digestion using RELiZORB in children with short bowel syndrome who are dependent on parenteral nutrition: Protocol for a 90-day, phase 3, open labeled study

PONE-D-22-35666

Dear Dr. Puder,

We’re pleased to inform you that your manuscript has been judged scientifically suitable for publication and will be formally accepted for publication once it meets all outstanding technical requirements.

Within one week, you’ll receive an e-mail detailing the required amendments. When these have been addressed, you’ll receive a formal acceptance letter and your manuscript will be scheduled for publication.

An invoice for payment will follow shortly after the formal acceptance. To ensure an efficient process, please log into Editorial Manager at http://www.editorialmanager.com/pone/, click the 'Update My Information' link at the top of the page, and double check that your user information is up-to-date. If you have any billing related questions, please contact our Author Billing department directly at authorbilling@plos.org.

If your institution or institutions have a press office, please notify them about your upcoming paper to help maximize its impact. If they’ll be preparing press materials, please inform our press team as soon as possible -- no later than 48 hours after receiving the formal acceptance. Your manuscript will remain under strict press embargo until 2 pm Eastern Time on the date of publication. For more information, please contact onepress@plos.org.

Kind regards,

Richard Ali

Staff Editor

PLOS ONE

Journal Requirements:

When submitting your revision, we need you to address these additional requirements.

1. Please ensure that your manuscript meets PLOS ONE's style requirements, including those for file naming. The PLOS ONE style templates can be found at 

https://journals.plos.org/plosone/s/file?id=wjVg/PLOSOne_formatting_sample_main_body.pdf and 

https://journals.plos.org/plosone/s/file?id=ba62/PLOSOne_formatting_sample_title_authors_affiliations.pdf

2. Thank you for stating the following in the Competing Interests section: 

"We have read the journal's policy and the authors of this manuscript have the following competing interests: Dr. Puder and Dr. Gura receive research support and advisory compensation from Alcresta Therapeutics, Inc (Newton, MA) which is the manufacturer of the device proposed in our study. Ms. Rudie has received advisory compensation from Alcresta Therapeutics. Dr. Loring and Dr. First were employees of Alcresta Therapeutics at the time of the protocol design. Dr. Martin receives research funding from Mead Johnson Nutrition and serves on the scientific advisory boards of LactaLogics, Plakous Therapeutics, and Vitara Biomedical. These competing interests do not alter our adherence to PLOS ONE policies on data sharing and materials.

We note that one or more of the authors are employed by a commercial company: Alcresta Therapeutics.

(1) Please provide an amended Funding Statement declaring this commercial affiliation, as well as a statement regarding the Role of Funders in your study. If the funding organization did not play a role in the study design, data collection and analysis, decision to publish, or preparation of the manuscript and only provided financial support in the form of authors' salaries and/or research materials, please review your statements relating to the author contributions, and ensure you have specifically and accurately indicated the role(s) that these authors had in your study. You can update author roles in the Author Contributions section of the online submission form.

Please also include the following statement within your amended Funding Statement. 

“The funder provided support in the form of salaries for authors [insert relevant initials], but did not have any additional role in the study design, data collection and analysis, decision to publish, or preparation of the manuscript. The specific roles of these authors are articulated in the ‘author contributions’ section.”

If your commercial affiliation did play a role in your study, please state and explain this role within your updated Funding Statement. 

(2) Please also provide an updated Competing Interests Statement declaring this commercial affiliation along with any other relevant declarations relating to employment, consultancy, patents, products in development, or marketed products, etc.  

Within your Competing Interests Statement, please confirm that this commercial affiliation does not alter your adherence to all PLOS ONE policies on sharing data and materials by including the following statement: ""This does not alter our adherence to  PLOS ONE policies on sharing data and materials.” (as detailed online in our guide for authors http://journals.plos.org/plosone/s/competing-interests).  If this adherence statement is not accurate and  there are restrictions on sharing of data and/or materials, please state these. Please note that we cannot proceed with consideration of your article until this information has been declared.

Please respond by return email with an updated Funding Statement and Competing Interests Statement and we will change the online submission form on your behalf.

3. Your ethics statement should only appear in the Methods section of your manuscript. If your ethics statement is written in any section besides the Methods, please move it to the Methods section and delete it from any other section. Please ensure that your ethics statement is included in your manuscript, as the ethics statement entered into the online submission form will not be published alongside your manuscript. 

# Editor report: Acceptance letter

**DOI:** 10.1371/journal.pone.0282248.r002

**Authors:** Richard Ali

20 Feb 2023

PONE-D-22-35666 

Fat digestion using RELiZORB in children with short bowel syndrome who are dependent on parenteral nutrition: Protocol for a 90-day, phase 3, open labeled study 

Dear Dr. Puder:

I'm pleased to inform you that your manuscript has been deemed suitable for publication in PLOS ONE. Congratulations! Your manuscript is now with our production department. 

If your institution or institutions have a press office, please let them know about your upcoming paper now to help maximize its impact. If they'll be preparing press materials, please inform our press team within the next 48 hours. Your manuscript will remain under strict press embargo until 2 pm Eastern Time on the date of publication. For more information please contact onepress@plos.org.

If we can help with anything else, please email us at plosone@plos.org. 

Thank you for submitting your work to PLOS ONE and supporting open access. 

Kind regards, 

PLOS ONE Editorial Office Staff

on behalf of

Richard Ali 

Staff Editor

PLOS ONE
